# Dynamic trend analysis of global psoriasis burden from 1990 to 2021: a study of gender, age, and regional differences based on GBD 2021 data

**DOI:** 10.3389/fpubh.2025.1518681

**Published:** 2025-07-09

**Authors:** Jikui Xiong, Tiankuo Xue, Meng Tong, Libo Xu, Bingxue Bai

**Affiliations:** Department of Dermatology, The Second Affiliated Hospital of Harbin Medical University, Harbin, China

**Keywords:** psoriasis, Global Burden of Disease, incidence, prevalence, disability-adjusted life years

## Abstract

**Background:**

Psoriasis is a chronic immune-mediated skin disease associated with systemic comorbidities such as cardiovascular disease and depression. While genetic susceptibility, immune dysregulation, and environmental factors are known contributors, the precise etiology remains uncertain. This study uses data from the Global Burden of Disease (GBD 2021) database to examine global and regional trends in psoriasis incidence, prevalence, and disability-adjusted life years (DALYs), analyzing differences by gender, age, and region to guide public health planning.

**Methods:**

Data from GBD 2021, covering 204 countries and regions, were used to estimate psoriasis incidence, prevalence, and DALYs through the DisMod-MR 2.1 model with Bayesian meta-regression to integrate multiple data sources. Annual average percentage change (AAPC) was calculated to analyze trends from 1990 to 2021, with future projections for the next 15 years based on a Bayesian age-period-cohort model.

**Results:**

From 1990 to 2021, global psoriasis prevalence increased from 23.06 million to 42.98 million (an 86% rise), and incidence grew by 80% from 2.85 million to 5.10 million cases. Psoriasis-related DALYs rose from 2 million to 3.69 million (an 85% increase). Regions with high Socio-demographic Index (SDI) values, including Western Europe, high-income North America, and Andean Latin America, exhibited significantly higher prevalence, incidence, and DALY rates compared to low-SDI regions such as East Asia and Sub-Saharan Africa. The burden was marginally higher in males than females, with the largest disparities in middle-aged males (40–60 years). A positive correlation between SDI and psoriasis burden was observed, showing that higher socioeconomic regions bear a greater disease burden.

**Conclusion:**

The global psoriasis burden continues to increase, particularly in high-SDI regions, where aging populations and greater healthcare access coexist. Projections indicate that psoriasis burden will rise over the next 15 years, especially among male patients. Enhanced early diagnosis, personalized treatment, and management of comorbidities are essential to reduce psoriasis’s long-term health impacts and improve patient outcomes.

## Introduction

Psoriasis is a chronic, recurrent, immune-mediated skin disease, characterized by symptoms such as erythema, scaling, itching, and pain (1–3). As the disease progresses, it can significantly impair patients’ quality of life, often leading to psychological issues, including anxiety and depression (4, 5). Although the exact cause of psoriasis remains uncertain, genetic susceptibility, immune dysregulation, and environmental factors are recognized as major contributing factors (6, 7). Recent advances in molecular immunology and genetics have established psoriasis as a systemic disease, not confined to skin lesions, but closely linked with comorbidities such as cardiovascular diseases, metabolic disorders (including diabetes), and depression (8, 9). This complex pathology exacerbates the disease burden and complicates treatment.

The Global Burden of Disease (GBD) project provides essential baseline data for assessing the global and regional burden of psoriasis. By systematically analyzing incidence, mortality, and disability-adjusted life years (DALYs), GBD highlights the health impact of psoriasis across various regions and populations (10). The GBD 2021 database, developed by the Institute for Health Metrics and Evaluation, provides comprehensive epidemiological data on 371 diseases across 204 countries from 1990 to 2021, integrating diverse data sources using standardized methods like the DisMod-MR 2.1 model. This robust framework has been widely applied to study the burden of chronic diseases, including immune-mediated conditions like psoriasis and systemic diseases such as female-specific cancers (11).

This study aims to leverage the most recent Global Burden of Disease (GBD) 2021 database to provide a comprehensive analysis of psoriasis burden across global, regional, and national levels. By examining incidence, prevalence, and DALYs from 1990 to 2021 and projecting future trends, we seek to uncover epidemiological shifts across gender, age groups, and regions. Although prior studies have assessed psoriasis using GBD 2017 and GBD 2019 data, and some recent works have used GBD 2021 data for specific countries, our study is the first to present a global and regional assessment using GBD 2021 data with detailed stratification by sex, age, and SDI. Additionally, we incorporate Bayesian age-period-cohort (BAPC) modeling to forecast future disease burden, offering new insights into emerging patterns and high-burden areas. These findings aim to support evidence-based public health planning and targeted psoriasis management strategies worldwide. Although prior studies have assessed psoriasis using GBD 2017 and GBD 2019 data, and some recent works have used GBD 2021 data for specific countries, our study is the first to present a global and regional assessment using GBD 2021 data with detailed stratification by sex, age, and SDI. Additionally, we incorporate Bayesian age-period-cohort modeling to forecast future disease burden, offering new insights into emerging patterns and high-burden areas.

## Methods

### Data collection

This study utilized the GBD 2021 database, which comprehensively evaluated health losses associated with 369 diseases, injuries, and conditions, as well as 88 risk factors across 204 countries and regions, including psoriasis (10). The GBD database incorporates advanced epidemiological data and standardized statistical methods to adjust for missing data and confounding factors. Specific details regarding the GBD study design and methodology can be found in the relevant literature (11). Data collection and analysis for this study followed the Guidelines for Accurate and Transparent Health Estimates Reporting (GATHER), and informed consent was not required, as the data were sourced from publicly available databases (12).

### Estimation framework

Advanced modeling techniques were employed in this study to estimate the prevalence, incidence, and DALYs for psoriasis. The DisMod-MR 2.1 model, a Bayesian meta-regression tool, was used to integrate multiple data sources and handle disease parameters, epidemiological relationships, and geographic data, ensuring robust estimates even in the presence of missing data (10). DALYs, as a measure of disease burden, capture the contribution of specific risk factors and are calculated as the sum of years of life lost (YLLs) and years lived with disability (YLDs) (10).

### Future trend prediction

Future trends were predicted using a BAPC model, combined with the integrated nested Laplace approximation method. Studies have demonstrated that the BAPC model offers superior accuracy and coverage compared to other prediction methods (13, 14). All analyses and visualizations were conducted using the World Health Organization’s Health Equity Assessment Toolkit and R statistical software (version 4.4.0).

### Statistical analysis

The 25th and 975th percentiles were used to derive 95% uncertainty intervals (UIs) following GBD’s methodology. The study analyzed global trends in incidence, mortality, and DALYs for psoriasis. To assess trends in age-standardized rates (ASRs), the study utilized the estimated annual percentage change (AAPC). AAPC and its 95% confidence intervals (CIs) were calculated using linear regression, with the logarithmic transformation of rates as the dependent variable and year as the independent variable. AAPC represents the geometric weighted average of annual percentage changes (APCs) over a fixed interval. For instance, an AAPC of 0.1 indicates a 0.1% annual growth. AAPCs and their 95% CIs were used to identify trend changes. We calculated AAPCs from 1990 to 2021 and employed Pearson correlation coefficients to assess the relationship between SDI and age-standardized incidence rates for psoriasis brain and central nervous system cancers. All statistical analyses in this study were descriptive and based on publicly available GBD 2021 estimates; no new statistical modeling was performed.

## Results

### Global level

**Figure 1:**
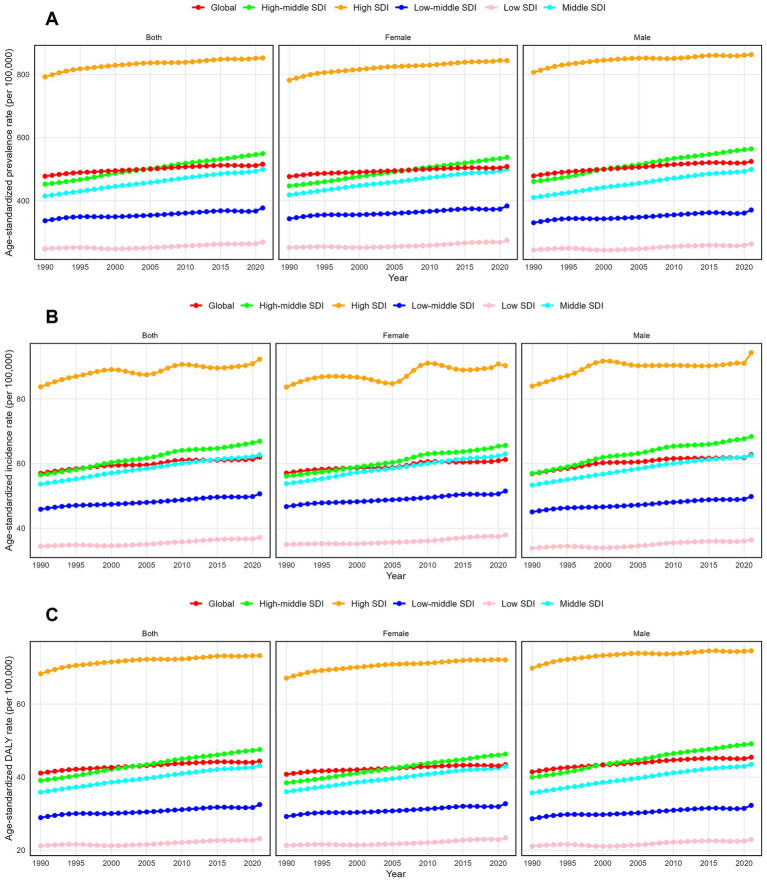
Trends in psoriasis prevalence, incidence and disability-adjusted life-years from 1990 to 2021. **(A)** Prevalence rate. **(B)** Incidence rate. **(C)** DALYs rate.

Globally, psoriasis cases increased by approximately 86% from 1990 to 2021, rising from 23.06 million to 42.98 million. The ASPR increased from 477.7 per 100,000 in 1990 to 516.0 per 100,000 in 2021, with an average annual growth of 0.24% (95% CI: 0.23 to 0.25%) (Table 1 and Figure 1A). In 2021, new psoriasis cases grew from 2.85 million in 1990 to 5.10 million, an increase of 80% over 32 years. The ASIR rose from 57.0 per 100,000 in 1990 to 62.0 per 100,000 in 2021, with an annual average growth of 0.27% (95% CI: 0.26 to 0.28%) (Supplementary Table 1 and Figure 1B). In 2021, psoriasis-related DALYs reached 3.69 million, compared to 2 million in 1990, an 85% increase. The age-standardized DALY rate rose from 41.1 per 100,000 in 1990 to 44.4 per 100,000 in 2021, with an annual increase of 0.24% (95% CI: 0.23 to 0.25%) (Supplementary Table 2 and Figure 1C).

**Table 1:** Age-standardized prevalence rate and AAPC of psoriasis globally and by region from 1990 to 2021.

Location	1990		2021		AAPC (95%CI)
	Number (95%UI)	ASR (95%UI)	Number (95%UI)	ASR (95%UI)	
Global	2852675.8 (2764294.4–2943367.2)	57.0 (55.3–58.8)	5099418.3 (4945748.1–5254031.0)	62.0 (60.1–63.9)	0.27 (0.26–0.28)
High SDI	777403.0 (754267.8–801779.7)	83.7 (81.2–86.3)	1134087.5 (1099752.4–1167976.4)	92.3 (89.6–94.9)	0.3 (0.28–0.31)
High-middle SDI	606238.5 (587340.7–625371.2)	56.5 (54.8–58.3)	1008069.6 (975952.4–1040169.5)	66.9 (64.8–69.0)	0.54 (0.54–0.55)
Middle SDI	843818.8 (816665.9–871346.7)	53.7 (52.1–55.3)	1641222.1 (1590306.0–1691699.4)	62.7 (60.8–64.7)	0.5 (0.49–0.5)
Low-middle SDI	475261.2 (459036.1–489690.4)	45.9 (44.4–47.3)	947741.1 (916034.2–979472.8)	50.7 (49.0–52.3)	0.31 (0.29–0.32)
Low SDI	146976.2 (141645.3–151520.3)	34.4 (33.3–35.6)	363918.8 (350683.3–375676.6)	37.2 (35.9–38.4)	0.24 (0.22–0.25)
High-income Asia Pacific	98706.2 (95548.7–101802.6)	52.7 (51.0–54.3)	126176.7 (121983.0–130129.5)	56.7 (55.0–58.5)	0.22 (0.2–0.23)
High-income North America	279448.7 (271343.3–288121.8)	96.5 (93.6–99.4)	422371.3 (410310.8–434102.1)	103.5 (100.8–106.1)	0.23 (0.21–0.24)
Western Europe	430713.0 (417260.5–444548.1)	103.7 (100.4–107.0)	565866.3 (547560.0–584609.2)	115.3 (111.8–118.9)	0.32 (0.3–0.33)
Australasia	14069.9 (13562.3–14613.9)	66.6 (64.1–69.1)	26079.6 (25106.4–27050.0)	75.9 (73.2–78.7)	0.41 (0.39–0.42)
Andean Latin America	31948.6 (30728.0–33078.5)	92.3 (88.8–95.5)	69315.2 (66699.9–71992.7)	105.0 (101.1–109.1)	0.42 (0.41–0.42)
Tropical Latin America	81446.9 (78601.2–83874.8)	57.5 (55.7–59.3)	138658.9 (134234.8–143056.3)	57.8 (55.9–59.5)	0.02 (0.01–0.02)
Central Latin America	106949.4 (102905.0–110561.0)	72.8 (70.4–75.3)	203443.3 (196311.4–210198.6)	78.6 (75.9–81.2)	0.25 (0.25–0.26)
Southern Latin America	33084.1 (31783.9–34406.3)	67.7 (65.1–70.4)	55542.8 (53414.8–57531.3)	76.9 (73.9–79.7)	0.39 (0.37–0.41)
Caribbean	24182.0 (23237.2–25081.9)	72.9 (70.0–75.5)	37556.8 (36151.9–38986.4)	76.4 (73.5–79.2)	0.16 (0.15–0.16)
Central Europe	73853.9 (71481.4–76385.4)	55.2 (53.4–57.0)	81011.8 (78559.2–83476.2)	61.5 (59.8–63.3)	0.35 (0.35–0.36)
Eastern Europe	128895.5 (124591.3–133152.6)	52.9 (51.2–54.7)	139137.5 (134768.5–143725.4)	58.6 (56.8–60.6)	0.34 (0.33–0.34)
Central Asia	32184.2 (31013.2–33420.1)	50.7 (48.9–52.6)	54029.3 (52019.6–56016.5)	55.7 (53.6–57.8)	0.31 (0.3–0.31)
North Africa and Middle East	152645.4 (147189.4–157649.6)	52.7 (50.9–54.4)	395336.5 (381884.7–409179.7)	64.0 (61.8–66.1)	0.63 (0.63–0.63)
South Asia	453226.3 (438163.6–467391.2)	45.4 (44.0–46.8)	910977.4 (881351.7–941591.2)	49.4 (47.8–51.0)	0.25 (0.22–0.28)
Southeast Asia	236373.2 (228543.3–244458.6)	58.0 (56.2–59.9)	491723.9 (475357.4–507614.6)	67.7 (65.5–69.8)	0.5 (0.5–0.5)
East Asia	543655.0 (526680.4–562339.1)	47.0 (45.5–48.5)	1037234.5 (1003142.1–1069796.6)	59.1 (57.2–60.9)	0.74 (0.74–0.75)
Oceania	2570.5 (2468.2–2666.8)	47.5 (45.6–49.3)	6516.1 (6251.8–6776.2)	52.8 (50.6–54.9)	0.34 (0.34–0.35)
Western Sub-Saharan Africa	62700.0 (60472.5–64604.8)	37.5 (36.3–38.7)	181649.8 (175025.0–187328.1)	42.2 (40.8–43.5)	0.38 (0.37–0.38)
Eastern Sub-Saharan Africa	34537.0 (33240.0–35702.1)	22.2 (21.4–22.9)	82617.8 (79425.4–85440.9)	22.9 (22.1–23.6)	0.1 (0.09–0.1)
Central Sub-Saharan Africa	16616.2 (15928.6–17255.6)	35.1 (33.8–36.4)	47617.0 (45582.5–49512.9)	39.3 (37.7–40.8)	0.36 (0.36–0.37)
Southern Sub-Saharan Africa	14869.8 (14319.5–15350.3)	31.8 (30.7–32.9)	26555.9 (25626.7–27433.8)	33.8 (32.6–34.9)	0.19 (0.18–0.19)

ASR, age-standardized rate; AAPC, average annual percentage change; CI, confidence interval; UI, uncertainty interval.

### Regional level

**Figure 2:**
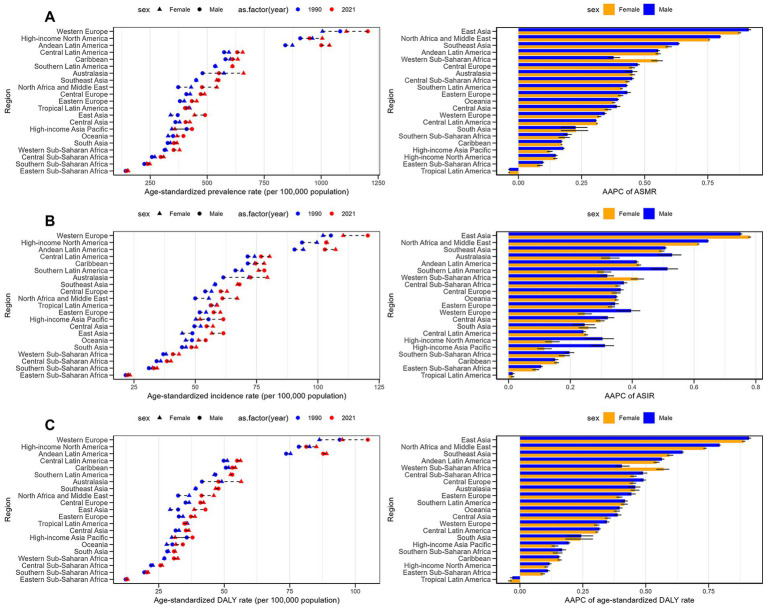
Age-standardized prevalence, incidence and DALY rates for psoriasis and their trends by sex and across 21 regions between 1990 and 2021. **(A)** Prevalence rate. **(B)** Incidence rate. **(C)** DALYs rate.

The burden of psoriasis varied significantly across regions with different SDI levels. In 2021, the ASPR showed marked disparities, with the highest rates in high-SDI regions at 852.3 per 100,000 (95% uncertainty interval: 830.1 to 875.3) and the lowest in low-SDI regions at 268.9 per 100,000 (95% uncertainty interval: 260.5 to 277.4) (Table 1 and Figure 1A). Furthermore, the ASPR increased most significantly in middle-high SDI regions, with an AAPC of 0.63% (95% CI: 0.63%) (Table 1 and Supplementary Figure 1A). In contrast, high-SDI regions experienced the smallest ASPR increase, with an AAPC of 0.23% (95% CI: 0.23 to 0.24%) (Table 1 and Supplementary Figure 1A). In 2021, the highest ASIR was observed in high-SDI regions, while the lowest was in low-SDI regions. Specifically, ASIR in high-SDI regions was 92.3 per 100,000 (95% UI: 89.6 to 94.9), while it was 37.2 per 100,000 (95% UI: 35.9 to 38.4) in low-SDI regions (Supplementary Table 1 and Figure 1B). Middle-high SDI regions exhibited the largest annual increase in ASIR, with an AAPC of 0.54% (95% CI: 0.54 to 0.55%) (Supplementary Table 1 and Supplementary Figure 1B), while low-SDI regions showed the smallest increase at 0.24% (95% CI: 0.22 to 0.25%) (Supplementary Table 1 and Supplementary Figure 1B). In 2021, the highest age-standardized DALY rate was 73.3 per 100,000 in high-SDI regions (95% UI: 53.2 to 97.7), while the lowest rate was 23.1 per 100,000 in low-SDI regions (95% UI: 16.7 to 30.9) (Supplementary Table 2 and Figure 1C). The largest annual DALY increase occurred in middle-high SDI regions, with an AAPC of 0.64% (95% CI: 0.63 to 0.64%), while the smallest increase was in high-SDI regions, with an AAPC of 0.22% (95% CI: 0.22 to 0.23%) (Figure 2; Supplementary Table 2; Supplementary Figure 1C).

This study evaluated the psoriasis burden across 21 GBD regions, revealing significant differences in burden by region and gender (Figures 2A–C). Western Europe, high-income North America, and Andean Latin America had higher ASPR, ASIR, and age-standardized DALY rates. Although the overall psoriasis burden was slightly higher in men, in certain regions, women had higher prevalence, incidence, and DALY rates, particularly in East and South Asia. AAPC data showed that the psoriasis burden increased most rapidly in East Asia, North Africa, and the Middle East, with significant growth in both genders. These findings highlight the need for region-specific research and tailored public health interventions.

### National level

**Figure 3:**
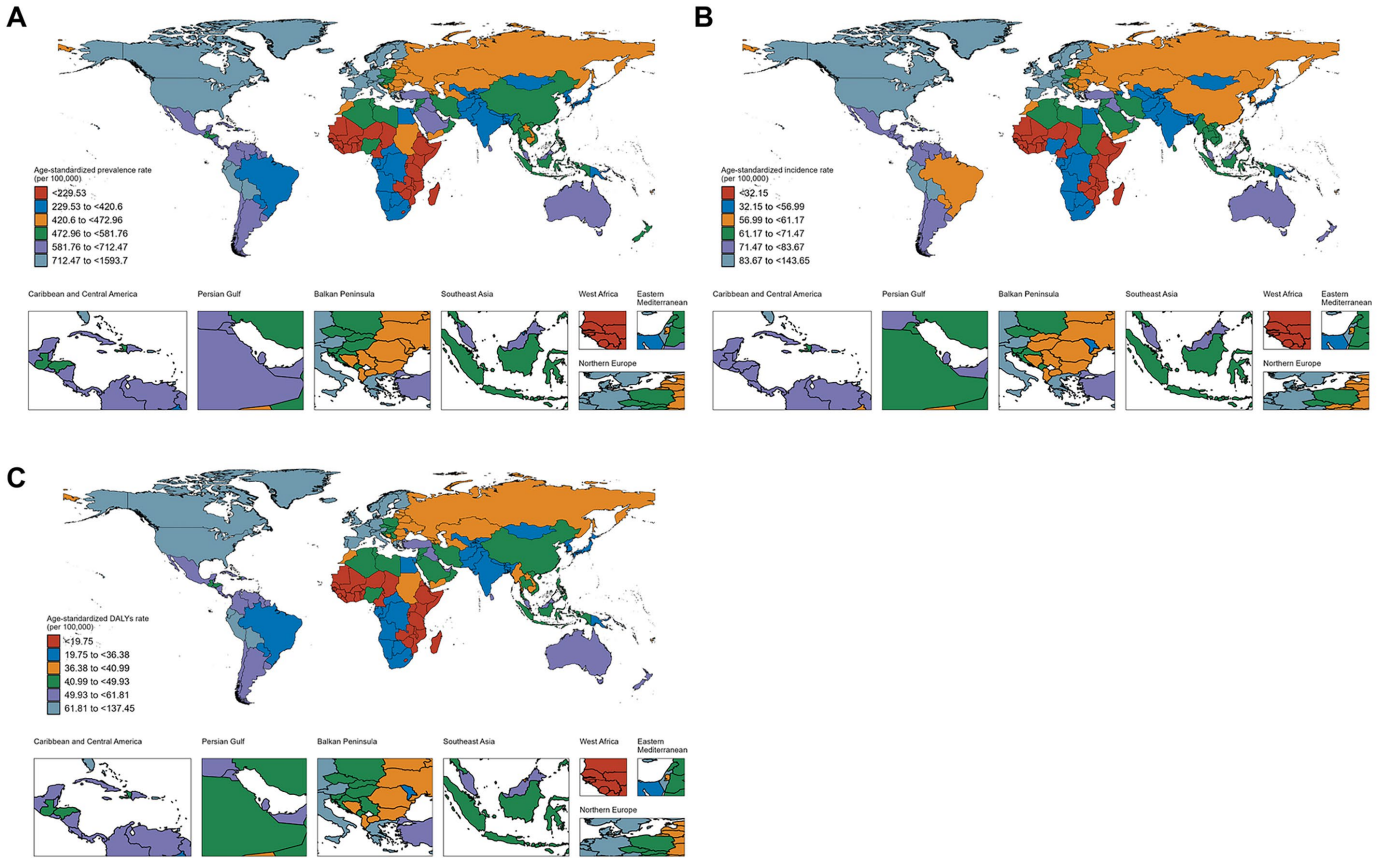
Global burden of psoriasis by sex in 204 countries and regions in 2021. **(A)** Prevalence rate. **(B)** Incidence rate. **(C)** DALYs rate.

In 2021, Germany (1,593.7 per 100,000) and Switzerland (1,299.1 per 100,000) had the highest ASPR, while Somalia (114.9 per 100,000) and Rwanda (117.0 per 100,000) had the lowest (Supplementary Table 3 and Figure 3A). Equatorial Guinea (AAPC: 1.34%) and Oman (AAPC: 0.96%) experienced the largest ASPR increases, while Somalia (AAPC: −0.26%) and South Sudan (AAPC: −0.18%) saw the largest decreases (Supplementary Table 3 and Supplementary Figure 1A). In 2021, Germany (143.6 per 100,000) and Switzerland (124.8 per 100,000) had the highest ASIR, while Somalia (18.6 per 100,000) and Rwanda (18.9 per 100,000) had the lowest (Supplementary Table 4 and Figure 3B). Equatorial Guinea (AAPC: 0.97%) and Oman (AAPC: 0.76%) showed the largest increases in ASIR, while Somalia (AAPC: −0.2%) and South Sudan (AAPC: −0.1%) experienced the largest decreases (Supplementary Table 4 and Supplementary Figure 1B). In 2021, Germany (137.4 per 100,000) and Switzerland (112.1 per 100,000) had the highest DALY rates, while Somalia (10.0 per 100,000) and Rwanda (10.2 per 100,000) had the lowest (Supplementary Table 5 and Figure 3C). Equatorial Guinea (AAPC: 1.35%) and Oman (AAPC: 0.97%) experienced the largest increases in DALY rates, while Somalia (AAPC: −0.24%) and Burundi (AAPC: −0.17%) had the largest decreases (Supplementary Table 5 and Supplementary Figure 1C).

### Age-sex patterns

**Figure 4:**
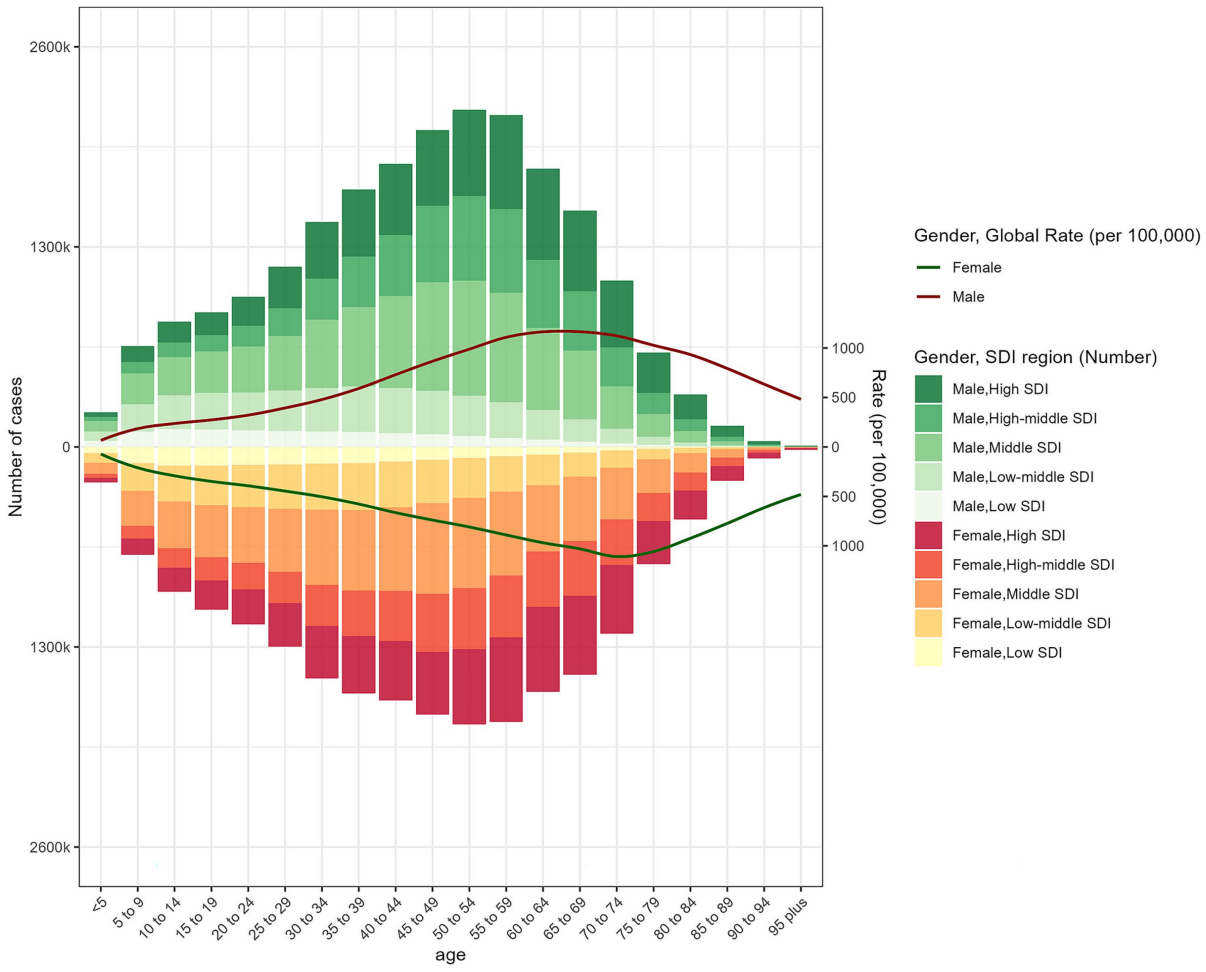
The age-specific numbers and ASPR of psoriasis by SDI regions in 2021.

**Figure 5:**
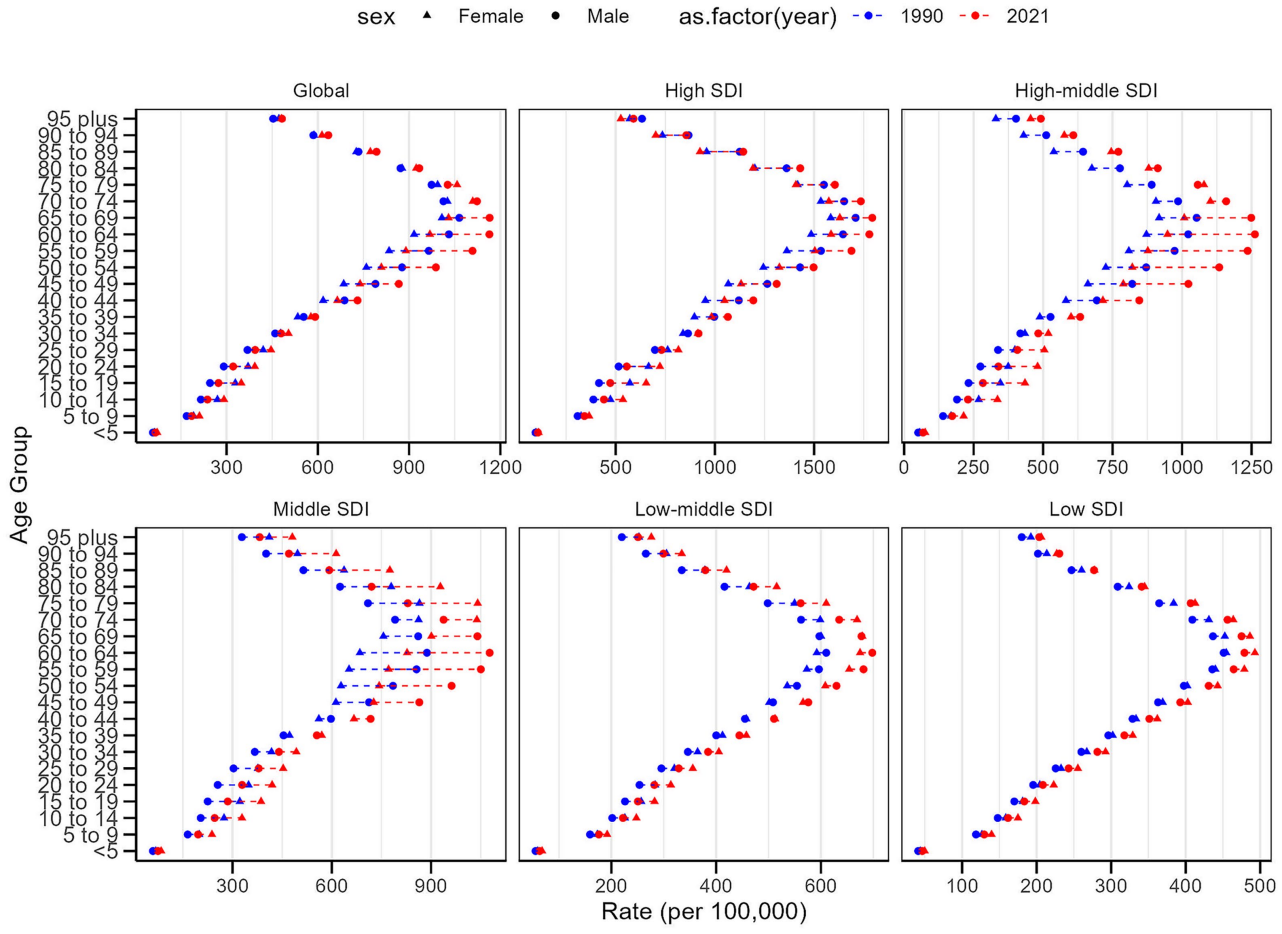
Age-standardized prevalence rates of psoriasis by sex, age group, and socio-demographic index, 1990 and 2021.

In 2021, the prevalence of psoriasis exhibited a trend of increasing with age before declining, peaking between the ages of 40 and 60, and then gradually decreasing. During childhood and adolescence, the prevalence was relatively low, with fewer cases observed among individuals aged 0 to 20 years (Figure 4). Overall, there was no significant difference in prevalence between males and females, but the disparity was more pronounced in certain age groups, particularly between the ages of 45 and 69. Moreover, the gender gap was more evident in high-middle SDI and middle SDI regions. Among individuals aged 60 and above, the prevalence declined for both genders but remained at relatively high levels (Figure 5). The highest prevalence rates and number of cases were observed in regions with a high Socio-demographic Index (SDI), while regions with a low SDI had fewer cases, with the 40 to 60 age group being the peak period across all SDI groups (Figure 4). The age-standardized DALY rates followed a similar pattern to the age-standardized prevalence rates (Supplementary Figures 4, 5). The age-standardized incidence rate and DALYs among individuals aged 15–24 remained relatively stable from 1990 to 2021, with only minor regional variations across different SDI levels.

In 2021, the age-standardized incidence rates and the number of psoriasis cases increased with age, peaking between the ages of 30 and 60, and then gradually declining. A significant gender difference in incidence rates was observed between the ages of 35 and 79, especially in the 55–59 age group. This gender gap was further amplified in the corresponding age groups in high-middle SDI and middle SDI regions. This suggests that socioeconomic factors may influence the incidence and gender disparity of psoriasis (Supplementary Figures 2, 3).

### Association with the sociodemographic index

**Figure 6:**
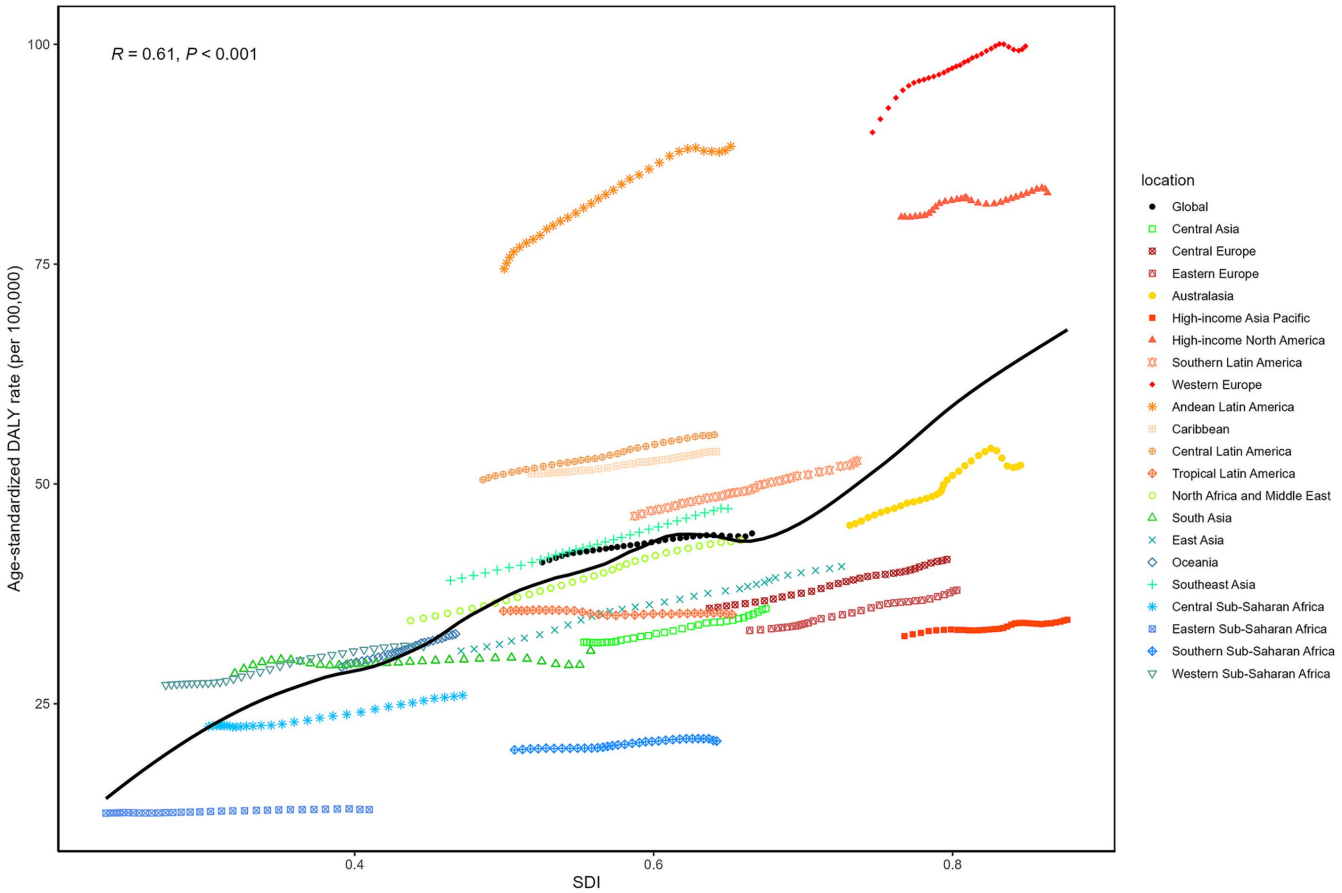
From 1990 to 2021, the age-standardized DALY rates for psoriasis across 21 Global Burden of Disease regions, classified by the SDI, are illustrated with 32 points for each region, representing the observed age-standardized DALY rates in that region from 1990 to 2021.

A significant positive correlation exists between the SDI and DALYs due to psoriasis (*R* = 0.61, *p* < 0.001) (Figure 6). This indicates that as SDI increases, DALYs associated with psoriasis also rise. High-SDI regions, including high-income areas in North America, East Asia, and Australia, exhibit significantly higher age-standardized DALY rates than low-SDI regions, such as Sub-Saharan Africa and South Asia. Despite the availability of better medical and social resources in high-SDI regions, the psoriasis disease burden remains substantial, especially in terms of DALYs. Additionally, in medium-SDI regions, such as Eastern Europe and Central Asia, age-standardized DALY rates are relatively high, while low-SDI regions exhibit lower DALY burdens. This trend may reflect that although patients in high- and middle-income countries tend to have higher survival rates, the impact of psoriasis on their quality of life is more pronounced, leading to elevated DALY values. Overall, the psoriasis DALY burden increases with socioeconomic development, likely influenced by lifestyle factors, environmental conditions, healthcare systems, and diagnostic rates.

Data from 204 countries in 2021 (Supplementary Figure 6) further support the significant positive correlation between psoriasis DALYs and SDI (*R* = 0.69, *p* < 0.001). As SDI rises, the DALY burden of psoriasis also increases. High-income countries, such as Monaco, Switzerland, Germany, and Luxembourg, report significantly higher DALYs than low- and middle-income countries. This may be due to higher diagnosis rates, longer patient survival, and better utilization of healthcare resources in developed countries. Conversely, low-SDI regions, such as Sub-Saharan Africa and South Asia, demonstrate a relatively lower DALY burden, which may be due to limited healthcare resources, lower diagnosis rates, and potentially higher numbers of undiagnosed cases. The overall trend shows that as SDI rises, the psoriasis DALY burden increases in middle- and high-SDI countries, while it remains relatively low in low-SDI countries. Variations in DALY burden within the same SDI range suggest that factors beyond SDI, including socioeconomic conditions, environmental factors, healthcare systems, and lifestyle, also significantly influence the psoriasis disease burden.

### Fifteen-year global psoriasis burden forecast

**Figure 7:**
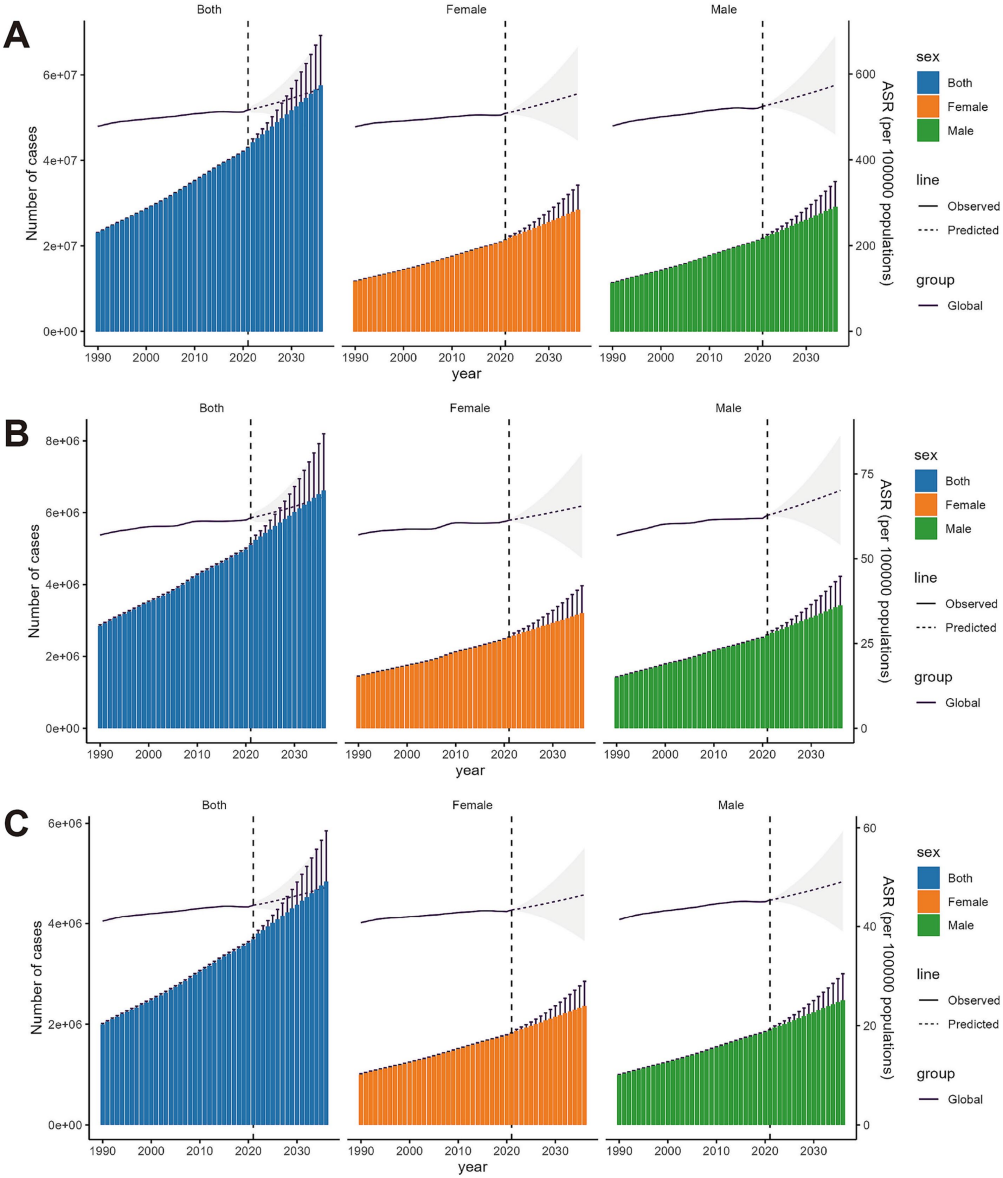
BAPC model predictions for the prevalence, incidence and DALY rates of psoriasis over the next 15 years, with both case numbers and age-standardized rates. The shaded areas represent the 95% confidence intervals. **(A)** Prevalence rate. **(B)** Incidence rate. **(C)** DALYs rate.

Over the next 15 years (2022–2036), global psoriasis prevalence, incidence, and DALY burden are projected to increase significantly. Figure 7A shows that the number of psoriasis cases will continue to rise, with a slightly higher increase in men compared to women, while the global ASPR is expected to remain relatively stable. Figure 7B indicates a similar trend in incidence, with new cases projected to grow steadily. The rate of increase in men is expected to slightly outpace that in women, but the global ASIR is projected to remain stable with minimal changes. Figure 7C illustrates the psoriasis-related DALY burden, which shows a larger increase in men than women. However, the growth rate of the global age-standardized DALY rate is expected to remain slow, indicating that while the psoriasis burden will increase over time, the rate of growth will be limited. Overall, this increase in disease burden is likely to be driven by global population growth and aging, rather than a substantial rise in age-standardized rates.

## Discussion

This study, using data from the GBD 2021 database, systematically assessed global trends in psoriasis prevalence, incidence, and disease burden from 1990 to 2021, focusing on variations across gender, age, and regions. Despite the unclear etiology of psoriasis, both its incidence and disease burden have increased globally. This trend underscores psoriasis as a chronic, recurrent, immune-mediated disease that poses a significant public health challenge, while offering insights for developing future targeted intervention policies. The following sections discuss the study’s main findings, analyzing potential contributing factors and offering recommendations for future disease management and public health interventions.

The results show a significant rise in the global burden of psoriasis from 1990 to 2021. The number of affected individuals grew from 23.06 million in 1990 to 42.98 million in 2021, an 86% increase, while DALYs attributable to the disease rose by 85%. Although the annual growth rates of age-standardized incidence and prevalence were relatively modest at 0.27 and 0.24%, respectively, these increases are statistically significant. This suggests that the global rise in psoriasis prevalence and incidence is independent of population growth, reflecting an increasing global health burden and indicating the need for enhanced prevention and management strategies.

Several factors may be driving the global increase in psoriasis burden. Environmental factors such as urbanization, air pollution, and lifestyle changes (e.g., Westernized diets, increased smoking, and rising obesity rates) likely contribute to this trend (15, 16). Psoriasis, as an immune-mediated disease, has a complex pathogenesis linked to genetic susceptibility and immune dysregulation. Recent advances in genomics and immunology have revealed the genetic underpinnings of psoriasis, indicating that certain populations may be at higher genetic risk (17). However, genetic factors alone cannot fully account for the global rise in disease burden; environmental and socioeconomic factors must also be considered.

This study also highlights gender and age differences in psoriasis burden. While the overall burden is similar between men and women, significant differences exist in specific age groups, particularly among middle-aged men (40–60 years). Our findings indicate a stable psoriasis burden among young adults (15–24 years) from 1990 to 2021, with minimal changes in age-standardized incidence and DALY rates across regions. In contrast, the burden peaks in middle-aged individuals (40–60 years), particularly males, with prevalence and incidence rates highest in high-middle and middle SDI regions. These age-specific patterns underscore the need for targeted interventions in middle-aged populations. These gender differences likely stem from hormonal levels, lifestyle variations, social pressures, and differing health-seeking behaviors (18). Moreover, the study identified a strong correlation between psoriasis prevalence and age, with the highest prevalence among middle-aged individuals (40–60 years). This correlation may reflect increased physiological stress and metabolic disorders in this age group (19). Additionally, the chronic, recurrent nature of psoriasis leads to greater disease burden during middle age due to exacerbations or relapses. With aging, weakened immune systems may reduce symptom severity, explaining the lower prevalence in older adults. However, older psoriasis patients face higher risks of comorbidities such as cardiovascular and metabolic disorders, emphasizing the need for a multidisciplinary approach in managing older adult patients (20).

Another significant finding is the marked regional variation in psoriasis burden, particularly across regions with differing SDI levels. The burden is higher in high-SDI regions, such as Western Europe, high-income North America, and Andean Latin America, with greater prevalence, incidence, and DALY rates than in low-SDI regions. This disparity may be explained by higher diagnostic rates in high-SDI regions, consistent with previous studies (21). Lifestyle factors, including poor dietary habits and environmental stressors (e.g., pollution), likely contribute to the increased incidence in high-income countries. Despite better healthcare resources in these regions, the chronic, recurrent nature of psoriasis, combined with its complex pathogenesis, makes long-term management more challenging (3). In contrast, the burden of psoriasis is lower in low-SDI regions, particularly in Sub-Saharan Africa and South Asia. This may reflect lower diagnostic rates and inadequate healthcare resources, as healthcare systems in these areas may lack sufficient knowledge and diagnostic capacity for psoriasis, resulting in many undiagnosed or untreated cases (22). High-SDI regions, such as Western Europe, high-income North America, and Andean Latin America, exhibited significantly higher prevalence, incidence, and DALY rates compared to low-SDI regions like Sub-Saharan Africa and South Asia. Notably, Andean Latin America’s elevated burden suggests region-specific factors, such as improved diagnostic capacity, may contribute to higher reported rates, warranting further investigation. These findings highlight the need for region-specific research and tailored public health interventions. Patients in low-income countries may die earlier from other prevalent diseases, such as infections, reducing the reported psoriasis burden, though many cases likely remain undiagnosed. Future public health efforts should focus on improving psoriasis surveillance and early diagnosis in low-SDI regions to enhance access to disease prevention and control.

This study further examined the relationship between socioeconomic factors and the burden of psoriasis. We found a positive correlation between the age-standardized DALY rate of psoriasis and SDI, indicating that higher socioeconomic levels are associated with increased DALY burden. Firstly, although high-SDI regions benefit from greater medical resources, the chronic nature of psoriasis, along with its associated comorbidities—such as cardiovascular disease, diabetes, and depression—often results in long-term health challenges for patients (23). Higher survival rates in these regions amplify the impact of psoriasis on quality of life, contributing to elevated DALYs. Furthermore, lifestyle factors in high-SDI regions, including high-stress environments, unhealthy diets rich in fats, and sedentary lifestyles, may exacerbate the risk and severity of psoriasis (16). Our analysis identified a significant positive correlation between SDI and DALYs (*R* = 0.61, *p* < 0.001), with high-SDI regions like Western Europe and high-income North America reporting DALY rates up to 73.3 per 100,000, compared to 23.1 per 100,000 in low-SDI regions. This trend likely reflects higher diagnostic rates and longer survival in high-SDI areas, which increase the reported burden despite better healthcare access. In contrast, lower psoriasis burdens in low-SDI regions may not reflect lower disease incidence, but rather inadequate medical resources, which result in underdiagnosis and lack of appropriate treatment. Patients in low-SDI regions face significant healthcare barriers, including limited awareness, insufficient infrastructure, and restricted access to medications (24), leading to underreported disease burden. Consequently, future research and public health initiatives should focus on improving diagnostic rates and treatment accessibility in low-income regions to alleviate the health burden on patients.

This study also utilized GBD data to project the future burden of psoriasis over the next 15 years. The findings show that global prevalence, incidence, and DALY burden of psoriasis will rise significantly, driven by population growth and aging. Although ASPR and ASIR are expected to remain relatively stable, population growth and aging will continue to drive an increase in the overall burden of psoriasis. This increase will be more pronounced in men, particularly middle-aged men, with a clear upward trend. The BAPC model predicts a continued rise in global psoriasis prevalence, incidence, and DALYs over the next 15 years (2022–2036), with a slightly higher increase in males, particularly middle-aged men. While age-standardized rates are expected to remain stable, population growth and aging will drive this increase. These findings underscore the need for targeted public health strategies, including early diagnosis and comorbidity management, to mitigate the growing burden. Future public health policies should prioritize early diagnosis and long-term management of psoriasis. As a chronic immune-mediated disease, psoriasis increases the risk of comorbidities, such as cardiovascular disease, metabolic disorders, and depression, necessitating multidisciplinary collaboration across dermatology, cardiology, endocrinology, and psychology (25–27). Moreover, given the profound impact of psoriasis on patients’ quality of life, future treatment strategies should emphasize psychological support and efforts to enhance life quality. This requires optimizing biological therapies, improving patient education, and establishing self-management support systems (28). Additionally, this study highlights the heterogeneity in the global burden of psoriasis, with significant regional and gender differences. Public health strategies must be adaptable and region-specific, with high-SDI regions focusing on long-term management and psychological support, and low-SDI regions prioritizing early diagnosis and improved access to care. By implementing these targeted interventions, the global burden of psoriasis can be significantly reduced.

Despite employing advanced modeling techniques and statistical analyses, this study has certain limitations. First, the GBD database relies on existing epidemiological data, and in some regions, the available data may be incomplete or of lower quality, potentially affecting the accuracy of the estimates. Although the DisMod-MR 2.1 model integrates multiple data sources to provide robust estimates, the precision of these estimates depends on the completeness and quality of the data, particularly in low-income regions where the incidence and burden of psoriasis may be underreported. Second, the data analysis in this study was based on publicly available databases and did not account for regional differences in medical resources, cultural factors, or health-seeking behaviors, all of which may influence psoriasis diagnosis and management. Additionally, the GBD estimation framework relies on standardized covariates such as the Socio-demographic Index (SDI), which may not fully capture region-specific determinants of disease burden. For example, the consistently high psoriasis burden in Andean Latin America—alongside elevated burdens for unrelated diseases like type 2 diabetes—suggests that unmodeled contextual factors may contribute. These findings underscore the need for further refinement of modeling approaches in regions with persistent burden discrepancies.

## Conclusion

This study, based on the GBD 2021 database, comprehensively assessed global trends in psoriasis prevalence and disease burden, with detailed analysis of gender, age, and regional differences. The findings indicate that the global burden of psoriasis continues to rise, especially among middle-aged men and in high-SDI regions. Despite the availability of advanced medical resources in these regions, psoriasis patients still face substantial long-term health challenges, underscoring the need for more robust disease management and psychological support. In contrast, the burden of psoriasis remains lower in low-SDI regions, though underdiagnosis is likely an issue. Public health efforts should focus on enhancing disease surveillance and early intervention in these areas. As the global population continues to grow and age, the burden of psoriasis is expected to further increase. Countries must proactively develop strategies that incorporate personalized management, multidisciplinary collaboration, and targeted policy interventions to effectively reduce the global burden of psoriasis and improve patients’ quality of life.

## Data Availability

The original contributions presented in the study are included in the article/Supplementary material, further inquiries can be directed to the corresponding author.
