# Supplementary material for: Dynamic trend analysis of global psoriasis burden from 1990 to 2021: a study of gender, age, and regional differences based on GBD 2021 data
Source: Front Public Health. 2025 Jul 9;13:1518681. doi: 10.3389/fpubh.2025.1518681 (PMC12283580; doi:10.3389/fpubh.2025.1518681)

Supplementary table 1. Age-standardized incidence rate and AAPC of psoriasis globally and by region from 1990 to 2021.

location	1990		2021		AAPC (95%CI)
	Number (95%UI)	ASR (95%UI)	Number (95%UI)	ASR (95%UI)	
Global	2852675.8 (2764294.4 to 2943367.2)	57.0 (55.3 to 58.8)	5099418.3 (4945748.1 to 5254031.0)	62.0 (60.1 to 63.9)	0.27 (0.26 to 0.28)
High SDI	777403.0 (754267.8 to 801779.7)	83.7 (81.2 to 86.3)	1134087.5 (1099752.4 to 1167976.4)	92.3 (89.6 to 94.9)	0.3 (0.28 to 0.31)
High-middle SDI	606238.5 (587340.7 to 625371.2)	56.5 (54.8 to 58.3)	1008069.6 (975952.4 to 1040169.5)	66.9 (64.8 to 69.0)	0.54 (0.54 to 0.55)
Middle SDI	843818.8 (816665.9 to 871346.7)	53.7 (52.1 to 55.3)	1641222.1 (1590306.0 to 1691699.4)	62.7 (60.8 to 64.7)	0.5 (0.49 to 0.5)
Low-middle SDI	475261.2 (459036.1 to 489690.4)	45.9 (44.4 to 47.3)	947741.1 (916034.2 to 979472.8)	50.7 (49.0 to 52.3)	0.31 (0.29 to 0.32)
Low SDI	146976.2 (141645.3 to 151520.3)	34.4 (33.3 to 35.6)	363918.8 (350683.3 to 375676.6)	37.2 (35.9 to 38.4)	0.24 (0.22 to 0.25)
High-income Asia Pacific	98706.2 (95548.7 to 101802.6)	52.7 (51.0 to 54.3)	126176.7 (121983.0 to 130129.5)	56.7 (55.0 to 58.5)	0.22 (0.2 to 0.23)
High-income North America	279448.7 (271343.3 to 288121.8)	96.5 (93.6 to 99.4)	422371.3 (410310.8 to 434102.1)	103.5 (100.8 to 106.1)	0.23 (0.21 to 0.24)
Western Europe	430713.0 (417260.5 to 444548.1)	103.7 (100.4 to 107.0)	565866.3 (547560.0 to 584609.2)	115.3 (111.8 to 118.9)	0.32 (0.3 to 0.33)
Australasia	14069.9 (13562.3 to 14613.9)	66.6 (64.1 to 69.1)	26079.6 (25106.4 to 27050.0)	75.9 (73.2 to 78.7)	0.41 (0.39 to 0.42)
Andean Latin America	31948.6 (30728.0 to 33078.5)	92.3 (88.8 to 95.5)	69315.2 (66699.9 to 71992.7)	105.0 (101.1 to 109.1)	0.42 (0.41 to 0.42)
Tropical Latin America	81446.9 (78601.2 to	57.5 (55.7 to	138658.9 (134234.8 to	57.8 (55.9 to	0.02 (0.01 to

	83874.8)	59.3)	143056.3)	59.5)	0.02)
Central Latin America	106949.4 (102905.0 to 110561.0)	72.8 (70.4 to 75.3)	203443.3 (196311.4 to 210198.6)	78.6 (75.9 to 81.2)	0.25 (0.25 to 0.26)
Southern Latin America	33084.1 (31783.9 to 34406.3)	67.7 (65.1 to 70.4)	55542.8 (53414.8 to 57531.3)	76.9 (73.9 to 79.7)	0.39 (0.37 to 0.41)
Caribbean	24182.0 (23237.2 to 25081.9)	72.9 (70.0 to 75.5)	37556.8 (36151.9 to 38986.4)	76.4 (73.5 to 79.2)	0.16 (0.15 to 0.16)
Central Europe	73853.9 (71481.4 to 76385.4)	55.2 (53.4 to 57.0)	81011.8 (78559.2 to 83476.2)	61.5 (59.8 to 63.3)	0.35 (0.35 to 0.36)
Eastern Europe	128895.5 (124591.3 to 133152.6)	52.9 (51.2 to 54.7)	139137.5 (134768.5 to 143725.4)	58.6 (56.8 to 60.6)	0.34 (0.33 to 0.34)
Central Asia	32184.2 (31013.2 to 33420.1)	50.7 (48.9 to 52.6)	54029.3 (52019.6 to 56016.5)	55.7 (53.6 to 57.8)	0.31 (0.3 to 0.31)
North Africa and Middle East	152645.4 (147189.4 to 157649.6)	52.7 (50.9 to 54.4)	395336.5 (381884.7 to 409179.7)	64.0 (61.8 to 66.1)	0.63 (0.63 to 0.63)
South Asia	453226.3 (438163.6 to 467391.2)	45.4 (44.0 to 46.8)	910977.4 (881351.7 to 941591.2)	49.4 (47.8 to 51.0)	0.25 (0.22 to 0.28)
Southeast Asia	236373.2 (228543.3 to 244458.6)	58.0 (56.2 to 59.9)	491723.9 (475357.4 to 507614.6)	67.7 (65.5 to 69.8)	0.5 (0.5 to 0.5)
East Asia	543655.0 (526680.4 to 562339.1)	47.0 (45.5 to 48.5)	1037234.5 (1003142.1 to 1069796.6)	59.1 (57.2 to 60.9)	0.74 (0.74 to 0.75)
Oceania	2570.5 (2468.2 to 2666.8)	47.5 (45.6 to 49.3)	6516.1 (6251.8 to 6776.2)	52.8 (50.6 to 54.9)	0.34 (0.34 to 0.35)
Western Sub-Saharan Africa	62700.0 (60472.5 to 64604.8)	37.5 (36.3 to 38.7)	181649.8 (175025.0 to 187328.1)	42.2 (40.8 to 43.5)	0.38 (0.37 to 0.38)
Eastern Sub-Saharan Africa	34537.0 (33240.0 to 35702.1)	22.2 (21.4 to 22.9)	82617.8 (79425.4 to 85440.9)	22.9 (22.1 to 23.6)	0.1 (0.09 to 0.1)
Central Sub-Saharan Africa	16616.2 (15928.6 to 17255.6)	35.1 (33.8 to 36.4)	47617.0 (45582.5 to 49512.9)	39.3 (37.7 to 40.8)	0.36 (0.36 to 0.37)

Southern Sub-Saharan Africa	14869.8 (14319.5 to 15350.3)	31.8 (30.7 to 32.9)	26555.9 (25626.7 to 27433.8)	33.8 (32.6 to 34.9)	0.19 (0.18 to 0.19)

Abbreviations: ASR: age-standardized rate; AAPC: average annual percentage change; CI: confidence interval; UI: uncertainty interval.

Supplementary table 2. Age-standardized DALY rate and AAPC of psoriasis globally and by region from 1990 to 2021.

location	1990		2021		AAPC (95%UI)
	Numbe (95%UI)	ASR (95%UI)	Number (95%UI)	ASR (95%UI)	
Global	1996756.1 (1441303.4 to 2670809.4)	41.1 (29.8 to 54.9)	3689928.3 (2684039.9 to 4917112.8)	44.4 (32.2 to 59.2)	0.24 (0.23 to 0.25)
High SDI	658614.7 (477507.6 to 881958.9)	68.3 (49.4 to 91.4)	975397.6 (714215.4 to 1297563.3)	73.3 (53.2 to 97.7)	0.22 (0.22 to 0.23)
High-middle SDI	418699.1 (302033.7 to 561332.7)	39.1 (28.2 to 52.3)	750440.6 (546290.2 to 999445.8)	47.5 (34.5 to 63.3)	0.64 (0.63 to 0.64)
Middle SDI	546044.2 (393203.0 to 729744.9)	35.9 (25.9 to 47.9)	1144223.0 (829568.3 to 1527293.8)	43.2 (31.3 to 57.6)	0.59 (0.58 to 0.6)
Low-middle SDI	286019.8 (206328.3 to 382896.9)	28.9 (20.8 to 38.7)	599948.1 (432683.6 to 806757.5)	32.5 (23.5 to 43.5)	0.36 (0.33 to 0.38)
Low SDI	85317.3 (61709.2 to 115148.3)	21.2 (15.2 to 28.3)	216728.4 (157310.2 to 292188.1)	23.1 (16.7 to 30.9)	0.27 (0.25 to 0.29)
High-income Asia Pacific	62535.7 (45549.2 to 83684.6)	32.7 (23.7 to 43.8)	82202.2 (59692.8 to 109283.8)	34.6 (24.9 to 46.2)	0.18 (0.17 to 0.18)
High-income North America	241412.9 (175174.6 to 323056.3)	80.3 (58.0 to 107.4)	362213.0 (265351.1 to 476605.0)	83.1 (60.4 to 109.7)	0.11 (0.11 to 0.12)
Western Europe	396707.9 (286401.9 to 531455.8)	90.0 (64.8 to 120.1)	541494.7 (393879.2 to 723857.4)	99.8 (72.2 to 133.9)	0.33 (0.32 to 0.34)
Australasia	9772.6 (7054.5 to 13011.0)	45.3 (32.6 to 60.6)	18758.0 (13429.6 to 24929.5)	52.1 (37.3 to 69.6)	0.45 (0.44 to 0.47)

Andean Latin America	24351.0 (17724.5 to 32998.1)	74.5 (54.0 to 100.6)	58153.3 (42211.0 to 77635.5)	88.4 (64.1 to 117.7)	0.55 (0.54 to 0.56)
Tropical Latin America	49228.2 (35412.3 to 65765.8)	35.5 (25.6 to 47.6)	85291.0 (61916.9 to 113230.1)	35.2 (25.5 to 46.8)	-0.04 (-0.04 to -0.03)
Central Latin America	70759.0 (50797.8 to 94585.9)	50.5 (36.4 to 66.8)	144890.6 (104615.1 to 193604.5)	55.6 (40.1 to 74.3)	0.31 (0.31 to 0.31)
Southern Latin America	22484.8 (16294.0 to 29962.2)	46.3 (33.6 to 61.7)	39041.0 (28403.3 to 52060.3)	52.6 (38.3 to 70.5)	0.41 (0.4 to 0.42)
Caribbean	16638.7 (11972.6 to 22187.9)	51.2 (36.9 to 68.5)	26796.2 (19201.3 to 35719.2)	53.7 (38.4 to 71.8)	0.16 (0.15 to 0.16)
Central Europe	48924.4 (35515.3 to 64848.2)	35.9 (26.0 to 47.5)	58899.3 (43017.5 to 78086.6)	41.4 (30.0 to 55.2)	0.47 (0.47 to 0.48)
Eastern Europe	83051.4 (60340.4 to 110730.2)	33.4 (24.3 to 44.5)	95182.9 (69625.2 to 127100.0)	37.9 (27.5 to 50.5)	0.42 (0.41 to 0.43)
Central Asia	19573.7 (14084.9 to 26020.4)	32.0 (23.0 to 42.5)	34360.4 (24873.9 to 45831.3)	35.8 (25.9 to 47.8)	0.37 (0.36 to 0.37)
North Africa and Middle East	94032.9 (67582.8 to 126814.1)	34.5 (25.0 to 46.1)	265386.0 (189416.7 to 355031.4)	43.6 (31.2 to 58.3)	0.76 (0.76 to 0.76)
South Asia	272799.4 (197005.1 to 366604.5)	28.5 (20.6 to 38.2)	570539.0 (410766.2 to 765530.3)	31.0 (22.4 to 41.7)	0.24 (0.18 to 0.29)
Southeast Asia	152518.6 (109885.9 to 204778.4)	39.0 (28.2 to 52.2)	342924.9 (249665.4 to 459152.2)	47.2 (34.3 to 63.2)	0.62 (0.61 to 0.62)
East Asia	351134.0 (252981.4 to 471594.2)	31.0 (22.4 to 41.5)	742940.3 (539071.3 to 990457.2)	40.6 (29.4 to 54.3)	0.88 (0.87 to 0.89)
Oceania	1501.0 (1074.9 to 1979.9)	29.2 (21.0 to 38.4)	3902.5 (2786.3 to 5189.6)	32.9 (23.6 to 43.6)	0.39 (0.38 to 0.4)
Western Sub-Saharan Africa	41844.8 (30390.7 to 56502.7)	27.1 (19.7 to 36.3)	125843.7 (91563.3 to 169081.6)	31.6 (22.9 to 42.2)	0.48 (0.47 to 0.49)

Eastern Sub-Saharan Africa	18691.9 (13485.9 to 25188.5)	12.6 (9.1 to 16.9)	45262.2 (33034.6 to 60751.7)	13.0 (9.5 to 17.3)	0.1 (0.09 to 0.11)
Central Sub-Saharan Africa	9932.7 (7107.7 to 13278.3)	22.4 (16.1 to 29.6)	29712.0 (21555.2 to 40044.6)	26.0 (18.8 to 34.9)	0.47 (0.46 to 0.48)
Southern Sub-Saharan Africa	8860.8 (6395.6 to 11753.2)	19.8 (14.3 to 26.3)	16134.8 (11578.8 to 21561.3)	20.8 (14.9 to 27.8)	0.16 (0.15 to 0.16)

Abbreviations: ASR: age-standardized rate; AAPC: average annual percentage change; CI: confidence interval; UI: uncertainty interval.

Supplement table 3. Age-standardized prevalence rates and AAPC for psoriasis at the national level from 1990 to 2021.

location	1990		2021		AAPC (95%CI)
	Number (95%UI)	ASR (95%UI)	Number (95%UI)	ASR (95%UI)	
Afghanistan	26140.2 (25212.7 to 27104.6)	324.7 (312.8 to 336.9)	86155.5 (82837.1 to 89601.1)	375.6 (362.5 to 389.4)	0.47 (0.47 to 0.48)
Albania	11094.7 (10665.6 to 11488.8)	377.1 (363.0 to 390.5)	14315.5 (13800.7 to 14857.9)	442.6 (427.6 to 458.3)	0.52 (0.52 to 0.53)
Algeria	82210.8 (79042.2 to 84994.3)	418.5 (402.8 to 432.6)	221318.5 (212324.9 to 230299.6)	506.0 (486.6 to 525.9)	0.61 (0.61 to 0.61)
American Samoa	159.8 (153.6 to 165.9)	422.8 (406.1 to 438.8)	242.1 (232.7 to 251.5)	470.3 (452.9 to 487.9)	0.35 (0.34 to 0.35)
Andorra	724.9 (697.1 to 753.1)	1196.5 (1154.5 to 1240.6)	1448.2 (1395.3 to 1502.3)	1284.3 (1239.7 to 1330.8)	0.23 (0.22 to 0.23)
Angola	20275.9 (19492.3 to 21091.3)	249.4 (240.6 to 258.6)	80918.3 (77638.0 to 84305.6)	313.1 (301.2 to 324.8)	0.74 (0.73 to 0.74)

Antigua and Barbuda	360.0<br><br>(346.9 to 373.4)	632.9<br><br>(609.6 to 656.9)	705.2<br><br>(678.8 to 733.3)	703.2<br><br>(678.4 to 729.7)	0.34<br><br>(0.34 to 0.34)
Argentina	175417.7<br><br>(169173.0 to 182185.2)	537.2<br><br>(517.9 to 558.3)	298118.2<br><br>(287043.0 to 310271.2)	604.7<br><br>(581.9 to 629.2)	0.38<br><br>(0.38 to 0.39)
Armenia	11891.0<br><br>(11423.6 to 12394.5)	363.2<br><br>(349.1 to 377.6)	14941.3<br><br>(14349.4 to 15511.4)	427.6<br><br>(410.9 to 443.9)	0.53<br><br>(0.53 to 0.54)
Australia	95559.7<br><br>(92178.4 to 99243.1)	529.2<br><br>(510.7 to 549.6)	185586.9<br><br>(178847.8 to 192465.7)	610.2<br><br>(587.8 to 633.0)	0.47<br><br>(0.45 to 0.48)
Austria	98567.1<br><br>(95214.5 to 102454.0)	1093.9<br><br>(1056.1 to 1135.0)	137940.4<br><br>(133128.8 to 142796.3)	1213.0<br><br>(1170.1 to 1255.1)	0.33<br><br>(0.33 to 0.33)
Azerbaijan	25025.7<br><br>(23979.6 to 25988.9)	378.5<br><br>(364.0 to 392.8)	48973.1<br><br>(47054.7 to 50914.5)	426.3<br><br>(410.9 to 442.1)	0.39<br><br>(0.38 to 0.39)
Bahamas	1582.5<br><br>(1524.2 to 1642.8)	681.7<br><br>(656.5 to 707.8)	3042.3<br><br>(2928.9 to 3152.5)	720.9<br><br>(695.3 to 745.9)	0.19<br><br>(0.18 to 0.19)
Bahrain	2022.1<br><br>(1938.0 to 2103.3)	459.2<br><br>(441.3 to 476.3)	9126.8<br><br>(8715.2 to 9529.1)	551.1<br><br>(529.7 to 571.6)	0.59<br><br>(0.59 to 0.59)
Bangladesh	265584.6<br><br>(255657.8 to 275330.3)	303.0<br><br>(292.5 to 313.7)	576489.5<br><br>(557167.3 to 596919.7)	352.5<br><br>(341.1 to 364.4)	0.49<br><br>(0.49 to 0.49)
Barbados	1758.5<br><br>(1691.8 to 1823.8)	681.9<br><br>(655.7 to 706.8)	2564.6<br><br>(2462.6 to 2658.6)	707.6<br><br>(679.8 to 733.3)	0.12<br><br>(0.12 to 0.12)
Belarus	43116.7<br><br>(41425.4 to 44836.5)	375.1<br><br>(360.8 to 389.5)	50455.4<br><br>(48378.6 to 52342.8)	436.0<br><br>(418.4 to 452.2)	0.49<br><br>(0.48 to 0.49)

Belgium	125286.7<br><br>(121072.7 to 129595.6)	1074.0<br><br>(1037.7 to 1110.6)	171654.7<br><br>(165864.7 to 177958.4)	1206.3<br><br>(1166.2 to 1247.9)	0.38<br><br>(0.37 to 0.38)
Belize	823.9<br><br>(794.2 to 854.0)	548.2<br><br>(528.1 to 567.4)	2538.4<br><br>(2442.0 to 2637.1)	620.9<br><br>(598.4 to 643.7)	0.4<br><br>(0.4 to 0.41)
Benin	6962.1<br><br>(6695.2 to 7240.9)	188.3<br><br>(181.8 to 194.9)	21653.0<br><br>(20879.0 to 22458.3)	201.5<br><br>(194.9 to 208.8)	0.21<br><br>(0.2 to 0.22)
Bermuda	418.1<br><br>(402.1 to 434.1)	658.8<br><br>(634.3 to 683.1)	578.4<br><br>(556.3 to 600.2)	710.8<br><br>(684.4 to 737.5)	0.24<br><br>(0.24 to 0.25)
Bhutan	1474.1<br><br>(1415.7 to 1530.4)	286.5<br><br>(276.4 to 296.7)	2585.4<br><br>(2487.1 to 2682.4)	340.5<br><br>(328.5 to 352.8)	0.56<br><br>(0.56 to 0.56)
Bolivia (Plurinational State of)	41232.1<br><br>(39650.1 to 42826.3)	778.1<br><br>(750.5 to 807.1)	106476.4<br><br>(102462.8 to 110469.7)	936.5<br><br>(902.6 to 971.4)	0.6<br><br>(0.6 to 0.6)
Bosnia and Herzegovina	16918.0<br><br>(16314.7 to 17540.8)	360.4<br><br>(348.2 to 373.2)	18933.0<br><br>(18205.0 to 19690.0)	451.0<br><br>(435.7 to 466.7)	0.73<br><br>(0.72 to 0.74)
Botswana	2277.4<br><br>(2186.8 to 2368.0)	215.9<br><br>(207.8 to 224.1)	5653.5<br><br>(5430.7 to 5891.2)	247.2<br><br>(237.6 to 256.6)	0.43<br><br>(0.43 to 0.44)
Brazil	554870.9<br><br>(536838.1 to 572869.4)	415.9<br><br>(402.7 to 429.4)	971497.2<br><br>(940061.6 to 1003122.9)	410.7<br><br>(397.8 to 423.8)	-0.04<br><br>(-0.04 to -0.03)
Brunei Darussalam	834.4<br><br>(803.2 to 870.4)	387.4<br><br>(373.8 to 402.6)	1986.8<br><br>(1915.3 to 2068.7)	419.2<br><br>(404.9 to 435.2)	0.25<br><br>(0.25 to 0.26)
Bulgaria	41323.2<br><br>(39833.3 to	403.9<br><br>(389.8 to	41079.4<br><br>(39536.4 to	469.7<br><br>(453.1 to	0.49<br><br>(0.48 to

	42848.1)	418.5)	42739.2)	487.5)	0.49)
Burkina Faso	13004.4<br><br>(12506.9 to 13521.0)	175.1<br><br>(169.2 to 181.3)	34066.1<br><br>(32836.1 to 35345.8)	188.0<br><br>(181.4 to 195.1)	0.23<br><br>(0.22 to 0.24)
Burundi	6007.6<br><br>(5793.8 to 6230.7)	141.1<br><br>(136.0 to 145.7)	14023.4<br><br>(13536.6 to 14516.5)	134.2<br><br>(129.5 to 138.5)	-0.17<br><br>(-0.17 to -0.16)
Cabo Verde	564.7<br><br>(544.6 to 584.8)	195.7<br><br>(188.9 to 202.7)	1256.7<br><br>(1211.5 to 1304.0)	224.5<br><br>(216.8 to 232.3)	0.45<br><br>(0.43 to 0.46)
Cambodia	29315.4<br><br>(28254.5 to 30402.0)	384.2<br><br>(370.4 to 399.1)	74439.6<br><br>(71637.6 to 77362.6)	469.1<br><br>(451.7 to 487.3)	0.65<br><br>(0.65 to 0.66)
Cameroon	16893.4<br><br>(16301.3 to 17495.2)	205.8<br><br>(198.6 to 212.7)	58628.5<br><br>(56455.6 to 60883.6)	224.8<br><br>(217.0 to 232.8)	0.28<br><br>(0.27 to 0.3)
Canada	239630.8<br><br>(231549.9 to 247991.7)	810.7<br><br>(783.0 to 837.6)	415095.7<br><br>(400829.0 to 430450.7)	908.8<br><br>(877.5 to 941.0)	0.37<br><br>(0.37 to 0.37)
Central African Republic	5413.2<br><br>(5198.8 to 5625.7)	246.7<br><br>(237.2 to 256.3)	12354.3<br><br>(11861.9 to 12868.1)	270.8<br><br>(260.7 to 282.1)	0.3<br><br>(0.29 to 0.3)
Chad	8024.6<br><br>(7723.8 to 8303.0)	171.6<br><br>(165.3 to 177.4)	23871.3<br><br>(22926.2 to 24802.0)	179.5<br><br>(173.6 to 185.6)	0.14<br><br>(0.13 to 0.15)
Chile	66108.0<br><br>(63615.6 to 68621.5)	529.9<br><br>(509.8 to 548.9)	133238.2<br><br>(128183.8 to 138568.9)	622.8<br><br>(599.3 to 647.3)	0.52<br><br>(0.52 to 0.52)
China	3921863.4<br><br>(3789618.5 to 4053307.5)	362.0<br><br>(350.3 to 373.6)	8453044.9<br><br>(8161388.8 to 8743685.0)	474.0<br><br>(458.6 to 488.9)	0.88<br><br>(0.87 to 0.88)

Colombia	170757.8<br><br>(164024.9 to 177542.9)	606.4<br><br>(584.5 to 629.8)	352204.0<br><br>(339234.9 to 365238.9)	674.3<br><br>(649.7 to 699.4)	0.34<br><br>(0.34 to 0.35)
Comoros	525.8<br><br>(506.2 to 545.6)	146.0<br><br>(141.1 to 151.0)	1040.0<br><br>(1004.1 to 1076.0)	151.4<br><br>(146.3 to 156.4)	0.11<br><br>(0.1 to 0.12)
Congo	5732.5<br><br>(5503.6 to 5947.6)	294.9<br><br>(283.9 to 305.3)	17054.6<br><br>(16345.8 to 17723.6)	353.4<br><br>(339.4 to 366.8)	0.59<br><br>(0.58 to 0.59)
Cook Islands	67.8<br><br>(65.2 to 70.3)	412.2<br><br>(396.1 to 427.0)	100.4<br><br>(96.7 to 104.4)	481.9<br><br>(464.9 to 500.6)	0.51<br><br>(0.5 to 0.51)
Costa Rica	16400.9<br><br>(15705.0 to 17016.2)	624.6<br><br>(600.2 to 647.3)	35331.4<br><br>(34045.7 to 36547.8)	688.7<br><br>(663.8 to 712.1)	0.32<br><br>(0.31 to 0.32)
Coted'Ivoire	18517.4<br><br>(17764.9 to 19252.9)	196.1<br><br>(189.1 to 203.2)	49021.4<br><br>(47152.0 to 51012.9)	211.4<br><br>(204.0 to 218.9)	0.24<br><br>(0.23 to 0.25)
Croatia	23011.5<br><br>(22147.7 to 23907.6)	412.6<br><br>(397.1 to 427.8)	25712.8<br><br>(24794.7 to 26657.0)	478.0<br><br>(461.1 to 495.8)	0.48<br><br>(0.47 to 0.49)
Cuba	67429.0<br><br>(64958.9 to 70257.6)	619.2<br><br>(596.5 to 644.2)	88182.0<br><br>(84581.3 to 91819.8)	644.9<br><br>(620.6 to 668.5)	0.13<br><br>(0.13 to 0.14)
Cyprus	8601.2<br><br>(8305.5 to 8915.8)	1059.1<br><br>(1022.6 to 1095.6)	20648.3<br><br>(19923.1 to 21434.4)	1253.5<br><br>(1208.9 to 1297.5)	0.54<br><br>(0.54 to 0.55)
Czechia	47778.7<br><br>(46104.9 to 49543.0)	411.7<br><br>(397.3 to 426.9)	63896.3<br><br>(61524.5 to 66203.6)	478.8<br><br>(462.1 to 495.5)	0.49<br><br>(0.48 to 0.49)
Democratic People's Republic of Korea	45058.3<br><br>(43444.9 to 46780.5)	228.8<br><br>(220.9 to 236.9)	85034.6<br><br>(81743.5 to 88015.6)	276.2<br><br>(266.1 to 285.6)	0.61<br><br>(0.61 to 0.61)

Democratic Republic of the Congo	80108.6 (76837.4 to 83182.3)	266.4 (256.7 to 275.8)	219104.0 (210292.1 to 227525.7)	294.7 (283.4 to 305.2)	0.33 (0.32 to 0.33)
Denmark	66984.0 (64568.0 to 69367.3)	1112.0 (1071.4 to 1149.7)	88852.6 (85813.4 to 92103.9)	1222.9 (1178.2 to 1264.2)	0.31 (0.3 to 0.31)
Djibouti	469.1 (451.2 to 486.9)	143.7 (138.4 to 148.5)	1674.8 (1613.8 to 1733.8)	147.0 (142.0 to 151.8)	0.07 (0.06 to 0.08)
Dominica	411.2 (395.9 to 425.7)	622.6 (599.0 to 645.1)	522.2 (501.8 to 543.4)	698.7 (672.0 to 726.8)	0.37 (0.37 to 0.37)
Dominican Republic	32836.9 (31584.4 to 34082.4)	542.9 (523.1 to 563.9)	67023.3 (64495.7 to 69652.4)	611.5 (588.2 to 635.0)	0.39 (0.39 to 0.39)
Ecuador	75927.7 (73071.2 to 78927.8)	894.2 (861.2 to 931.1)	187642.1 (180411.8 to 194475.2)	1048.6 (1008.6 to 1086.8)	0.52 (0.51 to 0.52)
Egypt	106929.8 (103167.2 to 110596.0)	234.7 (226.7 to 242.6)	274834.5 (264648.9 to 284863.6)	286.9 (276.5 to 297.3)	0.65 (0.65 to 0.66)
El Salvador	23925.1 (23028.5 to 24852.3)	538.0 (519.7 to 556.8)	39017.0 (37589.4 to 40509.5)	615.1 (592.2 to 638.2)	0.43 (0.43 to 0.44)
Equatorial Guinea	860.1 (826.7 to 895.4)	255.2 (245.6 to 264.8)	4820.1 (4609.0 to 5031.5)	385.5 (371.4 to 401.0)	1.34 (1.33 to 1.35)
Eritrea	3556.0 (3427.3 to 3685.5)	136.8 (132.0 to 141.5)	7884.5 (7619.4 to 8153.9)	142.5 (137.9 to 147.1)	0.13 (0.12 to 0.14)
Estonia	6635.3 (6368.9 to 6907.7)	379.4 (365.1 to 394.3)	7278.9 (6985.5 to 7567.4)	451.3 (434.1 to 467.9)	0.57 (0.56 to 0.57)

Eswatini	1285.8 (1235.3 to 1339.7)	207.8 (199.8 to 215.8)	2357.5 (2263.2 to 2457.3)	232.5 (224.1 to 241.2)	0.36 (0.35 to 0.37)
Ethiopia	60888.9 (58868.0 to 62973.3)	158.8 (153.6 to 163.9)	144590.4 (139453.2 to 149621.2)	165.9 (160.3 to 171.2)	0.14 (0.14 to 0.14)
Fiji	2326.9 (2233.2 to 2418.7)	377.1 (362.9 to 392.0)	3990.9 (3842.8 to 4139.6)	440.6 (424.6 to 456.5)	0.51 (0.5 to 0.51)
Finland	60384.4 (58208.1 to 62710.0)	1042.5 (1006.7 to 1080.7)	81398.5 (78523.5 to 84408.9)	1159.3 (1121.5 to 1200.1)	0.34 (0.34 to 0.34)
France	693448.1 (667217.1 to 717479.0)	1068.0 (1028.7 to 1104.3)	990073.3 (956669.7 to 1026079.9)	1213.8 (1170.5 to 1256.1)	0.41 (0.4 to 0.42)
Gabon	2500.4 (2405.7 to 2597.0)	301.6 (290.4 to 313.2)	6193.4 (5950.5 to 6433.9)	378.2 (363.6 to 392.5)	0.73 (0.73 to 0.73)
Gambia	1439.1 (1383.2 to 1494.3)	189.6 (182.9 to 196.2)	4058.4 (3903.1 to 4214.3)	206.3 (199.4 to 213.5)	0.27 (0.26 to 0.28)
Georgia	23892.5 (22960.0 to 24748.6)	404.8 (389.1 to 419.1)	18394.4 (17645.6 to 19057.6)	432.0 (415.0 to 447.5)	0.21 (0.21 to 0.22)
Germany	1361441.3 (1315243.9 to 1411197.7)	1421.5 (1373.1 to 1470.8)	1766751.8 (1706471.7 to 1831074.0)	1593.7 (1543.4 to 1652.1)	0.36 (0.34 to 0.38)
Ghana	22990.6 (21635.6 to 24265.0)	191.5 (181.6 to 201.9)	63165.7 (59672.9 to 66650.8)	212.0 (201.1 to 222.9)	0.33 (0.32 to 0.34)

Greece	127790.8 (122824.4 to 132597.0)	1060.4 (1020.0 to 1098.7)	157385.4 (151646.2 to 162858.4)	1193.1 (1153.8 to 1236.6)	0.38 (0.38 to 0.38)
Greenland	397.5 (382.0 to 413.9)	745.2 (718.2 to 771.8)	539.6 (520.5 to 560.5)	849.6 (821.7 to 880.5)	0.42 (0.42 to 0.43)
Grenada	439.6 (423.9 to 456.1)	574.2 (552.6 to 596.3)	744.4 (717.5 to 771.7)	668.7 (644.4 to 692.1)	0.49 (0.49 to 0.5)
Guam	529.7 (508.8 to 550.1)	446.2 (429.6 to 462.6)	909.3 (875.4 to 943.0)	507.8 (488.9 to 526.2)	0.43 (0.42 to 0.43)
Guatemala	32439.5 (31186.4 to 33617.8)	497.0 (478.6 to 516.0)	84973.3 (81719.8 to 88099.5)	581.0 (559.4 to 601.5)	0.51 (0.5 to 0.51)
Guinea	8924.6 (8606.0 to 9247.6)	183.1 (176.5 to 189.7)	21272.1 (20459.6 to 22103.0)	197.0 (189.8 to 203.5)	0.23 (0.21 to 0.24)
Guinea-Bissau	1462.3 (1411.9 to 1513.6)	186.9 (180.6 to 193.1)	3340.6 (3215.2 to 3474.0)	200.6 (193.8 to 207.4)	0.22 (0.21 to 0.23)
Guyana	3807.3 (3657.8 to 3946.1)	583.0 (561.5 to 602.5)	5006.1 (4814.9 to 5210.7)	663.4 (638.0 to 689.5)	0.42 (0.42 to 0.42)
Haiti	25657.2 (24692.4 to 26663.0)	485.4 (467.7 to 503.8)	60795.0 (58335.9 to 63155.3)	524.7 (505.7 to 544.6)	0.25 (0.25 to 0.26)
Honduras	18794.1 (18059.6 to 19552.9)	507.8 (488.6 to 527.0)	52968.7 (50877.3 to 55009.0)	575.0 (554.2 to 595.6)	0.4 (0.4 to 0.4)
Hungary	48571.5 (46548.3 to 50284.7)	404.2 (388.0 to 418.4)	57719.2 (55383.0 to 60003.4)	474.0 (456.6 to 492.1)	0.52 (0.51 to 0.52)

Iceland	2757.5 (2661.7 to 2859.9)	1052.4 (1016.8 to 1091.4)	4804.1 (4634.2 to 4987.1)	1165.9 (1125.7 to 1209.2)	0.33 (0.33 to 0.33)
India	2564832.2 (2483644.2 to 2651113.2)	342.0 (331.1 to 352.7)	5216088.7 (5050769.2 to 5385384.4)	365.8 (354.3 to 377.3)	0.17 (0.1 to 0.23)
Indonesia	706922.1 (683317.1 to 729988.7)	460.2 (445.0 to 475.1)	1613492.7 (1559130.4 to 1667709.2)	560.6 (542.2 to 578.2)	0.64 (0.63 to 0.65)
Iran (Islamic Republic of)	174295.2 (168850.5 to 179959.7)	397.3 (384.6 to 410.6)	463717.1 (447608.5 to 479919.3)	505.3 (488.9 to 522.3)	0.78 (0.77 to 0.78)
Iraq	68271.8 (65838.3 to 70825.8)	503.8 (486.0 to 522.7)	244030.2 (234191.9 to 253649.7)	655.7 (629.6 to 680.8)	0.85 (0.84 to 0.86)
Ireland	38753.9 (37347.5 to 40055.8)	1051.4 (1012.3 to 1088.7)	70635.9 (68064.4 to 73214.1)	1211.8 (1169.7 to 1256.3)	0.46 (0.46 to 0.46)
Israel	53430.2 (51675.3 to 55473.7)	1116.7 (1079.1 to 1160.3)	122817.2 (118826.0 to 127040.5)	1226.9 (1187.5 to 1269.4)	0.3 (0.3 to 0.3)
Italy	574001.9 (554343.2 to 595209.7)	853.0 (824.1 to 882.8)	716290.6 (691304.3 to 743396.5)	910.2 (878.6 to 943.4)	0.21 (0.19 to 0.21)
Jamaica	12703.1 (12220.4 to 13165.8)	601.9 (579.1 to 622.6)	19614.5 (18810.2 to 20317.4)	659.4 (632.4 to 682.5)	0.3 (0.29 to 0.3)
Japan	557779.8 (539465.4 to 576225.2)	382.8 (370.6 to 394.9)	660535.3 (638986.6 to 680859.2)	391.2 (379.0 to 403.4)	0.06 (0.06 to 0.07)

Jordan	11942.6 (11502.3 to 12388.7)	437.7 (421.8 to 455.0)	60547.7 (57878.2 to 63068.2)	521.7 (500.5 to 542.1)	0.57 (0.57 to 0.57)
Kazakhstan	58021.9 (55649.6 to 60095.2)	377.0 (361.8 to 390.4)	83228.9 (79781.2 to 86413.7)	429.3 (412.2 to 445.4)	0.42 (0.42 to 0.42)
Kenya	27057.4 (26163.7 to 28014.4)	156.6 (151.8 to 161.6)	68605.8 (66368.5 to 70928.1)	161.5 (156.3 to 166.5)	0.09 (0.09 to 0.1)
Kiribati	211.3 (203.8 to 219.3)	362.0 (349.4 to 375.9)	433.5 (416.8 to 449.5)	411.2 (396.6 to 426.5)	0.41 (0.41 to 0.42)
Kuwait	7409.4 (7097.1 to 7748.6)	494.7 (476.3 to 513.8)	30634.9 (29077.2 to 32057.6)	589.7 (566.5 to 611.6)	0.57 (0.57 to 0.57)
Kyrgyzstan	14145.5 (13591.7 to 14679.4)	368.1 (353.9 to 382.1)	25466.9 (24465.4 to 26470.2)	397.9 (382.3 to 413.6)	0.25 (0.25 to 0.26)
Lao People's Democratic Republic	12069.0 (11624.8 to 12535.6)	377.3 (363.9 to 392.9)	31123.0 (29953.5 to 32292.0)	470.8 (453.1 to 487.7)	0.72 (0.72 to 0.73)
Latvia	11559.8 (11109.4 to 12006.7)	385.8 (371.4 to 400.0)	10519.1 (10099.2 to 10926.1)	448.9 (432.6 to 465.7)	0.49 (0.48 to 0.49)
Lebanon	11119.2 (10704.2 to 11579.2)	413.7 (398.2 to 430.7)	30176.3 (29015.6 to 31379.5)	512.4 (493.7 to 532.0)	0.7 (0.69 to 0.7)
Lesotho	2467.8 (2370.5 to 2565.2)	194.1 (186.4 to 201.5)	3658.0 (3517.6 to 3805.9)	216.3 (208.5 to 224.8)	0.34 (0.33 to 0.35)
Liberia	3736.0 (3608.3 to 3878.9)	190.9 (184.3 to 197.6)	9382.0 (9016.3 to 9705.3)	203.8 (196.3 to 210.7)	0.22 (0.21 to 0.23)

Libya	13516.2 (13015.0 to 14014.7)	420.0 (404.3 to 435.4)	38161.8 (36531.6 to 39806.2)	524.8 (505.3 to 545.5)	0.72 (0.71 to 0.72)
Lithuania	15417.1 (14815.0 to 16023.8)	384.0 (369.0 to 398.8)	15985.8 (15333.9 to 16607.8)	464.8 (446.7 to 482.1)	0.62 (0.62 to 0.63)
Luxembourg	4988.6 (4815.7 to 5171.3)	1117.9 (1079.9 to 1158.0)	9825.0 (9460.6 to 10218.8)	1241.6 (1199.6 to 1287.4)	0.34 (0.34 to 0.34)
Madagascar	13028.4 (12566.7 to 13524.7)	140.6 (135.7 to 145.3)	32226.1 (30921.7 to 33391.5)	138.0 (133.0 to 142.8)	-0.06 (-0.07 to -0.05)
Malawi	10349.3 (9974.6 to 10746.0)	138.1 (133.6 to 143.0)	21893.6 (21058.3 to 22715.5)	140.5 (135.6 to 145.5)	0.05 (0.04 to 0.06)
Malaysia	73807.6 (70957.3 to 76675.1)	511.0 (491.3 to 531.3)	196569.1 (189067.3 to 204024.1)	609.9 (586.9 to 632.1)	0.58 (0.57 to 0.58)
Maldives	671.5 (644.8 to 697.4)	420.0 (404.1 to 436.4)	2914.9 (2791.8 to 3046.9)	556.8 (535.0 to 579.2)	0.92 (0.91 to 0.92)
Mali	12722.8 (12006.5 to 13412.8)	186.1 (176.1 to 196.2)	37543.5 (35470.9 to 39655.7)	202.3 (191.6 to 213.2)	0.27 (0.26 to 0.28)
Malta	4252.3 (4103.9 to 4404.3)	1056.7 (1020.8 to 1094.4)	6888.1 (6649.4 to 7156.0)	1211.8 (1171.5 to 1256.9)	0.44 (0.44 to 0.44)
Marshall Islands	113.0 (108.6 to 117.6)	357.2 (343.8 to 371.1)	211.0 (203.0 to 219.1)	410.2 (395.1 to 425.7)	0.45 (0.44 to 0.45)
Mauritania	3280.1 (3164.2 to 3404.1)	198.4 (191.5 to 205.4)	7987.9 (7689.6 to 8290.8)	217.6 (209.6 to 225.2)	0.3 (0.28 to 0.31)

Mauritius	4755.2<br><br>(4571.7 to 4941.7)	481.8<br><br>(464.2 to 499.9)	8794.9<br><br>(8444.8 to 9114.5)	568.8<br><br>(547.4 to 589.6)	0.54<br><br>(0.53 to 0.54)
Mexico	417870.1<br><br>(404273.8 to 431896.5)	583.7<br><br>(565.0 to 603.1)	860983.0<br><br>(832430.0 to 888521.9)	643.3<br><br>(622.5 to 663.3)	0.31<br><br>(0.31 to 0.32)
Micronesia (Federated States of)	286.0<br><br>(275.9 to 296.9)	369.7<br><br>(356.6 to 383.8)	409.6<br><br>(394.5 to 426.1)	425.3<br><br>(409.5 to 441.1)	0.45<br><br>(0.45 to 0.46)
Monaco	479.0<br><br>(463.2 to 496.4)	1196.1<br><br>(1152.6 to 1237.0)	655.6<br><br>(631.2 to 680.5)	1288.5<br><br>(1244.2 to 1336.3)	0.24<br><br>(0.23 to 0.24)
Mongolia	5700.7<br><br>(5472.2 to 5938.3)	333.8<br><br>(321.4 to 346.8)	12281.2<br><br>(11830.2 to 12750.4)	387.7<br><br>(374.4 to 402.3)	0.49<br><br>(0.48 to 0.49)
Montenegro	2717.5<br><br>(2622.2 to 2818.2)	419.6<br><br>(405.2 to 434.4)	3559.4<br><br>(3430.7 to 3692.0)	476.2<br><br>(459.9 to 494.1)	0.42<br><br>(0.41 to 0.42)
Morocco	79509.3<br><br>(76600.5 to 82558.0)	380.0<br><br>(365.2 to 394.3)	175290.0<br><br>(168440.6 to 182547.8)	459.0<br><br>(441.5 to 477.2)	0.61<br><br>(0.61 to 0.61)
Mozambique	13575.8<br><br>(13097.6 to 14093.6)	128.4<br><br>(124.1 to 132.7)	31889.0<br><br>(30781.3 to 33072.9)	132.5<br><br>(128.4 to 136.9)	0.1<br><br>(0.09 to 0.11)
Myanmar	127950.0<br><br>(123099.4 to 132773.3)	380.2<br><br>(366.0 to 394.8)	265165.5<br><br>(254407.5 to 275934.4)	475.3<br><br>(456.0 to 493.2)	0.73<br><br>(0.72 to 0.73)
Namibia	2516.6<br><br>(2412.5 to 2616.4)	220.5<br><br>(212.0 to 228.7)	5311.1<br><br>(5096.7 to 5532.6)	241.4<br><br>(232.1 to 250.5)	0.29<br><br>(0.28 to 0.3)
Nauru	31.5<br><br>(30.3 to 32.8)	399.1<br><br>(384.9 to 415.1)	41.5<br><br>(39.9 to 43.1)	452.5<br><br>(435.9 to 470.7)	0.41<br><br>(0.4 to 0.41)

Nepal	42570.6 (41088.0 to 44083.1)	262.5 (253.3 to 271.9)	101456.8 (97750.5 to 105363.5)	339.9 (327.9 to 352.4)	0.82 (0.8 to 0.84)
Netherlands	188619.9 (182523.7 to 195206.9)	1117.4 (1082.4 to 1156.1)	267514.0 (257150.7 to 277772.2)	1231.9 (1186.7 to 1280.5)	0.31 (0.31 to 0.32)
New Zealand	18551.9 (17915.9 to 19156.2)	515.4 (497.2 to 531.8)	34968.9 (33748.8 to 36098.2)	581.2 (561.1 to 602.0)	0.39 (0.38 to 0.39)
Nicaragua	15532.2 (14885.3 to 16179.6)	514.8 (495.4 to 534.7)	37240.2 (35799.4 to 38665.1)	586.4 (564.6 to 608.6)	0.42 (0.42 to 0.43)
Niger	10015.8 (9635.5 to 10398.9)	165.2 (159.5 to 170.8)	31127.5 (29970.9 to 32256.4)	166.2 (160.7 to 171.4)	0.02 (0.01 to 0.03)
Nigeria	328727.5 (317592.3 to 339953.5)	453.1 (438.6 to 467.9)	1010163.5 (975217.6 to 1044865.1)	551.1 (533.7 to 569.5)	0.63 (0.62 to 0.64)
Niue	8.4 (8.1 to 8.7)	389.2 (374.4 to 404.1)	8.7 (8.4 to 9.0)	459.2 (441.5 to 476.3)	0.54 (0.53 to 0.54)
North Macedonia	8120.5 (7820.2 to 8404.6)	397.2 (382.9 to 410.7)	12536.2 (12070.8 to 12989.8)	464.6 (448.4 to 480.6)	0.51 (0.5 to 0.51)
Northern Mariana Islands	179.0 (171.6 to 186.6)	462.8 (446.2 to 480.1)	272.5 (261.6 to 283.3)	496.8 (477.7 to 514.4)	0.23 (0.21 to 0.24)
Norway	37935.5 (36749.0 to 39181.3)	784.3 (759.0 to 810.8)	54113.5 (52358.6 to 56016.4)	825.8 (799.7 to 854.5)	0.17 (0.16 to 0.17)
Oman	6526.6 (6263.5 to	423.0 (407.0 to	26351.6 (25162.4 to	569.0 (547.4 to	0.96 (0.96 to

	6777.7)	438.8)	27590.1)	590.7)	0.96)
Pakistan	268780.2 (259688.0 to 277905.3)	294.1 (284.3 to 303.8)	706459.5 (681703.4 to 732163.7)	336.4 (325.4 to 347.8)	0.44 (0.43 to 0.44)
Palau	58.3 (56.0 to 60.9)	431.2 (414.6 to 448.7)	109.2 (104.5 to 113.7)	490.8 (471.4 to 509.8)	0.42 (0.41 to 0.43)
Palestine	5382.2 (5187.0 to 5581.2)	366.7 (353.5 to 380.1)	20251.9 (19453.3 to 21049.7)	462.4 (444.5 to 481.6)	0.75 (0.75 to 0.75)
Panama	13386.5 (12886.4 to 13913.2)	632.0 (608.7 to 655.0)	30400.2 (29216.9 to 31471.9)	695.8 (668.8 to 720.1)	0.31 (0.31 to 0.31)
Papua New Guinea	10158.0 (9737.9 to 10545.2)	322.9 (309.6 to 334.9)	31615.1 (30414.8 to 32821.6)	368.3 (354.7 to 382.6)	0.43 (0.42 to 0.43)
Paraguay	13717.7 (13242.4 to 14198.0)	402.2 (388.0 to 416.0)	28925.6 (27963.1 to 30042.0)	413.8 (399.9 to 428.9)	0.09 (0.09 to 0.09)
Peru	160362.4 (154310.9 to 166194.4)	864.9 (832.8 to 895.8)	374462.6 (360744.9 to 388837.4)	1029.5 (992.9 to 1067.9)	0.56 (0.55 to 0.57)
Philippines	231767.9 (223824.7 to 239191.1)	469.1 (453.6 to 483.8)	562034.6 (543414.4 to 580355.3)	532.3 (515.0 to 549.0)	0.41 (0.41 to 0.42)
Poland	186183.7 (179873.2 to 192446.2)	455.8 (440.8 to 470.5)	243479.8 (239071.0 to 247324.6)	509.1 (500.3 to 517.1)	0.36 (0.35 to 0.37)
Portugal	113670.0 (109844.4 to 117874.1)	993.2 (959.5 to 1029.1)	158342.6 (152596.2 to 164258.1)	1142.4 (1101.0 to 1184.5)	0.45 (0.45 to 0.45)

Puerto Rico	24660.4<br><br>(23683.1 to 25602.2)	678.4<br><br>(652.4 to 704.3)	30771.4<br><br>(29547.7 to 31887.7)	752.3<br><br>(724.7 to 778.2)	0.34<br><br>(0.33 to 0.34)
Qatar	1939.4<br><br>(1842.6 to 2034.7)	476.9<br><br>(459.3 to 494.7)	19124.5<br><br>(18115.1 to 20079.1)	592.5<br><br>(569.7 to 616.1)	0.7<br><br>(0.7 to 0.71)
Republic of Korea	149821.0<br><br>(144007.4 to 155518.9)	350.4<br><br>(337.5 to 363.0)	268360.3<br><br>(258316.6 to 278479.6)	399.6<br><br>(385.0 to 414.0)	0.42<br><br>(0.42 to 0.43)
Republic of Moldova	16619.5<br><br>(15999.1 to 17282.4)	364.9<br><br>(351.0 to 379.2)	18871.7<br><br>(18037.0 to 19600.5)	421.3<br><br>(403.9 to 437.7)	0.47<br><br>(0.46 to 0.47)
Romania	99980.8<br><br>(96133.2 to 103910.8)	389.3<br><br>(374.8 to 404.9)	104457.1<br><br>(100751.0 to 108521.7)	440.1<br><br>(424.6 to 455.8)	0.4<br><br>(0.39 to 0.4)
Russian Federation	645876.1<br><br>(624145.5 to 667266.5)	389.6<br><br>(376.0 to 401.9)	788018.4<br><br>(760582.9 to 814991.1)	445.6<br><br>(430.8 to 459.6)	0.44<br><br>(0.43 to 0.44)
Rwanda	6169.9<br><br>(5891.0 to 6440.8)	112.8<br><br>(108.1 to 117.9)	13190.0<br><br>(12591.2 to 13809.9)	117.0<br><br>(111.3 to 122.1)	0.11<br><br>(0.09 to 0.13)
Saint Kitts and Nevis	241.8<br><br>(233.1 to 251.1)	632.1<br><br>(608.7 to 656.4)	477.4<br><br>(456.6 to 496.8)	706.9<br><br>(678.6 to 733.5)	0.36<br><br>(0.36 to 0.36)
Saint Lucia	690.4<br><br>(665.3 to 718.0)	583.7<br><br>(562.4 to 608.0)	1356.3<br><br>(1302.4 to 1413.5)	661.4<br><br>(636.6 to 687.5)	0.4<br><br>(0.4 to 0.41)
Saint Vincent and the Grenadines	543.8<br><br>(522.4 to 563.5)	570.8<br><br>(549.9 to 592.6)	805.3<br><br>(774.2 to 837.5)	638.4<br><br>(615.0 to 663.0)	0.36<br><br>(0.36 to 0.36)
Samoa	497.1<br><br>(479.1 to 515.7)	386.1<br><br>(371.8 to 400.2)	805.6<br><br>(776.6 to 835.6)	438.2<br><br>(422.5 to 454.7)	0.41<br><br>(0.41 to 0.41)

San Marino	321.1 (310.1 to 333.5)	1169.1 (1128.5 to 1213.7)	535.8 (515.6 to 555.6)	1257.8 (1213.3 to 1301.0)	0.23 (0.23 to 0.24)
Sao Tome and Principe	201.9 (194.6 to 209.0)	207.5 (200.3 to 214.4)	444.5 (429.2 to 460.3)	230.5 (222.9 to 238.4)	0.34 (0.33 to 0.35)
Saudi Arabia	53963.9 (51800.8 to 56060.8)	440.7 (423.7 to 457.4)	231869.3 (221495.4 to 242682.3)	583.0 (561.0 to 605.1)	0.91 (0.9 to 0.91)
Senegal	11227.5 (10816.1 to 11653.3)	189.0 (182.0 to 196.0)	27754.2 (26745.5 to 28877.8)	206.7 (199.8 to 213.9)	0.29 (0.28 to 0.3)
Serbia	43488.4 (41894.7 to 45215.1)	402.6 (387.9 to 418.3)	52585.9 (50731.5 to 54562.8)	472.5 (455.6 to 489.9)	0.52 (0.52 to 0.53)
Seychelles	339.8 (327.3 to 352.5)	524.1 (505.0 to 544.2)	711.0 (681.2 to 739.4)	605.8 (582.7 to 628.9)	0.47 (0.47 to 0.48)
Sierra Leone	6364.7 (6142.7 to 6601.9)	189.4 (183.0 to 195.5)	14759.7 (14226.0 to 15326.8)	200.2 (193.6 to 206.9)	0.18 (0.17 to 0.19)
Singapore	12027.3 (11544.5 to 12491.0)	392.4 (378.3 to 406.4)	31631.1 (30397.8 to 32812.9)	442.0 (426.6 to 458.4)	0.39 (0.38 to 0.39)
Slovakia	22594.5 (21813.6 to 23391.3)	403.5 (389.7 to 417.7)	32001.3 (30815.3 to 33263.7)	473.4 (456.7 to 491.6)	0.52 (0.52 to 0.52)
Slovenia	9494.0 (9148.4 to 9858.0)	431.0 (415.4 to 447.1)	13054.8 (12504.0 to 13535.8)	494.6 (476.1 to 513.7)	0.45 (0.44 to 0.45)
Solomon Islands	772.8 (742.4 to 801.9)	315.0 (302.9 to 326.4)	2038.3 (1957.9 to 2114.7)	362.8 (349.5 to 375.9)	0.46 (0.45 to 0.46)

Somalia	7402.1<br><br>(7143.5 to 7679.9)	124.4<br><br>(120.6 to 128.4)	18560.9<br><br>(17876.8 to 19287.4)	114.9<br><br>(111.1 to 118.5)	-0.26<br><br>(-0.27 to -0.25)
South Africa	75792.2<br><br>(73153.7 to 78434.9)	235.9<br><br>(228.1 to 243.7)	142746.4<br><br>(138100.4 to 147794.0)	250.6<br><br>(242.6 to 259.2)	0.19<br><br>(0.18 to 0.2)
South Sudan	6444.9<br><br>(6213.8 to 6687.5)	140.0<br><br>(135.3 to 144.3)	10224.2<br><br>(9854.2 to 10605.4)	132.7<br><br>(128.4 to 137.2)	-0.18<br><br>(-0.18 to -0.17)
Spain	292922.6<br><br>(282611.1 to 303252.9)	672.3<br><br>(648.5 to 697.6)	426379.3<br><br>(415498.6 to 439628.3)	734.0<br><br>(715.2 to 756.4)	0.28<br><br>(0.27 to 0.29)
Sri Lanka	74870.0<br><br>(71851.3 to 77762.7)	492.5<br><br>(473.1 to 510.1)	145081.1<br><br>(139391.7 to 150999.2)	585.8<br><br>(563.8 to 609.5)	0.57<br><br>(0.56 to 0.58)
Sudan	54538.6<br><br>(52488.7 to 56445.6)	358.8<br><br>(345.3 to 372.2)	168075.0<br><br>(161916.0 to 174217.8)	470.7<br><br>(453.3 to 487.8)	0.88<br><br>(0.88 to 0.88)
Suriname	2152.6<br><br>(2071.1 to 2234.0)	616.1<br><br>(593.1 to 639.3)	4101.4<br><br>(3939.4 to 4251.3)	667.1<br><br>(641.5 to 691.2)	0.26<br><br>(0.26 to 0.26)
Sweden	81029.9<br><br>(78061.1 to 83816.5)	803.0<br><br>(775.3 to 830.4)	104274.7<br><br>(100752.4 to 108242.0)	830.2<br><br>(802.7 to 860.5)	0.1<br><br>(0.1 to 0.11)
Switzerland	96791.1<br><br>(93316.7 to 100238.1)	1204.3<br><br>(1163.6 to 1247.1)	146598.4<br><br>(140948.5 to 152012.4)	1299.1<br><br>(1250.3 to 1345.1)	0.24<br><br>(0.24 to 0.24)
Syrian Arab Republic	35356.6<br><br>(34074.0 to 36723.2)	380.4<br><br>(366.4 to 394.4)	70002.4<br><br>(67257.3 to 72612.6)	484.2<br><br>(465.7 to 501.5)	0.78<br><br>(0.78 to 0.79)
Taiwan (Province of China)	39633.9<br><br>(38489.2 to 41034.7)	202.7<br><br>(196.9 to 209.9)	81535.5<br><br>(79276.0 to 83930.9)	260.1<br><br>(253.2 to 267.4)	0.81<br><br>(0.79 to 0.82)

Tajikistan	14616.4 (14065.4 to 15225.2)	349.2 (336.0 to 362.4)	34254.1 (32884.0 to 35664.6)	380.0 (365.1 to 394.3)	0.27 (0.27 to 0.28)
Thailand	239706.1 (230514.5 to 248579.1)	469.9 (452.5 to 486.3)	476544.6 (458133.0 to 494877.5)	556.8 (536.2 to 577.9)	0.55 (0.55 to 0.56)
Timor-Leste	2261.3 (2169.7 to 2351.6)	392.1 (376.6 to 407.2)	5759.3 (5541.7 to 5967.8)	496.8 (478.0 to 514.0)	0.77 (0.77 to 0.78)
Togo	5496.8 (5281.6 to 5707.5)	197.3 (190.5 to 204.1)	15125.2 (14583.8 to 15687.8)	210.6 (203.2 to 217.6)	0.2 (0.19 to 0.22)
Tokelau	5.1 (4.9 to 5.3)	359.8 (345.3 to 373.2)	6.3 (6.0 to 6.5)	444.1 (427.3 to 460.7)	0.68 (0.68 to 0.69)
Tonga	286.7 (276.5 to 297.0)	369.1 (355.6 to 382.4)	400.7 (385.2 to 415.1)	427.8 (410.9 to 443.4)	0.48 (0.48 to 0.48)
Trinidad and Tobago	7369.1 (7087.8 to 7655.9)	669.0 (643.7 to 696.8)	11757.6 (11304.2 to 12222.6)	735.1 (708.0 to 762.2)	0.31 (0.31 to 0.31)
Tunisia	28626.9 (27597.3 to 29702.2)	404.8 (390.3 to 420.4)	65526.9 (63049.4 to 68234.3)	503.6 (484.9 to 523.1)	0.71 (0.7 to 0.71)
Turkey	267837.6 (257299.6 to 277913.7)	541.5 (520.8 to 561.8)	649200.1 (623744.4 to 674172.0)	704.9 (677.8 to 731.6)	0.86 (0.85 to 0.86)
Turkmenistan	11219.6 (10769.8 to 11678.8)	376.1 (361.8 to 390.5)	21379.8 (20570.2 to 22185.5)	424.9 (409.1 to 441.2)	0.4 (0.39 to 0.4)
Tuvalu	29.5 (28.4 to 30.7)	356.8 (343.4 to 370.4)	50.4 (48.4 to 52.3)	428.4 (412.2 to 444.3)	0.59 (0.59 to 0.6)

Uganda	17659.1 (16985.6 to 18305.1)	137.7 (133.3 to 142.3)	48894.7 (47229.9 to 50711.0)	147.8 (142.9 to 152.6)	0.23 (0.22 to 0.23)
Ukraine	234151.0 (225233.7 to 242721.8)	390.8 (376.5 to 404.5)	234953.9 (226407.2 to 243277.6)	431.1 (415.6 to 446.5)	0.32 (0.31 to 0.32)
United Arab Emirates	7924.7 (7563.2 to 8305.6)	483.9 (467.2 to 502.4)	71137.1 (67097.7 to 75081.7)	606.6 (583.5 to 629.9)	0.73 (0.72 to 0.73)
United Kingdom	596932.3 (578999.1 to 615093.7)	908.8 (880.6 to 936.7)	867722.5 (839921.6 to 895505.4)	1046.7 (1013.0 to 1080.3)	0.45 (0.44 to 0.46)
United Republic of Tanzania	29914.3 (28186.2 to 31579.8)	150.9 (142.6 to 158.9)	73730.6 (69783.5 to 77826.0)	155.4 (147.4 to 164.3)	0.09 (0.08 to 0.1)
United States of America	2579771.3 (2503078.4 to 2662180.6)	946.5 (918.7 to 975.1)	3906305.1 (3827070.9 to 3984120.1)	983.5 (965.1 to 1003.3)	0.13 (0.12 to 0.14)
United States Virgin Islands	715.6 (688.9 to 742.5)	688.9 (664.1 to 714.9)	817.4 (785.0 to 849.8)	748.0 (720.1 to 775.8)	0.26 (0.26 to 0.27)
Uruguay	17797.4 (17130.1 to 18436.8)	537.2 (517.0 to 556.8)	23346.6 (22481.8 to 24205.3)	597.9 (575.6 to 620.1)	0.35 (0.35 to 0.35)
Uzbekistan	59452.0 (57158.3 to 61927.6)	351.9 (338.9 to 365.9)	136910.3 (131440.0 to 142234.0)	409.0 (392.8 to 424.0)	0.49 (0.48 to 0.49)
Vanuatu	369.8 (355.3 to 384.5)	330.6 (317.9 to 342.8)	988.5 (951.7 to 1026.5)	376.5 (362.5 to 391.5)	0.42 (0.42 to 0.43)
Venezuela (Bolivarian Republic of)	98767.6 (94861.5 to	617.4 (594.8 to	185493.5 (178714.8 to	650.1 (627.1 to	0.17 (0.16 to

	102702.7)	640.3)	192739.1)	674.5)	0.17)
Viet Nam	234511.2<br><br>(225653.7 to 243683.1)	425.9<br><br>(410.7 to 442.5)	561516.8<br><br>(537641.0 to 585401.2)	529.8<br><br>(508.2 to 551.4)	0.71<br><br>(0.71 to 0.72)
Yemen	32673.4<br><br>(31379.5 to 33936.0)	345.3<br><br>(332.6 to 358.2)	118283.6<br><br>(113679.7 to 122847.2)	438.1<br><br>(421.8 to 454.7)	0.77<br><br>(0.77 to 0.77)
Zambia	8736.1<br><br>(8406.9 to 9064.2)	146.9<br><br>(142.2 to 152.2)	23690.0<br><br>(22811.6 to 24591.5)	152.7<br><br>(147.3 to 157.7)	0.12<br><br>(0.11 to 0.13)
Zimbabwe	17222.8<br><br>(16540.3 to 17897.1)	213.5<br><br>(205.9 to 221.2)	28512.5<br><br>(27408.5 to 29768.1)	217.1<br><br>(209.2 to 225.3)	0.05<br><br>(0.04 to 0.06)

Abbreviations: ASR: age-standardized rate; AAPC: average annual percentage change; CI: confidence interval; UI: uncertainty interval.

Supplement table 4. Age-standardized incidence rates and AAPC for psoriasis at the national level from 1990 to 2021.

location	1990		2021		AAPC (95%CI)
	Number (95%UI)	ASR (95%UI)	Number (95%UI)	ASR (95%UI)	
Afghanistan	3945.9<br><br>(3800.4 to 4088.4)	46.5<br><br>(44.9 to 48.3)	13536.7<br><br>(12942.6 to 14056.9)	53.5<br><br>(51.5 to 55.4)	0.45<br><br>(0.45 to 0.46)
Albania	1591.4<br><br>(1540.0 to 1650.6)	51.8<br><br>(50.2 to 53.8)	1763.5<br><br>(1701.3 to 1831.8)	58.7<br><br>(56.6 to 60.8)	0.41<br><br>(0.4 to 0.41)
Algeria	11638.4<br><br>(11188.1 to 12090.0)	54.8<br><br>(52.9 to 56.8)	28604.8<br><br>(27430.9 to 29712.9)	64.7<br><br>(62.1 to 67.1)	0.54<br><br>(0.54 to 0.54)
American Samoa	22.4<br><br>(21.5 to 23.3)	55.3<br><br>(53.0 to	31.3<br><br>(30.1 to	61.0<br><br>(58.5 to	0.32<br><br>(0.32 to

		57.4)	32.6)	63.2)	0.32)
Andorra	66.4 (63.9 to 68.9)	113.7 (109.5 to 117.6)	126.3 (121.6 to 131.4)	123.9 (119.8 to 128.3)	0.27 (0.25 to 0.28)
Angola	2984.0 (2849.3 to 3109.9)	33.7 (32.4 to 35.1)	11389.9 (10859.5 to 11919.0)	40.2 (38.5 to 41.9)	0.56 (0.56 to 0.57)
Antigua and Barbuda	43.8 (42.1 to 45.6)	76.4 (73.5 to 79.7)	80.6 (77.4 to 84.1)	83.0 (79.8 to 86.5)	0.27 (0.27 to 0.27)
Argentina	22298.4 (21412.7 to 23211.6)	67.8 (65.1 to 70.7)	36660.2 (35215.1 to 38003.0)	76.5 (73.4 to 79.3)	0.37 (0.34 to 0.38)
Armenia	1680.8 (1615.6 to 1753.9)	50.2 (48.3 to 52.3)	1892.7 (1812.9 to 1966.9)	57.0 (54.8 to 59.2)	0.42 (0.41 to 0.42)
Australia	11750.1 (11304.0 to 12223.2)	66.8 (64.2 to 69.4)	21832.2 (20968.9 to 22704.0)	76.2 (73.4 to 79.1)	0.42 (0.4 to 0.43)
Austria	9016.5 (8712.0 to 9334.6)	107.3 (103.7 to 110.9)	12161.1 (11714.8 to 12616.0)	119.4 (115.2 to 123.4)	0.33 (0.31 to 0.34)
Azerbaijan	3543.8 (3400.5 to 3699.6)	51.7 (49.6 to 53.9)	6491.7 (6228.5 to 6784.0)	56.9 (54.7 to 59.2)	0.32 (0.31 to 0.32)
Bahamas	192.9 (184.8 to 200.6)	80.2 (77.1 to 83.2)	348.1 (334.3 to 362.3)	84.3 (81.0 to 87.5)	0.17 (0.16 to 0.17)
Bahrain	273.3 (260.5 to 286.3)	58.2 (56.0 to 60.4)	1142.5 (1094.7 to 1193.5)	68.5 (65.7 to 71.0)	0.53 (0.53 to 0.53)

Bangladesh	40314.8 (38836.5 to 41792.1)	42.7 (41.2 to 44.3)	80010.6 (77338.6 to 82886.9)	48.6 (47.0 to 50.3)	0.42 (0.42 to 0.43)
Barbados	200.9 (192.5 to 209.0)	80.2 (76.9 to 83.4)	280.5 (269.1 to 292.4)	83.4 (80.3 to 86.6)	0.13 (0.12 to 0.13)
Belarus	5759.7 (5540.2 to 6023.6)	51.4 (49.6 to 53.7)	6235.6 (5979.0 to 6502.3)	57.9 (55.6 to 60.2)	0.38 (0.38 to 0.39)
Belgium	11495.6 (11115.1 to 11882.1)	106.0 (102.7 to 109.7)	15178.7 (14643.5 to 15715.6)	118.8 (115.0 to 122.9)	0.35 (0.33 to 0.36)
Belize	113.0 (108.1 to 117.3)	69.7 (67.0 to 72.4)	319.9 (306.2 to 332.7)	76.7 (73.6 to 79.7)	0.31 (0.3 to 0.31)
Benin	1108.4 (1066.7 to 1148.8)	27.6 (26.6 to 28.6)	3402.7 (3266.7 to 3533.2)	29.6 (28.6 to 30.6)	0.22 (0.21 to 0.22)
Bermuda	48.8 (46.9 to 50.9)	78.4 (75.4 to 81.6)	61.6 (58.8 to 64.4)	83.5 (80.1 to 86.8)	0.21 (0.2 to 0.21)
Bhutan	226.2 (217.7 to 234.7)	41.2 (39.8 to 42.6)	362.5 (350.2 to 376.5)	47.6 (46.0 to 49.3)	0.47 (0.47 to 0.47)
Bolivia (Plurinational State of)	4997.6 (4788.4 to 5181.1)	87.0 (83.7 to 90.1)	11553.9 (11106.9 to 12029.1)	99.6 (95.8 to 103.6)	0.44 (0.44 to 0.44)
Bosnia and Herzegovina	2373.9 (2286.5 to 2460.1)	50.5 (48.7 to 52.2)	2274.3 (2194.0 to 2365.2)	59.2 (57.2 to 61.4)	0.53 (0.52 to 0.54)
Botswana	344.7 (329.6 to 358.7)	30.4 (29.3 to 31.5)	797.9 (763.2 to 831.4)	34.1 (32.7 to 35.4)	0.36 (0.36 to 0.37)

Brazil	79423.9 (76660.2 to 81803.3)	57.6 (55.7 to 59.3)	134574.7 (130267.1 to 138820.6)	57.8 (55.9 to 59.5)	0.01 (0.01 to 0.02)
Brunei Darussalam	122.8 (118.2 to 128.3)	53.7 (51.9 to 55.8)	280.9 (270.2 to 291.6)	59.1 (57.0 to 61.2)	0.29 (0.28 to 0.31)
Bulgaria	5273.9 (5090.7 to 5473.9)	54.1 (52.3 to 56.1)	4774.0 (4597.0 to 4958.3)	60.7 (58.6 to 63.0)	0.37 (0.37 to 0.38)
Burkina Faso	2098.6 (2012.1 to 2177.8)	26.3 (25.3 to 27.2)	5459.9 (5246.5 to 5659.0)	28.2 (27.2 to 29.2)	0.23 (0.22 to 0.24)
Burundi	987.6 (946.7 to 1024.5)	21.7 (20.9 to 22.5)	2341.0 (2251.2 to 2431.1)	21.1 (20.4 to 21.8)	-0.09 (-0.1 to -0.08)
Cabo Verde	86.3 (83.0 to 89.3)	28.3 (27.3 to 29.3)	178.3 (171.9 to 184.4)	31.7 (30.6 to 32.7)	0.36 (0.35 to 0.37)
Cambodia	4282.6 (4108.6 to 4458.2)	51.7 (49.8 to 53.9)	10010.2 (9612.6 to 10475.9)	61.2 (58.7 to 63.9)	0.55 (0.54 to 0.55)
Cameroon	2595.1 (2492.2 to 2686.8)	29.2 (28.2 to 30.3)	8825.9 (8457.3 to 9148.1)	31.7 (30.6 to 32.8)	0.26 (0.24 to 0.28)
Canada	24971.3 (24127.6 to 25850.8)	87.8 (84.9 to 90.8)	41412.3 (39975.4 to 42799.3)	98.9 (95.6 to 102.1)	0.37 (0.35 to 0.39)
Central African Republic	795.8 (762.1 to 829.8)	33.5 (32.2 to 35.0)	1778.7 (1702.5 to 1855.3)	36.4 (34.9 to 38.0)	0.27 (0.27 to 0.27)
Chad	1301.1 (1251.2 to 1348.3)	25.9 (25.0 to 26.9)	3948.4 (3797.7 to 4085.0)	27.3 (26.4 to 28.2)	0.17 (0.16 to 0.18)

Chile	8579.5<br><br>(8252.7 to 8922.6)	67.3<br><br>(64.8 to 69.9)	16069.9<br><br>(15439.4 to 16741.6)	78.0<br><br>(74.8 to 81.0)	0.46<br><br>(0.44 to 0.48)
China	530265.1<br><br>(513660.3 to 548625.6)	47.5<br><br>(46.0 to 48.9)	1012635.2<br><br>(979126.5 to 1044617.1)	59.7<br><br>(57.8 to 61.6)	0.74<br><br>(0.74 to 0.75)
Colombia	22052.2<br><br>(21170.2 to 22948.9)	74.3<br><br>(71.5 to 77.3)	41207.3<br><br>(39513.2 to 42890.1)	80.7<br><br>(77.4 to 83.8)	0.27<br><br>(0.27 to 0.28)
Comoros	85.4<br><br>(82.1 to 88.6)	22.2<br><br>(21.5 to 23.0)	162.8<br><br>(156.9 to 168.2)	23.1<br><br>(22.3 to 23.9)	0.12<br><br>(0.11 to 0.13)
Congo	794.9<br><br>(759.2 to 826.6)	37.9<br><br>(36.4 to 39.3)	2212.1<br><br>(2113.6 to 2302.0)	43.6<br><br>(41.8 to 45.2)	0.45<br><br>(0.45 to 0.46)
Cook Islands	9.3<br><br>(8.9 to 9.6)	54.2<br><br>(52.0 to 56.2)	12.2<br><br>(11.7 to 12.7)	61.7<br><br>(59.4 to 64.1)	0.42<br><br>(0.42 to 0.43)
Costa Rica	2101.2<br><br>(2011.1 to 2182.9)	75.7<br><br>(72.7 to 78.5)	4092.2<br><br>(3935.5 to 4251.2)	81.8<br><br>(78.7 to 84.9)	0.25<br><br>(0.25 to 0.26)
Coted'Ivoire	2907.0<br><br>(2792.0 to 3011.2)	28.3<br><br>(27.3 to 29.2)	7512.6<br><br>(7217.6 to 7795.1)	30.5<br><br>(29.4 to 31.5)	0.24<br><br>(0.23 to 0.25)
Croatia	2958.5<br><br>(2848.3 to 3064.4)	54.8<br><br>(52.8 to 56.8)	2964.6<br><br>(2856.6 to 3074.0)	61.3<br><br>(59.1 to 63.4)	0.38<br><br>(0.37 to 0.38)
Cuba	8125.6<br><br>(7783.5 to 8458.8)	75.4<br><br>(72.3 to 78.5)	10070.4<br><br>(9637.7 to 10516.2)	78.6<br><br>(75.4 to 81.7)	0.13<br><br>(0.13 to 0.14)
Cyprus	839.1<br><br>(808.0 to	105.0<br><br>(101.2 to	1844.7<br><br>(1779.9 to	121.9<br><br>(118.0 to	0.45<br><br>(0.44 to

	867.3)	108.5)	1912.2)	126.3)	0.47)
Czechia	6106.0 (5903.7 to 6320.8)	54.8 (52.9 to 56.7)	7434.0 (7168.5 to 7724.5)	61.4 (59.3 to 63.7)	0.37 (0.37 to 0.38)
Democratic People's Republic of Korea	7007.8 (6754.4 to 7259.9)	35.2 (34.0 to 36.5)	12648.2 (12199.4 to 13089.3)	42.1 (40.7 to 43.5)	0.58 (0.58 to 0.58)
Democratic Republic of the Congo	11577.5 (11085.4 to 12058.8)	35.3 (33.9 to 36.7)	30831.2 (29464.1 to 32188.9)	38.6 (36.9 to 40.2)	0.29 (0.28 to 0.3)
Denmark	6024.9 (5815.6 to 6251.8)	108.4 (104.6 to 111.9)	7803.1 (7534.7 to 8101.3)	119.9 (115.8 to 124.3)	0.31 (0.3 to 0.32)
Djibouti	76.4 (73.3 to 79.3)	22.0 (21.3 to 22.7)	266.5 (256.0 to 276.3)	22.6 (21.8 to 23.4)	0.09 (0.08 to 0.09)
Dominica	51.0 (49.0 to 53.1)	75.7 (72.7 to 78.7)	59.5 (56.9 to 61.9)	82.6 (79.2 to 85.7)	0.29 (0.28 to 0.29)
Dominican Republic	4431.3 (4248.3 to 4612.6)	69.3 (66.4 to 72.1)	8353.9 (8018.4 to 8698.1)	76.0 (73.0 to 79.2)	0.3 (0.3 to 0.3)
Ecuador	8601.1 (8270.6 to 8928.8)	94.6 (91.1 to 98.2)	19231.0 (18482.7 to 19946.6)	107.0 (102.7 to 110.9)	0.4 (0.4 to 0.4)
Egypt	17801.8 (17080.9 to 18430.4)	37.1 (35.8 to 38.2)	44010.2 (42289.5 to 45716.2)	44.4 (42.7 to 46.1)	0.59 (0.58 to 0.6)
El Salvador	3241.1 (3105.2 to 3367.6)	68.8 (66.2 to 71.7)	4845.6 (4652.4 to 5050.9)	76.2 (73.3 to 79.4)	0.34 (0.33 to 0.34)

Equatorial Guinea	125.5 (120.3 to 130.4)	34.3 (32.9 to 35.6)	622.6 (594.4 to 650.0)	46.2 (44.3 to 48.0)	0.97 (0.96 to 0.97)
Eritrea	592.8 (568.8 to 615.2)	21.2 (20.4 to 21.9)	1284.4 (1237.2 to 1330.2)	22.1 (21.4 to 22.9)	0.13 (0.12 to 0.14)
Estonia	877.4 (843.3 to 914.8)	51.9 (49.8 to 54.0)	869.1 (831.8 to 905.3)	59.2 (56.7 to 61.6)	0.43 (0.42 to 0.44)
Eswatini	199.3 (189.9 to 207.8)	29.6 (28.4 to 30.8)	345.7 (331.9 to 359.6)	32.6 (31.4 to 33.9)	0.31 (0.3 to 0.31)
Ethiopia	9746.8 (9364.8 to 10072.2)	23.6 (22.9 to 24.4)	22743.3 (21896.2 to 23552.2)	24.7 (23.9 to 25.4)	0.14 (0.14 to 0.14)
Fiji	335.7 (322.2 to 348.7)	51.0 (49.1 to 53.1)	539.1 (515.8 to 562.7)	58.4 (55.9 to 60.9)	0.44 (0.43 to 0.44)
Finland	5633.2 (5433.0 to 5824.5)	104.0 (100.5 to 107.4)	7138.5 (6873.3 to 7404.3)	115.9 (111.9 to 119.9)	0.34 (0.32 to 0.35)
France	64499.1 (62214.2 to 66664.2)	105.5 (101.9 to 109.0)	86918.0 (83869.0 to 90067.5)	119.2 (115.1 to 123.5)	0.38 (0.35 to 0.4)
Gabon	338.7 (323.9 to 351.9)	38.4 (36.8 to 40.1)	782.5 (751.4 to 816.0)	45.6 (43.9 to 47.4)	0.56 (0.55 to 0.56)
Gambia	227.6 (218.2 to 235.7)	27.7 (26.7 to 28.7)	625.7 (600.5 to 648.5)	30.0 (28.9 to 31.0)	0.26 (0.25 to 0.27)
Georgia	3142.9 (3014.6 to 3263.8)	54.1 (52.0 to 56.1)	2296.6 (2205.9 to 2392.6)	57.4 (55.2 to 59.8)	0.2 (0.19 to 0.2)

Germany	112786.8 (108585.0 to 116791.4)	128.2 (123.4 to 132.3)	137846.5 (132932.6 to 142995.1)	143.6 (139.0 to 148.7)	0.35 (0.33 to 0.37)
Ghana	3592.0 (3411.7 to 3750.1)	27.9 (26.7 to 29.1)	9533.9 (9096.6 to 9962.5)	30.6 (29.3 to 32.0)	0.29 (0.29 to 0.3)
Greece	11903.1 (11483.4 to 12329.5)	105.1 (101.6 to 108.7)	13566.8 (13059.8 to 14086.4)	118.0 (113.9 to 121.8)	0.36 (0.34 to 0.37)
Greenland	45.8 (44.2 to 47.6)	83.4 (80.5 to 86.1)	59.2 (57.2 to 61.2)	95.0 (91.9 to 98.1)	0.42 (0.39 to 0.43)
Grenada	56.9 (54.6 to 59.2)	71.9 (69.0 to 74.7)	87.6 (84.0 to 91.0)	80.4 (77.3 to 83.4)	0.36 (0.36 to 0.37)
Guam	71.9 (68.9 to 74.7)	57.4 (55.1 to 59.6)	110.7 (106.3 to 115.1)	64.2 (61.7 to 66.7)	0.37 (0.36 to 0.37)
Guatemala	4687.3 (4487.4 to 4883.3)	65.4 (62.7 to 67.9)	11029.1 (10558.4 to 11468.8)	73.6 (70.5 to 76.3)	0.38 (0.38 to 0.38)
Guinea	1403.8 (1352.4 to 1455.4)	27.1 (26.2 to 28.1)	3352.9 (3221.2 to 3491.4)	29.1 (28.1 to 30.1)	0.23 (0.22 to 0.24)
Guinea-Bissau	231.9 (223.0 to 240.2)	27.5 (26.5 to 28.4)	524.6 (503.8 to 544.6)	29.5 (28.5 to 30.6)	0.22 (0.21 to 0.23)
Guyana	502.8 (481.6 to 522.6)	72.5 (69.4 to 75.3)	611.4 (586.4 to 636.0)	80.0 (76.7 to 83.2)	0.31 (0.31 to 0.32)
Haiti	3654.3 (3503.2 to 3802.6)	64.5 (62.0 to 67.1)	8340.0 (8006.5 to 8697.4)	69.0 (66.2 to 71.8)	0.22 (0.21 to 0.22)

Honduras	2679.4 (2549.3 to 2786.5)	66.3 (63.7 to 68.9)	6952.7 (6652.2 to 7227.3)	73.1 (70.0 to 76.0)	0.31 (0.31 to 0.32)
Hungary	6217.1 (5998.4 to 6436.1)	54.2 (52.2 to 56.1)	6711.8 (6463.8 to 6951.0)	61.0 (58.8 to 63.1)	0.39 (0.38 to 0.4)
Iceland	268.4 (259.2 to 277.6)	104.5 (101.0 to 108.1)	443.9 (427.9 to 461.1)	116.3 (112.1 to 120.6)	0.33 (0.31 to 0.34)
India	365117.6 (352852.3 to 376465.1)	46.2 (44.8 to 47.7)	712329.7 (689472.1 to 736325.1)	49.8 (48.2 to 51.4)	0.22 (0.17 to 0.25)
Indonesia	95965.3 (92842.5 to 99054.0)	59.0 (57.2 to 60.8)	201082.1 (194859.0 to 207346.1)	69.2 (67.1 to 71.3)	0.52 (0.51 to 0.52)
Iran (Islamic Republic of)	25363.1 (24536.9 to 26113.9)	53.3 (51.7 to 54.9)	59099.6 (57233.2 to 61106.6)	65.0 (63.0 to 67.1)	0.65 (0.64 to 0.65)
Iraq	9224.4 (8873.9 to 9557.6)	61.6 (59.3 to 63.9)	29883.5 (28668.1 to 31167.2)	76.3 (73.5 to 79.3)	0.69 (0.69 to 0.7)
Ireland	3791.3 (3654.0 to 3916.4)	104.5 (100.7 to 108.0)	6442.2 (6215.7 to 6685.2)	119.1 (115.2 to 123.4)	0.41 (0.39 to 0.42)
Israel	5240.5 (5061.6 to 5427.8)	108.6 (104.9 to 112.5)	11658.9 (11263.7 to 12084.6)	120.2 (116.2 to 124.7)	0.31 (0.29 to 0.32)
Italy	57781.8 (55914.7 to 59647.4)	92.1 (88.9 to 95.0)	69353.8 (66965.1 to 71699.4)	99.8 (96.6 to 103.0)	0.24 (0.23 to 0.26)
Jamaica	1607.9 (1540.7 to 1672.5)	74.0 (71.0 to 76.9)	2312.9 (2223.9 to 2407.3)	79.7 (76.6 to 82.9)	0.24 (0.24 to 0.24)

Japan	75051.8 (72682.9 to 77408.9)	53.5 (51.8 to 55.1)	85415.9 (82685.7 to 88125.6)	56.3 (54.5 to 58.1)	0.15 (0.13 to 0.16)
Jordan	1710.6 (1641.1 to 1775.0)	56.4 (54.1 to 58.4)	7944.6 (7603.9 to 8255.3)	66.0 (63.5 to 68.3)	0.51 (0.51 to 0.51)
Kazakhstan	8149.6 (7816.5 to 8489.0)	51.5 (49.4 to 53.6)	11137.5 (10700.2 to 11600.9)	57.2 (54.9 to 59.5)	0.34 (0.33 to 0.34)
Kenya	4336.9 (4174.7 to 4478.7)	23.2 (22.5 to 24.0)	10669.0 (10284.0 to 11027.3)	24.0 (23.3 to 24.8)	0.1 (0.1 to 0.11)
Kiribati	30.9 (29.7 to 32.1)	49.7 (47.9 to 51.5)	61.7 (59.2 to 64.1)	55.8 (53.5 to 57.8)	0.37 (0.37 to 0.38)
Kuwait	977.1 (937.3 to 1023.8)	61.0 (58.8 to 63.5)	3674.7 (3507.1 to 3848.0)	71.3 (68.5 to 74.0)	0.5 (0.5 to 0.51)
Kyrgyzstan	2046.3 (1967.0 to 2125.5)	50.7 (48.7 to 52.7)	3607.9 (3454.4 to 3759.9)	54.3 (52.1 to 56.6)	0.23 (0.22 to 0.24)
Lao People's Democratic Republic	1758.4 (1684.9 to 1833.0)	51.1 (49.2 to 53.3)	4215.3 (4047.7 to 4391.8)	61.2 (58.9 to 63.8)	0.58 (0.58 to 0.59)
Latvia	1512.8 (1452.8 to 1573.3)	52.4 (50.4 to 54.5)	1253.7 (1200.5 to 1304.4)	59.0 (56.5 to 61.5)	0.38 (0.37 to 0.39)
Lebanon	1514.1 (1458.9 to 1567.5)	54.3 (52.3 to 56.3)	3775.2 (3634.0 to 3928.2)	65.2 (62.9 to 67.8)	0.59 (0.59 to 0.6)
Lesotho	378.7 (362.8 to 394.0)	28.2 (27.0 to 29.3)	540.9 (516.8 to 563.6)	31.0 (29.7 to 32.2)	0.31 (0.3 to 0.31)

Liberia	583.7 (560.8 to 604.2)	27.8 (26.9 to 28.8)	1446.5 (1390.9 to 1503.3)	29.8 (28.8 to 30.8)	0.23 (0.22 to 0.24)
Libya	1925.2 (1847.3 to 1995.4)	54.9 (52.9 to 57.0)	4838.0 (4652.1 to 5042.5)	66.3 (64.0 to 68.9)	0.6 (0.6 to 0.61)
Lithuania	2040.9 (1957.4 to 2125.8)	52.3 (50.2 to 54.3)	1878.6 (1798.2 to 1959.7)	60.4 (58.1 to 62.8)	0.47 (0.47 to 0.48)
Luxembourg	452.9 (438.2 to 468.3)	108.8 (105.2 to 112.3)	881.0 (851.3 to 913.5)	121.0 (117.2 to 125.2)	0.33 (0.31 to 0.34)
Madagascar	2136.3 (2049.5 to 2213.3)	21.7 (20.9 to 22.4)	5316.5 (5085.2 to 5498.4)	21.6 (20.8 to 22.3)	-0.01 (-0.02 to 0)
Malawi	1710.0 (1645.3 to 1772.0)	21.4 (20.6 to 22.1)	3595.4 (3437.7 to 3733.6)	21.9 (21.1 to 22.7)	0.08 (0.07 to 0.08)
Malaysia	9695.7 (9311.2 to 10106.2)	62.9 (60.4 to 65.3)	23678.7 (22654.5 to 24645.7)	72.8 (69.7 to 75.7)	0.48 (0.48 to 0.48)
Maldives	96.2 (92.0 to 99.9)	55.0 (52.8 to 57.0)	379.8 (363.0 to 397.6)	69.3 (66.6 to 72.0)	0.75 (0.75 to 0.75)
Mali	2012.1 (1909.2 to 2101.3)	27.4 (26.1 to 28.6)	5953.0 (5667.6 to 6245.9)	29.6 (28.3 to 30.9)	0.26 (0.24 to 0.27)
Malta	408.1 (394.1 to 421.6)	104.9 (101.5 to 108.2)	593.0 (571.6 to 614.1)	119.3 (115.3 to 123.2)	0.4 (0.38 to 0.41)
Marshall Islands	17.2 (16.5 to 18.0)	49.3 (47.3 to 51.2)	29.8 (28.7 to 31.1)	55.6 (53.4 to 57.8)	0.39 (0.39 to 0.39)

Mauritania	504.2 (484.3 to 522.2)	28.6 (27.5 to 29.5)	1205.8 (1159.2 to 1249.2)	31.1 (30.0 to 32.2)	0.27 (0.26 to 0.28)
Mauritius	620.0 (593.8 to 645.9)	60.4 (58.0 to 63.0)	1023.4 (983.1 to 1065.3)	69.5 (66.8 to 72.2)	0.45 (0.45 to 0.46)
Mexico	55532.3 (53580.2 to 57223.3)	72.9 (70.7 to 75.2)	104805.2 (101456.6 to 108043.9)	78.9 (76.3 to 81.3)	0.26 (0.25 to 0.26)
Micronesia (Federated States of)	42.2 (40.5 to 43.8)	50.4 (48.3 to 52.4)	56.3 (54.1 to 58.5)	57.0 (54.9 to 59.3)	0.4 (0.4 to 0.4)
Monaco	39.3 (37.8 to 40.7)	113.7 (109.8 to 117.8)	54.1 (52.1 to 56.3)	124.1 (120.0 to 128.2)	0.27 (0.25 to 0.28)
Mongolia	871.6 (834.5 to 908.7)	47.4 (45.6 to 49.3)	1756.4 (1681.6 to 1826.5)	53.4 (51.2 to 55.6)	0.39 (0.38 to 0.39)
Montenegro	357.8 (345.5 to 369.6)	55.4 (53.4 to 57.3)	427.0 (411.4 to 443.0)	61.2 (59.2 to 63.5)	0.33 (0.32 to 0.34)
Morocco	11428.9 (11001.4 to 11886.9)	51.5 (49.5 to 53.6)	23291.8 (22440.6 to 24291.0)	60.9 (58.7 to 63.4)	0.54 (0.54 to 0.54)
Mozambique	2265.3 (2175.3 to 2346.9)	20.2 (19.5 to 20.9)	5361.4 (5139.6 to 5563.2)	20.9 (20.2 to 21.6)	0.11 (0.1 to 0.12)
Myanmar	18221.0 (17518.3 to 18960.9)	51.5 (49.6 to 53.5)	34761.8 (33339.6 to 36095.4)	61.6 (59.1 to 64.0)	0.58 (0.58 to 0.58)
Namibia	376.4 (360.8 to 391.2)	30.9 (29.6 to 32.0)	764.4 (731.2 to 794.2)	33.5 (32.1 to 34.8)	0.26 (0.25 to 0.27)

Nauru	4.5<br><br>(4.4 to 4.7)	53.1<br><br>(51.0 to 55.2)	5.8<br><br>(5.6 to 6.0)	59.6<br><br>(57.4 to 61.9)	0.37<br><br>(0.37 to 0.38)
Nepal	6697.4<br><br>(6451.0 to 6938.5)	38.9<br><br>(37.6 to 40.3)	14420.9<br><br>(13902.0 to 14920.3)	47.6<br><br>(45.9 to 49.2)	0.64<br><br>(0.63 to 0.65)
Netherlands	17325.5<br><br>(16733.9 to 17953.9)	108.7<br><br>(105.1 to 112.3)	23404.9<br><br>(22490.1 to 24249.4)	120.5<br><br>(116.0 to 124.5)	0.31<br><br>(0.29 to 0.32)
New Zealand	2319.8<br><br>(2242.5 to 2398.9)	65.9<br><br>(63.7 to 68.1)	4247.4<br><br>(4094.2 to 4382.5)	74.3<br><br>(71.5 to 76.7)	0.37<br><br>(0.35 to 0.39)
Nicaragua	2212.7<br><br>(2111.2 to 2302.4)	66.9<br><br>(64.2 to 69.6)	4795.7<br><br>(4603.1 to 4991.3)	74.0<br><br>(71.1 to 76.9)	0.33<br><br>(0.32 to 0.33)
Niger	1659.4<br><br>(1596.6 to 1717.1)	25.2<br><br>(24.3 to 26.1)	5241.6<br><br>(5039.5 to 5449.7)	25.8<br><br>(25.0 to 26.7)	0.08<br><br>(0.07 to 0.09)
Nigeria	38730.0<br><br>(37360.6 to 39857.9)	48.5<br><br>(46.9 to 50.0)	115536.7<br><br>(111283.9 to 119031.2)	56.3<br><br>(54.5 to 58.0)	0.49<br><br>(0.48 to 0.49)
Niue	1.1<br><br>(1.1 to 1.2)	52.2<br><br>(50.0 to 54.3)	1.1<br><br>(1.1 to 1.1)	60.0<br><br>(57.7 to 62.3)	0.45<br><br>(0.45 to 0.46)
North Macedonia	1098.5<br><br>(1059.8 to 1138.1)	53.6<br><br>(51.7 to 55.5)	1519.3<br><br>(1459.9 to 1576.7)	60.3<br><br>(58.2 to 62.6)	0.39<br><br>(0.38 to 0.4)
Northern Mariana Islands	24.6<br><br>(23.5 to 25.7)	59.0<br><br>(56.8 to 61.2)	34.1<br><br>(32.6 to 35.5)	63.3<br><br>(60.7 to 65.6)	0.22<br><br>(0.21 to 0.23)
Norway	3943.6<br><br>(3825.0 to 4072.1)	87.3<br><br>(84.7 to 90.2)	5684.0<br><br>(5501.3 to 5871.9)	94.0<br><br>(91.1 to 97.1)	0.24<br><br>(0.22 to 0.25)

Oman	925.4 (885.7 to 963.7)	55.3 (53.2 to 57.4)	3334.3 (3192.9 to 3501.1)	69.9 (67.4 to 72.7)	0.76 (0.76 to 0.76)
Pakistan	40870.2 (39330.3 to 42219.7)	41.9 (40.6 to 43.3)	103853.7 (100121.1 to 107327.8)	47.3 (45.7 to 48.8)	0.39 (0.39 to 0.39)
Palau	7.9 (7.6 to 8.2)	56.0 (53.7 to 58.1)	13.6 (13.0 to 14.2)	63.2 (60.9 to 65.7)	0.39 (0.39 to 0.4)
Palestine	811.6 (782.2 to 843.9)	50.4 (48.6 to 52.3)	2845.1 (2739.0 to 2957.5)	61.2 (58.9 to 63.5)	0.63 (0.63 to 0.63)
Panama	1686.4 (1615.0 to 1750.6)	76.3 (73.2 to 79.3)	3576.8 (3431.8 to 3713.0)	82.3 (79.0 to 85.5)	0.25 (0.25 to 0.25)
Papua New Guinea	1545.7 (1481.3 to 1608.4)	45.9 (44.1 to 47.7)	4704.4 (4499.8 to 4891.7)	51.5 (49.3 to 53.6)	0.38 (0.38 to 0.38)
Paraguay	2023.0 (1946.5 to 2090.2)	56.2 (54.2 to 58.0)	4084.1 (3945.8 to 4225.6)	58.0 (56.0 to 59.9)	0.1 (0.1 to 0.1)
Peru	18349.8 (17653.2 to 19028.8)	92.8 (89.3 to 96.0)	38530.3 (37066.3 to 39993.6)	105.8 (101.8 to 109.8)	0.43 (0.42 to 0.43)
Philippines	31640.8 (30606.0 to 32603.0)	59.6 (57.9 to 61.5)	72611.4 (70392.7 to 74978.4)	66.8 (64.8 to 68.9)	0.37 (0.37 to 0.37)
Poland	23518.0 (22779.8 to 24318.3)	58.6 (56.8 to 60.5)	27814.5 (27097.4 to 28554.5)	63.9 (62.4 to 65.5)	0.29 (0.28 to 0.29)
Portugal	10963.5 (10573.8 to 11357.7)	100.7 (97.2 to 104.2)	13895.1 (13400.1 to 14413.2)	114.7 (110.8 to 118.7)	0.4 (0.38 to 0.41)

Puerto Rico	2881.1 (2764.3 to 2995.3)	79.9 (76.7 to 83.1)	3179.3 (3042.7 to 3311.2)	86.6 (83.2 to 90.0)	0.26 (0.26 to 0.27)
Qatar	256.7 (244.1 to 269.4)	59.7 (57.4 to 62.0)	2345.3 (2224.5 to 2473.0)	71.8 (68.9 to 74.6)	0.6 (0.6 to 0.6)
Republic of Korea	21863.6 (20958.6 to 22742.2)	50.3 (48.3 to 52.2)	36302.1 (34883.4 to 37742.4)	57.2 (55.1 to 59.3)	0.39 (0.37 to 0.41)
Republic of Moldova	2301.5 (2208.7 to 2397.9)	50.5 (48.5 to 52.6)	2359.7 (2252.8 to 2466.1)	56.6 (54.2 to 58.9)	0.37 (0.36 to 0.38)
Romania	13290.5 (12809.1 to 13793.2)	52.9 (51.1 to 54.8)	12665.4 (12228.6 to 13135.7)	58.4 (56.2 to 60.3)	0.32 (0.32 to 0.33)
Russian Federation	85725.0 (82967.5 to 88625.5)	52.9 (51.3 to 54.7)	97498.9 (94380.2 to 100750.2)	58.9 (57.1 to 60.9)	0.35 (0.34 to 0.35)
Rwanda	1065.9 (1012.7 to 1114.4)	18.2 (17.4 to 19.1)	2225.1 (2122.9 to 2326.3)	18.9 (18.1 to 19.7)	0.11 (0.09 to 0.13)
Saint Kitts and Nevis	29.5 (28.3 to 30.7)	76.4 (73.2 to 79.4)	54.4 (52.1 to 56.7)	83.2 (79.9 to 86.5)	0.28 (0.28 to 0.28)
Saint Lucia	89.7 (85.7 to 93.6)	72.6 (69.4 to 75.5)	156.6 (150.3 to 163.6)	79.8 (76.7 to 83.2)	0.31 (0.31 to 0.31)
Saint Vincent and the Grenadines	70.9 (67.9 to 73.9)	71.6 (68.6 to 74.5)	95.4 (91.8 to 99.3)	78.1 (75.0 to 81.2)	0.28 (0.28 to 0.28)
Samoa	71.3 (68.3 to 74.1)	51.8 (49.8 to 53.9)	111.3 (106.8 to 115.5)	58.2 (55.8 to 60.3)	0.38 (0.37 to 0.38)

San Marino	28.7 (27.7 to 29.7)	111.9 (108.0 to 115.8)	45.8 (44.1 to 47.7)	122.0 (117.7 to 126.4)	0.27 (0.25 to 0.28)
Sao Tome and Principe	30.5 (29.3 to 31.6)	29.4 (28.4 to 30.4)	64.7 (62.2 to 66.8)	32.2 (31.1 to 33.3)	0.3 (0.29 to 0.31)
Saudi Arabia	7570.1 (7253.9 to 7856.2)	56.6 (54.5 to 58.7)	28543.0 (27268.8 to 29851.5)	70.8 (68.3 to 73.4)	0.72 (0.72 to 0.73)
Senegal	1772.8 (1705.2 to 1835.9)	27.7 (26.7 to 28.6)	4241.6 (4079.2 to 4381.3)	30.1 (29.0 to 31.0)	0.26 (0.25 to 0.27)
Serbia	5711.9 (5494.0 to 5923.1)	54.1 (52.0 to 55.9)	6197.7 (5984.5 to 6427.3)	60.9 (58.8 to 63.0)	0.39 (0.39 to 0.4)
Seychelles	42.6 (40.6 to 44.2)	64.1 (61.5 to 66.5)	84.0 (80.5 to 87.6)	72.7 (69.7 to 75.7)	0.4 (0.4 to 0.41)
Sierra Leone	990.2 (950.4 to 1026.4)	27.7 (26.7 to 28.7)	2291.7 (2211.7 to 2371.5)	29.4 (28.4 to 30.4)	0.2 (0.18 to 0.21)
Singapore	1668.0 (1602.3 to 1739.3)	54.3 (52.3 to 56.4)	4177.7 (4006.0 to 4337.3)	61.2 (58.8 to 63.4)	0.38 (0.36 to 0.39)
Slovakia	2977.1 (2872.4 to 3076.7)	54.1 (52.2 to 55.9)	3793.9 (3647.8 to 3926.6)	61.0 (58.8 to 63.0)	0.39 (0.39 to 0.4)
Slovenia	1198.3 (1157.6 to 1241.4)	56.2 (54.2 to 58.2)	1492.8 (1435.6 to 1548.3)	62.7 (60.4 to 64.9)	0.36 (0.35 to 0.36)
Solomon Islands	119.3 (114.1 to 124.2)	45.0 (43.2 to 46.8)	304.3 (292.1 to 317.4)	51.1 (49.2 to 53.2)	0.41 (0.41 to 0.42)

Somalia	1261.8 (1209.8 to 1307.0)	19.7 (19.0 to 20.3)	3214.5 (3084.4 to 3340.0)	18.6 (17.9 to 19.2)	-0.2 (-0.21 to -0.19)
South Africa	10940.1 (10546.1 to 11305.8)	32.5 (31.4 to 33.6)	19796.9 (19146.5 to 20458.7)	34.5 (33.4 to 35.6)	0.19 (0.19 to 0.19)
South Sudan	1052.3 (1011.3 to 1089.9)	21.6 (20.8 to 22.3)	1704.4 (1636.7 to 1766.8)	20.9 (20.2 to 21.7)	-0.1 (-0.11 to -0.09)
Spain	32879.3 (31713.4 to 34047.4)	78.9 (76.2 to 81.7)	45677.6 (44061.9 to 47212.5)	86.7 (84.0 to 89.5)	0.28 (0.26 to 0.29)
Sri Lanka	9735.5 (9338.8 to 10126.3)	61.3 (58.9 to 63.7)	17118.4 (16422.3 to 17856.4)	70.8 (67.8 to 73.7)	0.47 (0.47 to 0.47)
Sudan	8132.2 (7816.8 to 8440.9)	49.7 (47.9 to 51.5)	23666.8 (22674.3 to 24593.4)	61.8 (59.5 to 64.0)	0.71 (0.71 to 0.71)
Suriname	272.8 (261.5 to 283.9)	75.2 (72.1 to 78.1)	486.6 (466.9 to 506.7)	80.3 (77.1 to 83.5)	0.22 (0.21 to 0.22)
Sweden	8236.2 (7959.9 to 8501.7)	88.7 (85.6 to 91.6)	10761.2 (10408.0 to 11124.8)	94.3 (91.3 to 97.4)	0.18 (0.16 to 0.2)
Switzerland	8508.5 (8216.0 to 8808.6)	114.4 (110.3 to 118.3)	12563.2 (12118.2 to 13012.0)	124.8 (120.7 to 129.1)	0.27 (0.25 to 0.28)
Syrian Arab Republic	5253.0 (5055.4 to 5424.1)	51.6 (49.8 to 53.4)	9112.4 (8716.2 to 9485.7)	62.8 (60.4 to 65.2)	0.64 (0.64 to 0.64)
Taiwan (Province of China)	6382.1 (6153.2 to 6626.6)	32.1 (31.0 to 33.2)	11951.1 (11511.4 to 12345.9)	40.1 (38.8 to 41.4)	0.72 (0.72 to 0.73)

Tajikistan	2213.0<br><br>(2124.7 to 2311.0)	48.9<br><br>(47.1 to 51.0)	4998.6<br><br>(4804.4 to 5186.9)	52.7<br><br>(50.7 to 54.8)	0.25<br><br>(0.24 to 0.25)
Thailand	31376.7<br><br>(30027.0 to 32613.1)	59.3<br><br>(57.0 to 61.6)	54850.9<br><br>(52543.7 to 57097.6)	68.4<br><br>(65.6 to 71.0)	0.46<br><br>(0.45 to 0.46)
Timor-Leste	330.9<br><br>(316.3 to 345.3)	52.6<br><br>(50.4 to 54.8)	769.8<br><br>(737.0 to 799.7)	63.4<br><br>(60.8 to 66.0)	0.61<br><br>(0.61 to 0.61)
Togo	863.1<br><br>(828.0 to 893.7)	28.5<br><br>(27.4 to 29.4)	2301.2<br><br>(2215.8 to 2388.5)	30.4<br><br>(29.4 to 31.5)	0.21<br><br>(0.2 to 0.22)
Tokelau	0.7<br><br>(0.7 to 0.7)	49.2<br><br>(47.2 to 51.0)	0.8<br><br>(0.8 to 0.9)	58.7<br><br>(56.4 to 60.9)	0.57<br><br>(0.57 to 0.57)
Tonga	41.2<br><br>(39.6 to 42.8)	50.1<br><br>(48.2 to 52.1)	55.1<br><br>(52.9 to 57.4)	57.1<br><br>(54.9 to 59.4)	0.42<br><br>(0.42 to 0.43)
Trinidad and Tobago	904.6<br><br>(868.1 to 940.7)	79.2<br><br>(76.1 to 82.4)	1302.3<br><br>(1246.1 to 1357.2)	85.4<br><br>(81.8 to 89.0)	0.25<br><br>(0.24 to 0.25)
Tunisia	4004.6<br><br>(3860.6 to 4157.4)	53.6<br><br>(51.8 to 55.7)	8214.2<br><br>(7872.4 to 8541.3)	64.5<br><br>(61.8 to 66.9)	0.6<br><br>(0.6 to 0.6)
Turkey	33716.2<br><br>(32438.2 to 34937.9)	64.3<br><br>(62.0 to 66.7)	71700.0<br><br>(69085.5 to 74506.5)	79.7<br><br>(76.8 to 82.8)	0.7<br><br>(0.69 to 0.7)
Turkmenistan	1649.2<br><br>(1581.9 to 1714.9)	51.5<br><br>(49.6 to 53.4)	2921.6<br><br>(2807.5 to 3042.9)	56.8<br><br>(54.6 to 59.2)	0.32<br><br>(0.32 to 0.33)
Tuvalu	4.2<br><br>(4.1 to 4.4)	48.9<br><br>(47.0 to 50.8)	6.9<br><br>(6.6 to 7.1)	57.5<br><br>(55.3 to 59.7)	0.53<br><br>(0.53 to 0.53)

Uganda	2940.2 (2823.0 to 3053.3)	21.3 (20.5 to 22.1)	8040.1 (7724.9 to 8337.8)	22.7 (21.9 to 23.4)	0.2 (0.2 to 0.21)
Ukraine	30678.3 (29550.9 to 31807.6)	53.2 (51.3 to 55.0)	29041.9 (28088.8 to 30025.0)	57.7 (56.0 to 59.7)	0.27 (0.26 to 0.27)
United Arab Emirates	1053.3 (1009.7 to 1106.5)	60.2 (58.0 to 62.4)	8285.7 (7803.5 to 8830.0)	72.8 (70.0 to 76.0)	0.61 (0.61 to 0.61)
United Kingdom	58226.7 (56537.3 to 60077.4)	95.0 (92.1 to 98.0)	81325.7 (78879.1 to 83986.6)	108.1 (104.8 to 111.5)	0.39 (0.37 to 0.41)
United Republic of Tanzania	4825.4 (4574.3 to 5062.6)	22.8 (21.7 to 23.8)	11789.9 (11217.7 to 12334.4)	23.5 (22.5 to 24.7)	0.11 (0.1 to 0.11)
United States of America	254425.2 (247149.2 to 262221.5)	97.4 (94.6 to 100.4)	380893.2 (370220.6 to 391421.1)	104.0 (101.4 to 106.6)	0.21 (0.19 to 0.23)
United States Virgin Islands	85.4 (82.0 to 88.7)	80.7 (77.6 to 83.9)	85.0 (81.4 to 88.9)	86.3 (82.8 to 89.7)	0.21 (0.21 to 0.22)
Uruguay	2204.6 (2126.6 to 2284.1)	67.9 (65.4 to 70.4)	2809.7 (2698.8 to 2921.0)	75.9 (72.8 to 78.7)	0.34 (0.33 to 0.36)
Uzbekistan	8887.1 (8521.0 to 9232.4)	49.2 (47.3 to 51.2)	18926.2 (18192.8 to 19705.3)	55.4 (53.2 to 57.7)	0.39 (0.38 to 0.4)
Vanuatu	56.5 (54.3 to 58.7)	46.6 (45.0 to 48.4)	145.0 (139.0 to 150.8)	52.4 (50.4 to 54.6)	0.38 (0.38 to 0.39)
Venezuela (Bolivarian Republic of)	12756.8 (12206.3 to 13283.8)	75.2 (72.1 to 78.3)	22138.7 (21224.3 to 23008.1)	78.9 (75.4 to 82.0)	0.15 (0.15 to 0.16)

Viet Nam	32265.7<br><br>(31026.9 to 33579.3)	55.5<br><br>(53.4 to 57.7)	70452.3<br><br>(67528.1 to 73477.0)	66.3<br><br>(63.7 to 69.0)	0.58<br><br>(0.57 to 0.58)
Yemen	5036.2<br><br>(4835.6 to 5214.7)	48.4<br><br>(46.6 to 50.1)	17119.3<br><br>(16460.8 to 17791.5)	59.1<br><br>(56.7 to 61.5)	0.65<br><br>(0.64 to 0.65)
Zambia	1429.1<br><br>(1370.4 to 1483.8)	22.3<br><br>(21.6 to 23.1)	3831.5<br><br>(3676.3 to 3969.2)	23.3<br><br>(22.4 to 24.1)	0.13<br><br>(0.12 to 0.13)
Zimbabwe	2630.7<br><br>(2508.7 to 2744.9)	30.2<br><br>(29.0 to 31.4)	4310.1<br><br>(4125.8 to 4477.6)	31.1<br><br>(29.7 to 32.2)	0.09<br><br>(0.08 to 0.1)

Abbreviations: ASR: age-standardized rate; AAPC: average annual percentage change; CI: confidence interval; UI: uncertainty interval.

Supplement table 5. Age-standardized DALY rates and AAPC for psoriasis at the national level from 1990 to 2021.

location	1990		2021		AAPC (95%CI)
	Number (95%UI)	ASR (95%UI)	Numbe (95%UI)	ASR (95%UI)	
Afghanistan	2237.6<br><br>(1559.2 to 3004.7)	27.6<br><br>(19.3 to 37.0)	7468.7<br><br>(5341.2 to 9989.1)	31.7<br><br>(22.8 to 42.1)	0.46<br><br>(0.44 to 0.47)
Albania	968.9<br><br>(700.2 to 1292.4)	32.6<br><br>(23.5 to 43.2)	1222.4<br><br>(860.8 to 1624.6)	38.3<br><br>(27.0 to 51.3)	0.52<br><br>(0.51 to 0.54)
Algeria	7181.8<br><br>(5067.6 to 9691.2)	36.0<br><br>(25.8 to 48.1)	19104.0<br><br>(13832.1 to 25649.5)	43.5<br><br>(31.5 to 58.3)	0.61<br><br>(0.6 to 0.62)
American Samoa	14.0<br><br>(9.9 to 18.7)	36.4<br><br>(25.8 to 48.4)	20.7<br><br>(15.0 to 27.8)	40.2<br><br>(29.4 to 53.9)	0.32<br><br>(0.31 to 0.33)
Andorra	62.8<br><br>(45.7 to 84.6)	103.8<br><br>(75.4 to	123.6<br><br>(89.0 to	110.9<br><br>(79.9 to	0.21<br><br>(0.21 to

		140.0)	164.0)	148.2)	0.22)
Angola	1771.5 (1269.2 to 2418.4)	21.4 (15.5 to 28.8)	7097.1 (5079.1 to 9524.2)	26.9 (19.5 to 36.0)	0.74 (0.73 to 0.75)
Antigua and Barbuda	31.4 (22.5 to 41.9)	55.0 (39.4 to 72.9)	60.4 (43.7 to 79.9)	60.6 (43.9 to 80.4)	0.32 (0.31 to 0.33)
Argentina	15214.8 (11051.4 to 20206.8)	46.6 (33.8 to 61.9)	25666.5 (18671.5 to 34176.5)	52.3 (38.0 to 69.5)	0.37 (0.36 to 0.38)
Armenia	1034.1 (740.7 to 1372.6)	31.5 (22.7 to 41.6)	1281.4 (910.4 to 1696.6)	37.0 (26.4 to 49.3)	0.53 (0.52 to 0.54)
Australia	8190.6 (5918.8 to 10862.2)	45.5 (32.8 to 60.8)	15780.7 (11267.4 to 20907.4)	52.5 (37.4 to 69.9)	0.46 (0.44 to 0.47)
Austria	8436.8 (6114.9 to 10993.4)	94.4 (68.3 to 123.3)	11767.2 (8442.9 to 15578.5)	105.1 (75.7 to 140.0)	0.34 (0.33 to 0.34)
Azerbaijan	2198.1 (1593.9 to 2976.7)	33.0 (24.1 to 44.6)	4253.5 (3061.3 to 5653.1)	37.0 (26.6 to 49.5)	0.38 (0.37 to 0.39)
Bahamas	138.5 (100.1 to 185.9)	59.1 (42.6 to 79.0)	262.1 (190.4 to 350.7)	62.2 (45.3 to 83.5)	0.17 (0.16 to 0.18)
Bahrain	177.6 (128.0 to 237.8)	39.6 (28.8 to 52.6)	791.2 (567.0 to 1072.3)	47.3 (34.0 to 64.1)	0.58 (0.56 to 0.59)
Bangladesh	23274.6 (16553.6 to 31179.4)	26.1 (18.7 to 34.3)	50091.5 (35706.4 to 67898.9)	30.5 (21.7 to 41.3)	0.5 (0.48 to 0.52)

Barbados	152.3 (109.3 to 204.8)	59.2 (42.6 to 80.0)	219.2 (159.4 to 285.9)	61.4 (44.7 to 80.5)	0.12 (0.11 to 0.12)
Belarus	3693.5 (2610.3 to 4963.7)	32.3 (22.8 to 43.8)	4275.1 (3050.8 to 5702.1)	37.5 (27.0 to 50.8)	0.48 (0.47 to 0.49)
Belgium	10754.1 (7734.5 to 14252.5)	93.0 (67.1 to 123.3)	14593.7 (10653.5 to 19418.9)	104.3 (75.6 to 139.4)	0.36 (0.36 to 0.37)
Belize	72.6 (51.2 to 95.5)	47.7 (34.0 to 62.5)	221.4 (160.1 to 297.2)	53.8 (38.9 to 72.1)	0.39 (0.38 to 0.41)
Benin	610.6 (447.7 to 821.2)	16.2 (11.9 to 21.6)	1911.9 (1362.5 to 2523.4)	17.4 (12.6 to 23.0)	0.25 (0.23 to 0.26)
Bermuda	36.3 (26.1 to 48.3)	57.3 (41.1 to 76.0)	49.2 (35.9 to 65.0)	61.7 (45.1 to 82.3)	0.24 (0.23 to 0.24)
Bhutan	129.4 (92.7 to 173.4)	24.7 (17.8 to 33.2)	225.8 (161.2 to 302.5)	29.6 (21.1 to 39.6)	0.58 (0.57 to 0.59)
Bolivia (Plurinational State of)	3602.4 (2613.1 to 4787.3)	67.1 (48.8 to 89.1)	9255.2 (6662.6 to 12310.0)	80.9 (58.5 to 107.4)	0.61 (0.61 to 0.62)
Bosnia and Herzegovina	1465.8 (1065.6 to 1947.7)	31.2 (22.6 to 41.5)	1600.2 (1169.8 to 2116.3)	38.8 (28.1 to 51.7)	0.72 (0.7 to 0.74)
Botswana	199.6 (140.2 to 271.6)	18.6 (13.1 to 25.1)	486.6 (353.8 to 655.2)	21.1 (15.3 to 28.2)	0.4 (0.39 to 0.42)
Brazil	48024.3 (34529.9 to 64094.2)	35.6 (25.7 to 47.5)	82785.0 (60113.3 to 109845.5)	35.2 (25.5 to 46.7)	-0.04 (-0.05 to -0.03)

Brunei Darussalam	74.0<br><br>(53.4 to 100.1)	33.7<br><br>(24.4 to 45.0)	173.5<br><br>(124.0 to 233.7)	36.4<br><br>(26.0 to 49.2)	0.24<br><br>(0.22 to 0.25)
Bulgaria	3530.9<br><br>(2565.9 to 4749.0)	34.9<br><br>(25.1 to 46.8)	3472.8<br><br>(2459.9 to 4650.5)	40.6<br><br>(28.6 to 54.7)	0.48<br><br>(0.47 to 0.49)
Burkina Faso	1134.7<br><br>(806.3 to 1520.4)	15.0<br><br>(10.7 to 20.2)	3012.8<br><br>(2101.5 to 4130.7)	16.3<br><br>(11.6 to 22.2)	0.28<br><br>(0.26 to 0.29)
Burundi	530.1<br><br>(373.4 to 709.0)	12.2<br><br>(8.7 to 16.3)	1243.3<br><br>(878.7 to 1647.6)	11.6<br><br>(8.2 to 15.4)	-0.17<br><br>(-0.19 to -0.15)
Cabo Verde	49.8<br><br>(35.8 to 67.0)	17.1<br><br>(12.2 to 22.9)	109.4<br><br>(76.4 to 144.1)	19.5<br><br>(13.7 to 25.7)	0.44<br><br>(0.42 to 0.45)
Cambodia	2553.8<br><br>(1827.9 to 3475.5)	32.9<br><br>(23.7 to 44.5)	6467.4<br><br>(4602.4 to 8484.1)	40.4<br><br>(28.8 to 53.1)	0.67<br><br>(0.65 to 0.68)
Cameroon	1481.1<br><br>(1066.1 to 1956.4)	17.7<br><br>(12.7 to 23.3)	5135.0<br><br>(3630.4 to 6954.3)	19.4<br><br>(13.9 to 26.0)	0.27<br><br>(0.24 to 0.3)
Canada	20709.3<br><br>(14894.3 to 27645.1)	70.4<br><br>(50.4 to 94.6)	35411.7<br><br>(25797.0 to 46949.0)	78.8<br><br>(57.5 to 105.5)	0.36<br><br>(0.35 to 0.37)
Central African Republic	469.0<br><br>(329.5 to 629.3)	21.0<br><br>(14.9 to 28.0)	1076.4<br><br>(761.2 to 1461.0)	23.1<br><br>(16.5 to 31.2)	0.32<br><br>(0.31 to 0.33)
Chad	705.3<br><br>(500.1 to 962.9)	14.8<br><br>(10.6 to 19.9)	2102.0<br><br>(1495.3 to 2849.2)	15.5<br><br>(11.0 to 20.8)	0.15<br><br>(0.12 to 0.17)
Chile	5734.3<br><br>(4071.5 to 7654.7)	45.7<br><br>(32.5 to 60.8)	11381.3<br><br>(8274.8 to 15241.6)	53.6<br><br>(38.8 to 71.9)	0.52<br><br>(0.51 to 0.53)

China	343657.0<br><br>(247482.6 to 461827.6)	31.4<br><br>(22.7 to 42.1)	728553.2<br><br>(528686.2 to 971655.4)	41.2<br><br>(29.8 to 55.1)	0.88<br><br>(0.88 to 0.89)
Colombia	14959.6<br><br>(10560.7 to 19822.2)	52.4<br><br>(37.3 to 68.8)	30455.8<br><br>(21803.2 to 40529.7)	58.5<br><br>(41.7 to 78.0)	0.36<br><br>(0.35 to 0.37)
Comoros	46.5<br><br>(33.0 to 62.0)	12.7<br><br>(9.1 to 16.5)	91.3<br><br>(64.1 to 122.6)	13.2<br><br>(9.2 to 17.7)	0.1<br><br>(0.09 to 0.12)
Congo	501.9<br><br>(355.4 to 677.5)	25.4<br><br>(18.4 to 33.6)	1486.4<br><br>(1073.9 to 1989.4)	30.4<br><br>(22.0 to 40.9)	0.58<br><br>(0.57 to 0.59)
Cook Islands	5.9<br><br>(4.3 to 7.8)	35.6<br><br>(26.0 to 47.1)	8.5<br><br>(6.2 to 11.2)	41.3<br><br>(30.2 to 54.6)	0.48<br><br>(0.47 to 0.5)
Costa Rica	1441.1<br><br>(1027.8 to 1905.7)	54.2<br><br>(38.8 to 71.7)	3044.9<br><br>(2165.9 to 4094.0)	59.6<br><br>(42.3 to 80.5)	0.3<br><br>(0.29 to 0.31)
Coted'Ivoire	1619.0<br><br>(1136.1 to 2214.3)	16.7<br><br>(12.0 to 22.7)	4312.1<br><br>(3099.2 to 5804.4)	18.3<br><br>(13.3 to 24.5)	0.3<br><br>(0.28 to 0.32)
Croatia	1985.9<br><br>(1390.8 to 2661.6)	35.8<br><br>(25.1 to 48.5)	2179.1<br><br>(1600.5 to 2876.8)	41.3<br><br>(30.3 to 55.3)	0.48<br><br>(0.46 to 0.49)
Cuba	5859.0<br><br>(4196.7 to 7799.3)	53.7<br><br>(38.4 to 71.8)	7540.5<br><br>(5397.5 to 10044.2)	55.9<br><br>(40.3 to 74.4)	0.13<br><br>(0.12 to 0.14)
Cyprus	744.4<br><br>(528.7 to 992.2)	91.8<br><br>(65.0 to 122.3)	1772.7<br><br>(1282.4 to 2372.1)	108.5<br><br>(77.8 to 145.6)	0.54<br><br>(0.54 to 0.55)
Czechia	4089.0<br><br>(2939.2 to 5472.8)	35.5<br><br>(25.7 to 47.6)	5416.5<br><br>(3835.8 to 7137.5)	41.4<br><br>(29.5 to 54.9)	0.49<br><br>(0.48 to 0.51)

Democratic People's Republic of Korea	3976.4 (2861.7 to 5436.8)	20.0 (14.5 to 27.1)	7377.8 (5238.4 to 9818.3)	24.1 (17.2 to 32.0)	0.6 (0.59 to 0.61)
Democratic Republic of the Congo	6897.7 (4931.3 to 9210.0)	22.5 (16.1 to 29.9)	19093.8 (13569.0 to 26119.3)	25.2 (18.2 to 34.3)	0.37 (0.35 to 0.38)
Denmark	5730.2 (4109.8 to 7582.1)	96.0 (68.9 to 127.2)	7593.7 (5524.7 to 10020.0)	106.1 (76.5 to 141.0)	0.32 (0.31 to 0.32)
Djibouti	41.9 (29.2 to 55.8)	12.5 (8.8 to 16.7)	148.3 (103.2 to 204.9)	12.8 (8.9 to 17.6)	0.08 (0.06 to 0.09)
Dominica	35.9 (25.7 to 48.2)	54.0 (38.8 to 72.6)	44.8 (32.3 to 59.1)	60.3 (43.3 to 79.8)	0.36 (0.35 to 0.37)
Dominican Republic	2889.2 (2063.4 to 3895.9)	47.2 (33.8 to 63.6)	5804.8 (4150.8 to 7680.5)	52.9 (37.8 to 69.8)	0.37 (0.37 to 0.38)
Ecuador	6681.3 (4733.3 to 9090.5)	77.7 (55.1 to 104.9)	16308.5 (11688.8 to 21803.1)	90.9 (65.2 to 121.6)	0.51 (0.5 to 0.52)
Egypt	9394.5 (6692.1 to 12877.4)	20.3 (14.5 to 27.9)	24034.8 (17027.1 to 32801.1)	24.8 (17.7 to 33.7)	0.65 (0.64 to 0.67)
El Salvador	2085.0 (1492.1 to 2791.5)	46.3 (33.3 to 61.7)	3371.3 (2421.9 to 4497.9)	53.0 (38.1 to 70.6)	0.44 (0.43 to 0.45)
Equatorial Guinea	74.7 (52.4 to 100.6)	21.8 (15.3 to 29.2)	421.4 (296.8 to 562.9)	33.0 (23.7 to 44.3)	1.35 (1.33 to 1.36)
Eritrea	315.9 (222.4 to 420.3)	11.8 (8.5 to 15.8)	697.8 (506.1 to 947.5)	12.3 (8.9 to 16.6)	0.15 (0.14 to 0.16)

Estonia	567.5 (401.8 to 754.1)	32.7 (23.3 to 43.6)	616.6 (441.6 to 820.3)	38.9 (27.8 to 51.6)	0.58 (0.56 to 0.59)
Eswatini	113.7 (80.3 to 151.8)	18.0 (12.9 to 23.9)	204.3 (147.4 to 275.8)	19.8 (14.3 to 26.6)	0.31 (0.29 to 0.32)
Ethiopia	5353.1 (3832.6 to 7160.0)	13.6 (9.8 to 18.3)	12732.8 (9048.7 to 17131.9)	14.3 (10.3 to 19.2)	0.15 (0.14 to 0.16)
Fiji	204.7 (145.5 to 274.4)	32.6 (23.3 to 43.3)	343.0 (250.6 to 450.5)	37.6 (27.4 to 49.4)	0.47 (0.46 to 0.48)
Finland	5173.0 (3703.9 to 6889.4)	90.0 (65.0 to 120.4)	6894.3 (4964.0 to 9214.5)	100.1 (71.8 to 134.1)	0.34 (0.34 to 0.35)
France	59402.8 (43460.1 to 79702.4)	92.2 (67.1 to 124.0)	83972.4 (60293.6 to 111387.0)	104.6 (74.4 to 139.4)	0.4 (0.39 to 0.41)
Gabon	217.8 (152.9 to 294.0)	25.9 (18.6 to 34.6)	536.8 (380.3 to 721.7)	32.4 (23.2 to 42.9)	0.72 (0.7 to 0.73)
Gambia	126.2 (89.8 to 167.3)	16.3 (11.5 to 21.6)	357.8 (241.6 to 478.1)	17.8 (12.3 to 23.6)	0.28 (0.26 to 0.31)
Georgia	2073.5 (1476.2 to 2740.7)	35.2 (25.1 to 46.4)	1572.3 (1139.4 to 2114.0)	37.4 (26.9 to 51.1)	0.2 (0.18 to 0.21)
Germany	116239.9 (83934.1 to 155490.4)	122.5 (88.8 to 164.1)	149394.6 (107820.0 to 198715.1)	137.4 (99.5 to 185.6)	0.36 (0.34 to 0.38)
Ghana	2018.8 (1437.7 to 2734.0)	16.5 (11.9 to 22.2)	5535.9 (3925.7 to 7457.8)	18.3 (13.2 to 24.9)	0.34 (0.32 to 0.36)

Greece	10997.0 (7952.6 to 14844.8)	92.0 (66.5 to 124.0)	13360.9 (9806.1 to 17767.0)	103.2 (75.1 to 138.0)	0.37 (0.37 to 0.38)
Greenland	34.5 (24.9 to 46.0)	64.1 (46.4 to 85.6)	46.2 (33.6 to 62.1)	73.0 (53.3 to 98.5)	0.42 (0.41 to 0.43)
Grenada	38.3 (27.8 to 51.5)	49.7 (36.2 to 66.5)	64.0 (46.8 to 84.9)	57.6 (42.1 to 77.0)	0.48 (0.47 to 0.48)
Guam	46.7 (33.4 to 62.9)	38.9 (28.0 to 51.8)	78.4 (56.4 to 105.0)	44.1 (31.8 to 59.0)	0.41 (0.4 to 0.43)
Guatemala	2838.1 (2044.5 to 3919.9)	42.6 (30.9 to 58.1)	7358.0 (5270.2 to 9949.3)	49.8 (36.0 to 66.9)	0.5 (0.5 to 0.51)
Guinea	781.6 (565.8 to 1058.5)	15.8 (11.4 to 21.1)	1876.8 (1358.2 to 2540.9)	17.1 (12.3 to 22.7)	0.24 (0.22 to 0.25)
Guinea-Bissau	128.8 (90.5 to 175.6)	16.1 (11.5 to 21.8)	293.9 (210.2 to 396.4)	17.3 (12.4 to 23.1)	0.23 (0.21 to 0.25)
Guyana	330.1 (240.6 to 441.6)	49.8 (36.5 to 66.5)	429.0 (307.9 to 580.1)	56.6 (40.6 to 76.5)	0.42 (0.4 to 0.43)
Haiti	2218.5 (1578.0 to 2950.9)	41.4 (29.5 to 55.1)	5238.7 (3730.3 to 7066.0)	44.7 (31.9 to 60.2)	0.25 (0.24 to 0.25)
Honduras	1655.8 (1166.0 to 2208.0)	44.0 (31.2 to 58.9)	4611.0 (3290.8 to 6168.6)	49.5 (35.5 to 65.8)	0.39 (0.37 to 0.4)
Hungary	4144.9 (2915.8 to 5528.1)	34.8 (24.4 to 46.5)	4904.1 (3500.7 to 6446.6)	41.1 (29.1 to 54.1)	0.53 (0.52 to 0.54)

Iceland	238.5 (173.7 to 321.8)	91.2 (66.5 to 123.2)	412.8 (299.2 to 549.0)	101.2 (73.3 to 135.1)	0.34 (0.33 to 0.35)
India	222182.4 (160989.2 to 299581.7)	29.2 (21.2 to 39.3)	449733.6 (325102.9 to 604120.7)	31.4 (22.7 to 42.1)	0.19 (0.12 to 0.25)
Indonesia	62049.9 (44844.2 to 83189.3)	39.8 (28.7 to 53.1)	140507.9 (100754.2 to 187693.9)	48.6 (35.0 to 64.8)	0.64 (0.63 to 0.65)
Iran (Islamic Republic of)	15183.1 (11022.2 to 20371.0)	34.1 (24.8 to 45.6)	39783.7 (28894.4 to 53304.1)	43.3 (31.4 to 57.8)	0.77 (0.77 to 0.78)
Iraq	5941.8 (4185.1 to 7998.1)	43.1 (30.7 to 57.3)	20986.4 (15357.8 to 27638.9)	55.7 (40.6 to 73.4)	0.83 (0.82 to 0.83)
Ireland	3335.6 (2401.7 to 4428.4)	90.7 (65.2 to 120.5)	6042.9 (4392.6 to 8064.5)	104.6 (75.9 to 140.4)	0.46 (0.46 to 0.47)
Israel	4642.3 (3275.6 to 6239.1)	97.0 (68.4 to 130.4)	10588.1 (7553.5 to 14264.0)	106.3 (75.9 to 143.4)	0.3 (0.29 to 0.3)
Italy	48946.8 (35601.4 to 65274.1)	73.5 (53.2 to 98.3)	60658.0 (44118.0 to 80699.2)	78.7 (56.9 to 105.3)	0.22 (0.21 to 0.23)
Jamaica	1111.1 (799.7 to 1508.0)	52.3 (37.7 to 70.8)	1696.8 (1210.4 to 2301.7)	57.2 (40.6 to 77.8)	0.29 (0.28 to 0.29)
Japan	48318.6 (35046.1 to 64456.4)	33.4 (24.1 to 44.6)	56240.2 (40725.6 to 75023.1)	34.2 (24.7 to 45.7)	0.07 (0.07 to 0.08)
Jordan	1052.1 (744.1 to 1407.6)	37.8 (27.2 to 49.9)	5259.1 (3841.3 to 6996.2)	44.8 (32.7 to 59.3)	0.56 (0.55 to 0.57)

Kazakhstan	5056.5 (3570.9 to 6685.2)	32.7 (23.0 to 43.1)	7198.4 (5251.2 to 9557.4)	37.1 (27.1 to 49.2)	0.42 (0.4 to 0.43)
Kenya	2402.0 (1728.5 to 3215.5)	13.5 (9.8 to 18.1)	6044.4 (4369.4 to 8130.3)	14.0 (10.2 to 18.8)	0.09 (0.08 to 0.1)
Kiribati	18.4 (13.0 to 24.7)	30.9 (22.2 to 41.0)	37.6 (26.5 to 50.9)	35.2 (24.9 to 47.3)	0.42 (0.41 to 0.44)
Kuwait	652.9 (466.7 to 867.3)	42.9 (30.6 to 57.0)	2657.5 (1884.0 to 3559.3)	50.7 (36.2 to 67.7)	0.55 (0.54 to 0.56)
Kyrgyzstan	1240.8 (899.3 to 1665.6)	32.0 (23.4 to 42.9)	2223.7 (1572.5 to 2960.9)	34.5 (24.5 to 46.1)	0.25 (0.23 to 0.26)
Lao People's Democratic Republic	1057.3 (758.0 to 1435.3)	32.5 (23.4 to 43.7)	2732.6 (1969.1 to 3688.4)	40.9 (29.6 to 55.0)	0.75 (0.73 to 0.76)
Latvia	985.3 (716.3 to 1318.3)	33.2 (24.1 to 44.6)	890.5 (635.8 to 1185.7)	38.8 (27.8 to 51.9)	0.51 (0.49 to 0.52)
Lebanon	961.3 (691.1 to 1286.1)	35.5 (25.7 to 47.8)	2570.6 (1864.2 to 3421.1)	43.7 (31.8 to 58.2)	0.68 (0.66 to 0.69)
Lesotho	217.3 (153.6 to 289.7)	16.9 (12.1 to 22.3)	313.7 (222.4 to 421.7)	18.3 (13.1 to 24.4)	0.27 (0.25 to 0.28)
Liberia	323.0 (228.7 to 434.8)	16.2 (11.4 to 21.5)	816.3 (570.1 to 1098.6)	17.4 (12.4 to 23.0)	0.23 (0.21 to 0.25)
Libya	1187.6 (842.5 to 1587.5)	36.3 (25.8 to 48.1)	3288.3 (2368.4 to 4457.7)	45.0 (32.5 to 60.4)	0.69 (0.67 to 0.69)

Lithuania	1322.3 (957.4 to 1737.3)	33.1 (23.8 to 43.5)	1347.9 (985.5 to 1817.1)	40.0 (29.1 to 54.2)	0.62 (0.6 to 0.63)
Luxembourg	427.9 (306.3 to 567.0)	96.6 (69.1 to 128.6)	842.2 (607.0 to 1121.3)	107.4 (77.9 to 143.7)	0.34 (0.34 to 0.35)
Madagascar	1150.4 (819.3 to 1601.7)	12.2 (8.7 to 17.1)	2865.8 (1988.5 to 3826.8)	12.0 (8.2 to 15.9)	-0.03 (-0.04 to -0.02)
Malawi	914.3 (656.3 to 1212.2)	11.9 (8.6 to 15.9)	1940.5 (1367.9 to 2557.7)	12.2 (8.7 to 16.1)	0.06 (0.04 to 0.08)
Malaysia	6480.1 (4605.1 to 8560.2)	44.2 (31.8 to 58.5)	17082.0 (12311.8 to 22642.2)	52.8 (38.0 to 69.7)	0.57 (0.56 to 0.59)
Maldives	59.3 (42.6 to 79.9)	36.4 (26.2 to 48.3)	256.7 (183.0 to 345.9)	48.6 (35.1 to 64.9)	0.94 (0.92 to 0.95)
Mali	1111.0 (773.5 to 1465.0)	16.0 (11.3 to 21.1)	3323.0 (2346.5 to 4487.7)	17.5 (12.6 to 23.3)	0.3 (0.28 to 0.32)
Malta	367.2 (265.5 to 483.6)	91.5 (66.3 to 120.6)	586.7 (420.1 to 781.5)	105.1 (75.5 to 140.0)	0.44 (0.44 to 0.45)
Marshall Islands	9.9 (7.1 to 13.4)	30.8 (22.4 to 40.5)	18.3 (13.0 to 24.2)	35.1 (25.3 to 46.3)	0.43 (0.42 to 0.44)
Mauritania	288.7 (202.5 to 391.1)	17.2 (12.2 to 22.8)	705.9 (498.6 to 945.4)	18.9 (13.6 to 25.2)	0.3 (0.27 to 0.32)
Mauritius	413.0 (296.8 to 546.6)	41.4 (29.8 to 54.9)	746.9 (536.6 to 985.2)	48.8 (35.1 to 64.9)	0.53 (0.51 to 0.54)

Mexico	36565.9 (26417.7 to 48806.0)	50.2 (36.5 to 66.7)	74181.5 (54036.2 to 99119.4)	55.4 (40.4 to 74.0)	0.32 (0.31 to 0.32)
Micronesia (Federated States of)	25.0 (17.8 to 33.9)	31.8 (22.7 to 42.7)	35.6 (25.4 to 47.0)	36.6 (26.3 to 48.2)	0.45 (0.44 to 0.46)
Monaco	40.9 (29.7 to 54.5)	103.9 (74.8 to 138.5)	55.6 (40.7 to 74.0)	111.5 (81.5 to 148.5)	0.22 (0.22 to 0.23)
Mongolia	499.5 (355.1 to 664.9)	28.8 (20.6 to 38.1)	1065.5 (773.1 to 1425.9)	33.5 (24.5 to 44.7)	0.49 (0.47 to 0.5)
Montenegro	235.6 (170.4 to 315.4)	36.4 (26.3 to 48.7)	304.3 (219.8 to 401.6)	41.2 (29.9 to 54.9)	0.42 (0.41 to 0.43)
Morocco	6903.6 (4924.4 to 9269.4)	32.6 (23.5 to 43.5)	15008.5 (10838.8 to 20036.0)	39.2 (28.4 to 52.3)	0.6 (0.59 to 0.61)
Mozambique	1191.5 (833.7 to 1602.9)	11.0 (7.8 to 14.7)	2810.5 (1988.4 to 3774.0)	11.3 (8.1 to 15.0)	0.08 (0.06 to 0.1)
Myanmar	11167.6 (8019.8 to 14907.3)	32.7 (23.6 to 43.5)	22984.5 (16523.6 to 30710.9)	41.0 (29.6 to 54.8)	0.74 (0.73 to 0.76)
Namibia	221.2 (156.3 to 297.6)	19.1 (13.4 to 25.5)	462.6 (333.5 to 639.5)	20.8 (15.1 to 28.2)	0.26 (0.24 to 0.28)
Nauru	2.8 (2.0 to 3.7)	34.4 (24.6 to 45.6)	3.6 (2.6 to 4.9)	38.7 (27.8 to 52.4)	0.38 (0.36 to 0.4)
Nepal	3710.8 (2636.3 to 5043.5)	22.5 (16.1 to 30.2)	8814.7 (6439.0 to 11693.7)	29.3 (21.3 to 39.1)	0.84 (0.81 to 0.87)

Netherlands	16283.0 (11706.9 to 21538.8)	97.0 (69.9 to 128.6)	22795.0 (16554.3 to 29959.4)	106.6 (77.5 to 141.7)	0.3 (0.29 to 0.31)
New Zealand	1582.0 (1141.4 to 2116.5)	44.1 (31.8 to 58.9)	2977.3 (2157.8 to 3944.2)	50.0 (36.2 to 66.7)	0.42 (0.41 to 0.43)
Nicaragua	1366.5 (969.7 to 1849.8)	44.5 (31.7 to 59.5)	3237.7 (2331.1 to 4339.1)	50.6 (36.4 to 67.6)	0.42 (0.41 to 0.43)
Niger	886.5 (618.6 to 1219.2)	14.3 (10.0 to 19.5)	2776.3 (1979.3 to 3827.4)	14.5 (10.4 to 19.7)	0.03 (0.02 to 0.05)
Nigeria	28537.0 (20769.9 to 38215.7)	38.8 (28.1 to 51.5)	88465.1 (64175.5 to 118564.3)	47.4 (34.5 to 63.3)	0.65 (0.64 to 0.65)
Niue	0.7 (0.5 to 1.0)	33.5 (24.7 to 45.3)	0.7 (0.5 to 1.0)	39.5 (29.0 to 52.9)	0.53 (0.52 to 0.53)
North Macedonia	706.1 (503.9 to 941.1)	34.5 (24.6 to 45.9)	1069.0 (775.7 to 1431.3)	40.1 (29.1 to 54.2)	0.5 (0.49 to 0.52)
Northern Mariana Islands	15.9 (11.5 to 20.9)	40.3 (29.1 to 52.7)	23.6 (16.8 to 30.8)	43.0 (30.8 to 56.6)	0.21 (0.19 to 0.23)
Norway	3239.5 (2348.7 to 4322.9)	67.7 (49.0 to 90.4)	4627.7 (3338.7 to 6206.0)	71.5 (51.6 to 96.1)	0.18 (0.17 to 0.18)
Oman	572.3 (414.2 to 789.8)	36.4 (26.2 to 49.5)	2309.4 (1632.0 to 3124.0)	49.1 (35.0 to 65.8)	0.97 (0.95 to 0.98)
Pakistan	23502.2 (16560.9 to 31350.0)	25.4 (18.0 to 33.5)	61673.3 (44495.3 to 82446.5)	29.0 (21.1 to 38.5)	0.43 (0.43 to 0.44)

Palau	5.1 (3.6 to 6.8)	37.1 (26.6 to 49.9)	9.3 (6.7 to 12.5)	42.2 (30.5 to 56.4)	0.41 (0.41 to 0.42)
Palestine	470.2 (332.0 to 635.8)	31.5 (22.6 to 42.3)	1750.4 (1257.5 to 2345.5)	39.4 (28.6 to 53.2)	0.72 (0.71 to 0.73)
Panama	1173.5 (833.0 to 1547.7)	54.9 (39.1 to 72.3)	2632.4 (1916.2 to 3489.1)	60.3 (43.8 to 79.8)	0.31 (0.3 to 0.32)
Papua New Guinea	883.9 (630.4 to 1161.6)	27.6 (19.8 to 36.2)	2773.4 (1946.5 to 3696.7)	31.8 (22.7 to 41.7)	0.46 (0.45 to 0.47)
Paraguay	1203.8 (868.1 to 1614.7)	34.8 (25.2 to 46.3)	2506.1 (1795.9 to 3329.7)	35.7 (25.6 to 47.4)	0.08 (0.07 to 0.09)
Peru	14067.3 (10170.5 to 19026.2)	75.1 (54.1 to 100.5)	32589.6 (23619.2 to 43636.4)	89.5 (64.9 to 119.7)	0.56 (0.55 to 0.58)
Philippines	20282.6 (14572.5 to 27014.5)	40.4 (29.2 to 53.8)	48890.6 (35465.4 to 65625.6)	45.9 (33.3 to 61.4)	0.42 (0.42 to 0.43)
Poland	15921.5 (11473.5 to 21145.3)	39.1 (28.2 to 52.0)	20639.9 (15172.0 to 27289.7)	43.9 (32.1 to 58.2)	0.37 (0.37 to 0.38)
Portugal	9703.4 (6953.2 to 13021.0)	85.3 (60.9 to 114.4)	13402.0 (9648.7 to 17637.5)	98.5 (70.4 to 130.2)	0.46 (0.45 to 0.47)
Puerto Rico	2140.9 (1545.4 to 2851.1)	58.9 (42.5 to 78.3)	2610.8 (1892.5 to 3438.5)	65.1 (47.4 to 86.9)	0.33 (0.32 to 0.34)
Qatar	171.1 (121.7 to 232.3)	41.2 (29.7 to 55.5)	1681.4 (1168.2 to 2293.9)	51.1 (36.5 to 69.0)	0.69 (0.68 to 0.7)

Republic of Korea	13087.4 (9204.3 to 17600.9)	30.3 (21.5 to 40.5)	23037.1 (16603.5 to 30579.9)	34.8 (25.1 to 46.1)	0.45 (0.44 to 0.46)
Republic of Moldova	1430.1 (1035.0 to 1939.8)	31.4 (22.7 to 42.6)	1608.4 (1134.5 to 2159.9)	36.4 (25.7 to 48.6)	0.49 (0.47 to 0.5)
Romania	8581.3 (6262.1 to 11323.2)	33.6 (24.4 to 44.7)	8911.2 (6433.9 to 11850.5)	38.2 (27.6 to 51.1)	0.42 (0.41 to 0.43)
Russian Federation	55128.6 (40054.3 to 73275.4)	33.4 (24.3 to 44.5)	66647.6 (48786.5 to 88847.5)	38.2 (27.7 to 50.9)	0.44 (0.43 to 0.44)
Rwanda	550.3 (383.4 to 724.6)	9.8 (6.9 to 13.0)	1168.4 (823.1 to 1599.5)	10.2 (7.2 to 13.8)	0.1 (0.07 to 0.12)
Saint Kitts and Nevis	20.9 (15.2 to 28.2)	54.6 (39.2 to 73.4)	40.8 (29.1 to 54.3)	60.9 (43.5 to 81.2)	0.35 (0.34 to 0.36)
Saint Lucia	60.0 (42.9 to 79.9)	50.2 (36.0 to 66.6)	115.6 (83.7 to 153.5)	56.9 (41.3 to 76.0)	0.4 (0.39 to 0.41)
Saint Vincent and the Grenadines	47.6 (34.5 to 63.1)	49.5 (35.8 to 64.9)	68.9 (49.8 to 90.7)	55.0 (39.8 to 72.5)	0.34 (0.33 to 0.35)
Samoa	43.7 (31.3 to 58.2)	33.3 (23.9 to 44.4)	70.0 (50.5 to 93.8)	37.7 (27.3 to 50.2)	0.41 (0.4 to 0.41)
San Marino	27.6 (19.8 to 36.8)	101.4 (72.6 to 135.3)	45.5 (33.0 to 60.2)	108.6 (78.4 to 144.2)	0.22 (0.21 to 0.22)
Sao Tome and Principe	17.8 (12.6 to 24.1)	18.0 (12.6 to 24.5)	39.1 (27.4 to 53.1)	20.0 (14.3 to 27.1)	0.33 (0.3 to 0.35)

Saudi Arabia	4731.5 (3368.8 to 6455.5)	37.9 (27.0 to 51.0)	20085.0 (14133.4 to 27170.5)	49.9 (35.1 to 67.4)	0.89 (0.88 to 0.9)
Senegal	984.9 (697.7 to 1296.8)	16.3 (11.8 to 21.3)	2436.9 (1776.4 to 3303.8)	17.9 (13.2 to 24.1)	0.3 (0.28 to 0.32)
Serbia	3756.2 (2664.1 to 4973.3)	34.9 (24.7 to 46.5)	4490.8 (3208.5 to 5918.6)	41.1 (29.1 to 54.1)	0.52 (0.51 to 0.53)
Seychelles	29.7 (21.1 to 39.4)	45.5 (32.2 to 60.0)	61.3 (44.2 to 82.4)	52.3 (38.0 to 70.0)	0.46 (0.45 to 0.47)
Sierra Leone	555.3 (387.9 to 753.2)	16.3 (11.5 to 21.8)	1300.4 (915.3 to 1749.7)	17.3 (12.4 to 23.3)	0.2 (0.18 to 0.22)
Singapore	1055.7 (751.9 to 1420.2)	34.2 (24.5 to 45.6)	2751.4 (1954.2 to 3648.8)	38.8 (27.4 to 51.3)	0.38 (0.37 to 0.4)
Slovakia	1943.0 (1406.2 to 2543.1)	34.8 (25.3 to 45.8)	2728.2 (1986.1 to 3613.6)	41.0 (29.5 to 54.6)	0.53 (0.53 to 0.54)
Slovenia	812.9 (596.0 to 1094.7)	37.1 (27.2 to 50.1)	1103.6 (796.4 to 1475.7)	42.7 (31.1 to 57.1)	0.46 (0.44 to 0.47)
Solomon Islands	67.9 (48.1 to 93.9)	27.2 (19.7 to 37.0)	178.4 (126.3 to 240.5)	31.3 (22.4 to 42.1)	0.45 (0.44 to 0.46)
Somalia	659.2 (475.5 to 876.7)	10.8 (7.7 to 14.2)	1664.4 (1164.7 to 2276.7)	10.0 (7.1 to 13.5)	-0.24 (-0.25 to -0.22)
South Africa	6593.5 (4722.7 to 8900.3)	20.3 (14.6 to 27.2)	12171.0 (8728.0 to 16250.2)	21.3 (15.3 to 28.5)	0.16 (0.15 to 0.16)

South Sudan	566.9 (406.0 to 761.6)	12.0 (8.6 to 16.0)	902.7 (641.0 to 1217.9)	11.4 (8.0 to 15.3)	-0.17 (-0.18 to -0.16)
Spain	25246.2 (18203.5 to 33583.0)	58.3 (41.9 to 77.7)	36353.2 (26638.7 to 48530.4)	63.6 (46.2 to 84.2)	0.28 (0.27 to 0.29)
Sri Lanka	6525.7 (4713.4 to 8719.4)	42.4 (30.7 to 56.1)	12403.7 (9080.8 to 16247.4)	50.3 (37.0 to 66.2)	0.56 (0.54 to 0.57)
Sudan	4771.5 (3359.5 to 6603.9)	30.9 (22.0 to 42.4)	14634.8 (10465.1 to 19875.4)	40.4 (28.9 to 54.6)	0.87 (0.85 to 0.89)
Suriname	187.9 (134.3 to 254.8)	53.3 (38.4 to 72.6)	351.4 (255.6 to 469.3)	57.3 (41.7 to 76.5)	0.24 (0.23 to 0.25)
Sweden	6931.7 (4980.1 to 9160.4)	69.6 (49.8 to 92.2)	8897.9 (6523.5 to 11963.7)	71.9 (52.8 to 98.0)	0.09 (0.09 to 0.1)
Switzerland	8266.9 (5973.0 to 10932.3)	103.7 (75.3 to 137.1)	12461.3 (9005.3 to 16677.2)	112.1 (81.0 to 149.9)	0.25 (0.24 to 0.25)
Syrian Arab Republic	3097.8 (2198.8 to 4172.9)	32.8 (23.6 to 43.7)	5987.3 (4279.9 to 8026.0)	41.3 (29.7 to 55.6)	0.75 (0.74 to 0.76)
Taiwan (Province of China)	3500.6 (2507.1 to 4725.1)	17.8 (12.7 to 23.9)	7009.3 (5111.6 to 9332.1)	22.7 (16.5 to 30.1)	0.79 (0.77 to 0.81)
Tajikistan	1287.5 (922.4 to 1720.7)	30.4 (21.9 to 40.5)	2988.4 (2137.3 to 3990.1)	32.9 (23.5 to 44.1)	0.26 (0.25 to 0.27)
Thailand	20945.4 (15045.4 to 28048.6)	40.6 (29.3 to 53.9)	40793.7 (29663.3 to 54611.1)	48.2 (34.7 to 64.6)	0.56 (0.55 to 0.57)

Timor-Leste	197.8 (140.3 to 268.4)	33.6 (24.4 to 45.1)	501.9 (365.9 to 677.0)	42.8 (31.2 to 57.4)	0.78 (0.77 to 0.79)
Togo	483.4 (345.0 to 638.4)	17.0 (12.2 to 22.7)	1331.4 (958.6 to 1826.4)	18.3 (13.2 to 24.9)	0.21 (0.18 to 0.24)
Tokelau	0.4 (0.3 to 0.6)	31.1 (22.1 to 42.1)	0.5 (0.4 to 0.7)	38.1 (27.8 to 50.6)	0.65 (0.64 to 0.67)
Tonga	25.2 (17.5 to 33.8)	32.0 (22.4 to 42.7)	34.7 (24.6 to 46.5)	36.8 (26.1 to 49.5)	0.45 (0.44 to 0.46)
Trinidad and Tobago	642.3 (460.7 to 851.5)	57.8 (41.6 to 76.7)	1001.9 (710.9 to 1326.7)	63.2 (44.7 to 83.4)	0.29 (0.28 to 0.3)
Tunisia	2491.9 (1828.1 to 3364.3)	34.9 (25.6 to 47.1)	5592.5 (3984.7 to 7505.1)	43.1 (30.7 to 57.7)	0.68 (0.66 to 0.69)
Turkey	23252.9 (16414.9 to 30882.3)	46.6 (33.1 to 61.9)	55689.2 (40382.6 to 74009.5)	60.6 (44.0 to 80.8)	0.85 (0.84 to 0.86)
Turkmenistan	984.6 (697.9 to 1329.3)	32.6 (23.1 to 43.7)	1868.2 (1368.2 to 2504.0)	37.0 (27.1 to 49.5)	0.4 (0.39 to 0.41)
Tuvalu	2.6 (1.9 to 3.4)	30.8 (22.2 to 40.9)	4.4 (3.1 to 5.9)	37.0 (26.4 to 49.9)	0.59 (0.58 to 0.6)
Uganda	1558.2 (1092.5 to 2115.0)	11.8 (8.5 to 16.0)	4345.5 (3064.6 to 5977.0)	12.8 (9.2 to 17.3)	0.25 (0.24 to 0.26)
Ukraine	19924.1 (14458.0 to 26646.8)	33.5 (24.4 to 44.8)	19796.8 (14461.3 to 26416.4)	36.9 (26.6 to 49.4)	0.32 (0.31 to 0.33)

United Arab Emirates	698.1 (494.5 to 945.0)	41.8 (30.3 to 56.0)	6195.1 (4347.3 to 8376.2)	52.3 (37.5 to 69.5)	0.73 (0.72 to 0.74)
United Kingdom	51143.2 (37166.3 to 68564.1)	78.6 (56.8 to 105.3)	73776.2 (53401.2 to 98325.0)	90.2 (65.3 to 120.6)	0.44 (0.43 to 0.45)
United Republic of Tanzania	2625.8 (1834.0 to 3462.5)	13.0 (9.1 to 17.1)	6473.9 (4629.6 to 8869.5)	13.4 (9.5 to 18.1)	0.1 (0.08 to 0.12)
United States of America	220663.6 (160362.5 to 294717.3)	81.4 (59.0 to 108.7)	326749.5 (239297.8 to 431398.0)	83.6 (60.9 to 110.7)	0.09 (0.08 to 0.09)
United States Virgin Islands	62.5 (45.4 to 82.9)	59.9 (43.4 to 79.1)	68.9 (50.4 to 90.7)	64.5 (46.5 to 85.9)	0.24 (0.23 to 0.24)
Uruguay	1534.7 (1117.2 to 2019.1)	46.5 (33.7 to 61.4)	1991.1 (1423.6 to 2641.0)	51.5 (37.1 to 68.8)	0.34 (0.34 to 0.35)
Uzbekistan	5199.2 (3755.8 to 6903.0)	30.4 (22.0 to 40.1)	11909.1 (8469.9 to 15747.5)	35.4 (25.2 to 46.7)	0.5 (0.49 to 0.51)
Vanuatu	32.5 (23.2 to 43.7)	28.5 (20.3 to 38.1)	86.6 (62.0 to 116.3)	32.5 (23.6 to 43.4)	0.43 (0.42 to 0.44)
Venezuela (Bolivarian Republic of)	8673.4 (6186.7 to 11559.1)	53.5 (38.0 to 70.9)	15998.0 (11723.7 to 21332.9)	56.2 (41.1 to 75.1)	0.16 (0.15 to 0.17)
Viet Nam	20535.8 (14938.9 to 27873.5)	36.9 (26.7 to 49.6)	49017.5 (35649.6 to 65922.0)	46.2 (33.6 to 62.1)	0.73 (0.72 to 0.74)
Yemen	2850.0 (2039.9 to 3794.3)	29.5 (21.1 to 39.2)	10260.5 (7458.8 to 13744.5)	37.4 (27.3 to 49.8)	0.77 (0.76 to 0.78)

Zambia	772.4 (527.4 to 1034.8)	12.7 (8.8 to 16.9)	2093.2 (1501.8 to 2858.2)	13.2 (9.5 to 17.6)	0.12 (0.1 to 0.13)
Zimbabwe	1515.3 (1047.3 to 2010.9)	18.4 (13.1 to 24.1)	2496.7 (1758.6 to 3352.5)	18.7 (13.3 to 24.8)	0.04 (0.02 to 0.06)

Abbreviations: ASR: age-standardized rate; AAPC: average annual percentage change; CI: confidence interval; UI: uncertainty interval.

Supplement Figure 1. Map of the average annual percentage change of psoriasis from 1990 to 2021. (A) incidence; (B) mortality; (C) disability-adjusted life years.

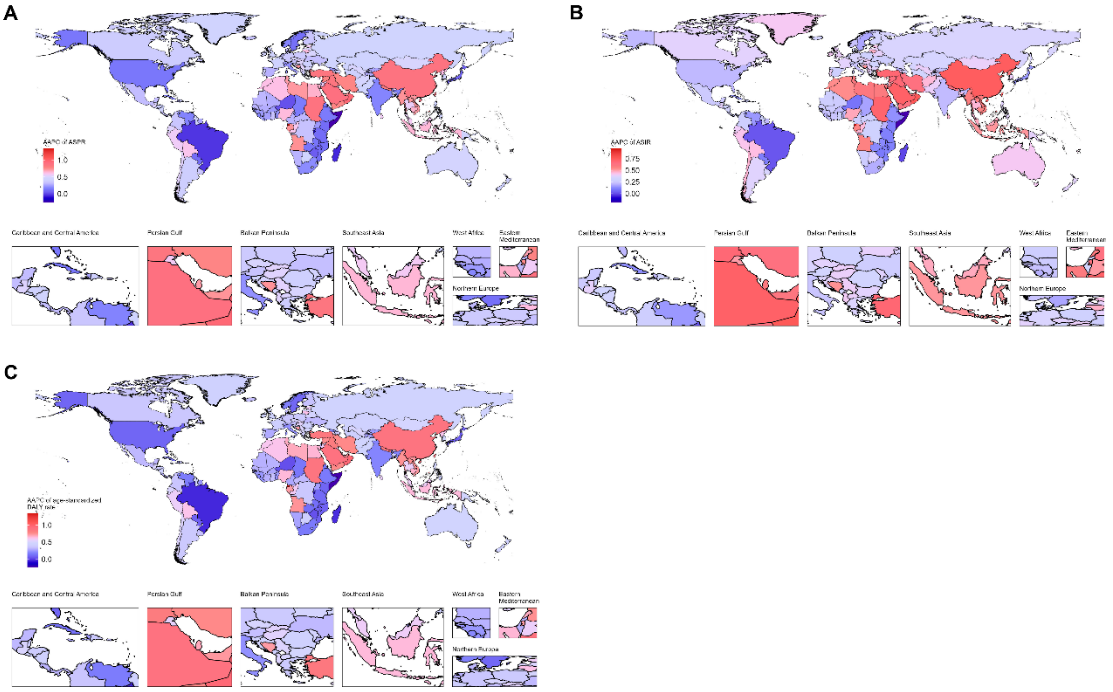

Supplementary Figure 2. The age-specific numbers and age-standardized incidence rate of psoriasis by SDI regions in 2021.

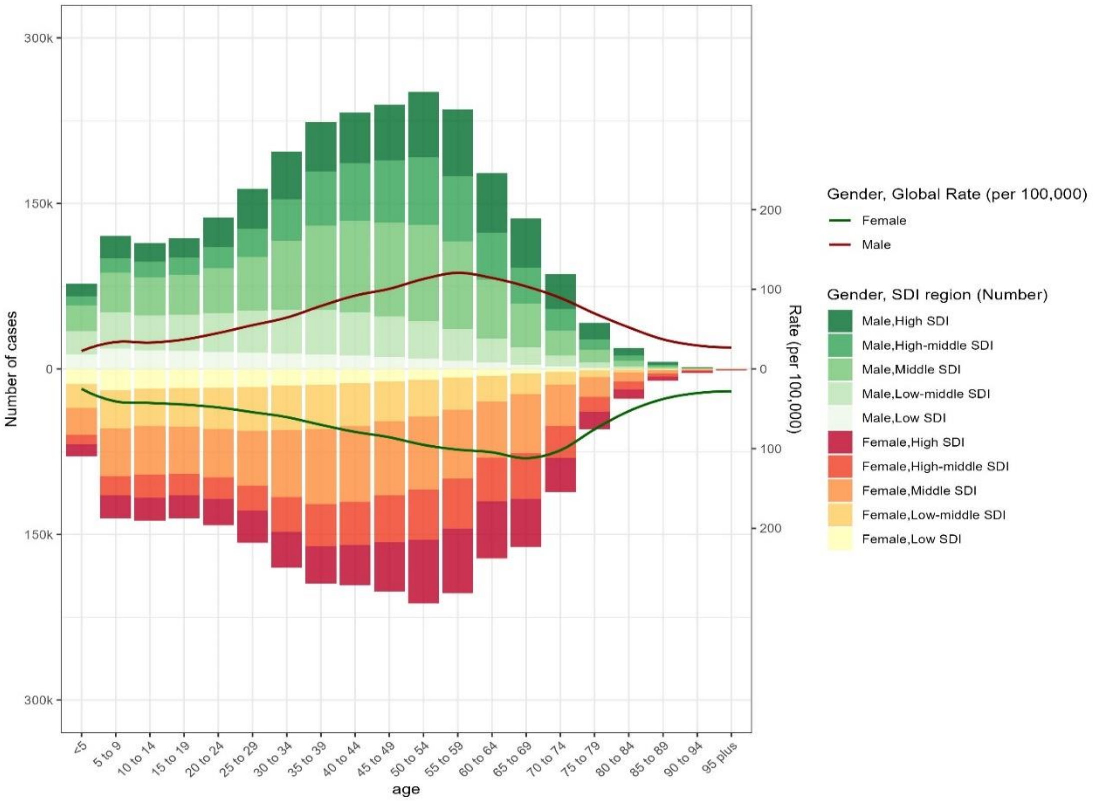

Supplementary Figure 3. The age-specific numbers and age-standardized DALY rate of psoriasis by SDI regions in 2021.

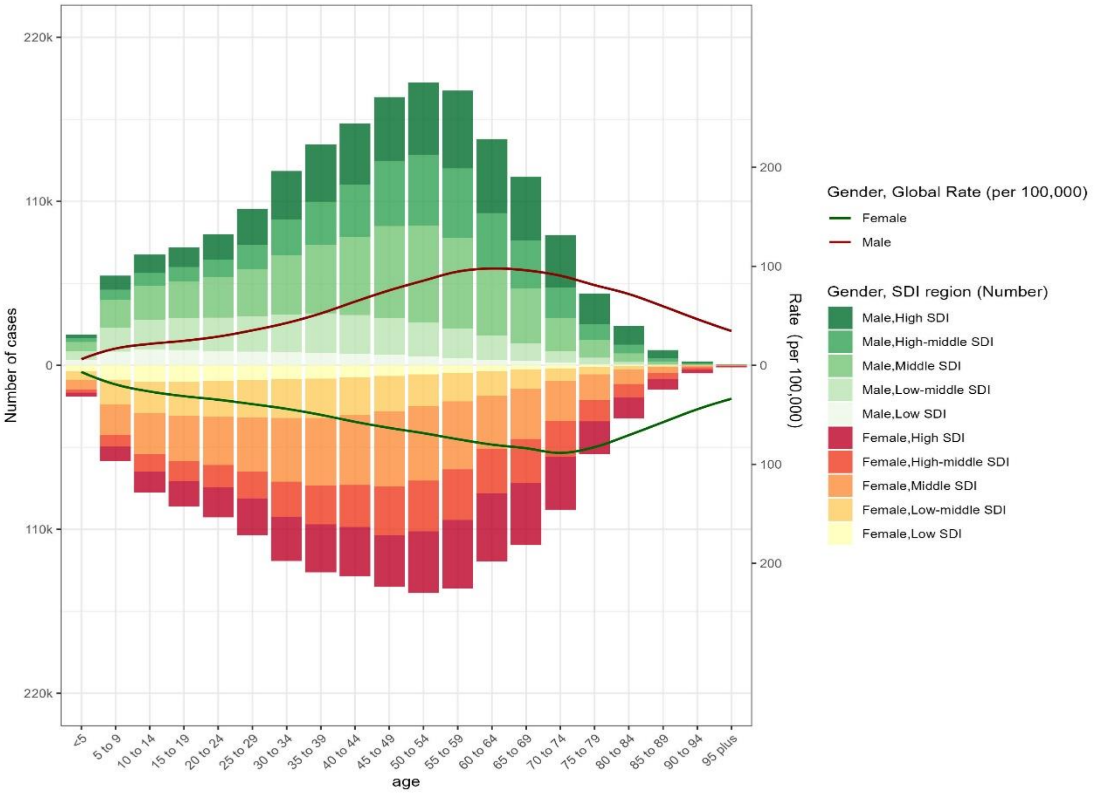

Supplementary Figure 4. Age-standardized incidence rates of psoriasis by sex, age group, and socio-demographic index, 1990 and 2021.

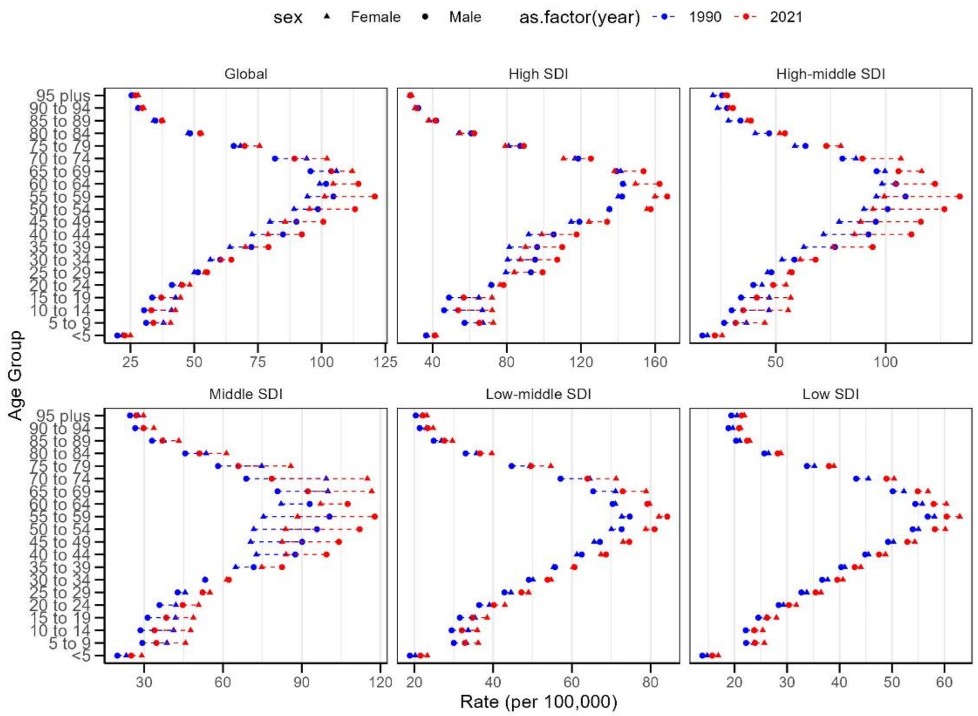

Supplementary Figure 5. Age-standardized DALY rates of psoriasis by sex, age group, and socio-demographic index, 1990 and 2021.

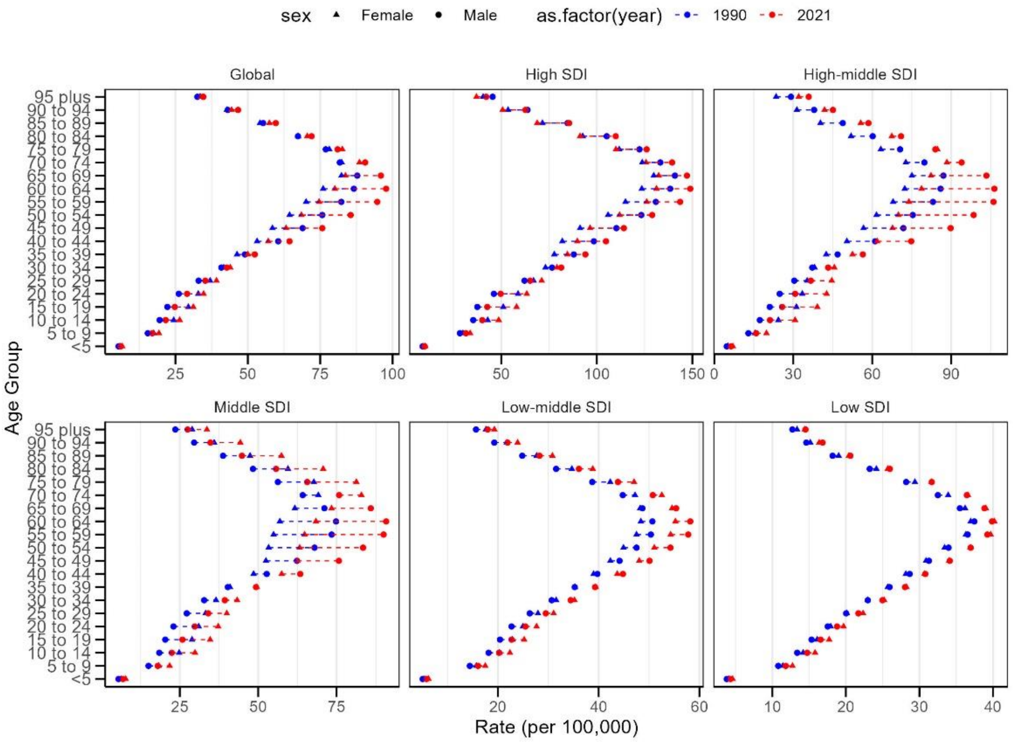

Supplementary Figure 6. The DALY rates for psoriasis were classified according to the SDI across 204 countries in 2021.

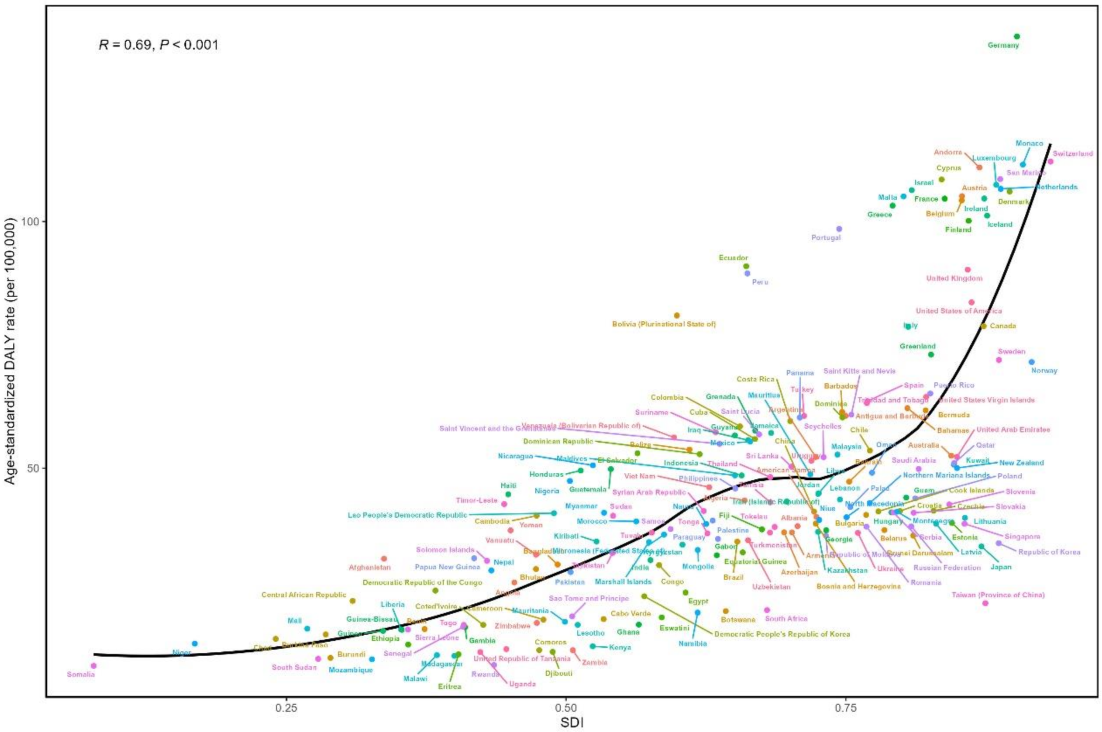

Supplement: Supplementary file 1 [file Data_Sheet_1.pdf]
